# Geographic–genomic and geographic–phenotypic differentiation of the *Aquilegia viridiflora* complex

**DOI:** 10.1093/hr/uhad041

**Published:** 2023-03-13

**Authors:** Wei Zhang, Huaying Wang, Tengjiao Zhang, Xiaoxue Fang, Meiying Liu, Hongxing Xiao

**Affiliations:** Key Laboratory of Molecular Epigenetics of Ministry of Education, College of Life Sciences, Northeast Normal University, Changchun 130024, China; Key Laboratory of Molecular Epigenetics of Ministry of Education, College of Life Sciences, Northeast Normal University, Changchun 130024, China; Key Laboratory of Molecular Epigenetics of Ministry of Education, College of Life Sciences, Northeast Normal University, Changchun 130024, China; Key Laboratory of Molecular Epigenetics of Ministry of Education, College of Life Sciences, Northeast Normal University, Changchun 130024, China; Key Laboratory of Molecular Epigenetics of Ministry of Education, College of Life Sciences, Northeast Normal University, Changchun 130024, China; Key Laboratory of Molecular Epigenetics of Ministry of Education, College of Life Sciences, Northeast Normal University, Changchun 130024, China

## Abstract

How species diverge into different lineages is a central issue in evolutionary biology. Despite the increasing evidence indicating that such divergences do not need geographic isolation, the correlation between lineage divergence and the adaptive ecological divergence of phenotype corresponding to distribution is still unknown. In addition, gene flow has been widely detected during and through such diverging processes. We used one widely distributed *Aquilegia viridiflora* complex as a model system to examine genomic differentiation and corresponding phenotypic variations along geographic gradients. Our phenotypic analyses of 20 populations from northwest to northeast China identified two phenotypic groups along the geographic cline. All examined traits are distinct from each other, although a few intermediate individuals occur in their contacting regions. We further sequenced the genomes of representative individuals of each population. However, four distinct genetic lineages were detected based on nuclear genomes. In particular, we recovered numerous genetic hybrids in the contact regions of four lineages. Gene flow is widespread and continuous between four lineages but much higher between contacting lineages than geographically isolated lineages. Gene flow and natural selection might result in inconsistency between heredity and phenotype. Moreover, many genes with fast lineage-specific mutations were identified to be involved in local adaptation. Our results suggest that both geographic isolation and local selection exerted by the environment and pollinators may together create geographic distributions of phenotypic variations as well as the underlying genomic divergences in numerous lineages.

## Introduction

Disentangling the drivers of genomic and phenotypic divergence is essential to the understanding of speciation processes. Allopatric divergence has been considered the most likely cause of speciation for a long time, and targets of natural selection may contribute to allopatric speciation when populations encounter different selective pressures in different habitats [1]. One of the key speciation forces is evolutionary divergence driven by adaptation to the environment, essentially proposed by Charles Darwin (1859) . At the genomic level, loci with strong population differentiation reflect local adaptation in populations under different environments, in which reproductive isolation may appear as a byproduct of the accumulation of genetic differences [2–4]. Natural selection thus acts as a barrier to gene flow to produce local genomic divergence between lineages [5–7]. Similarly, gene flow and drift can also act and even interact in several ways during evolutionary divergence [8, 9]. Geographic patterns of phenotype divergence also reflect the fact that that traits have diverged with environmental gradients as selective forces and created adaptive genetic differences [10–12]. Extensive studies have indicated that morphological divergence is expected to be associated with prezygotic reproductive barriers. Plants can directly or indirectly reduce matting, sperm transfer, or fertilization [13, 14] by changing both the flowering time and mating system. For many taxa, including *Aquilegia formosa* and *Aquilegia pubescens*, the adaptive ecological divergence of floral characteristics mediated by pollinators likely contributes toreproductive isolation between populations [15–19]. If populations with divergent selection maintain geographic isolation,environmental differences may drive lineage adaptation and differentiation and act to form reproductive isolation between them with restricted gene flow. However, the latest research suggests that recently speciated and speciating allopatric taxa seem to evolve under similar selective pressures [20]. This denies the classical view that the main driver of the early stages of speciation is divergent adaptation. Consequently, species withwide distribution provide a model to infer the effect of divergent ecological pressures or similar ecological pressure on allopatric lineages.

During population divergence, driving forces may produce distinct demographic histories, including divergence time, population size fluctuations, and directionality of gene flow. However, itcan be difficult to identify which force is likely to have conducedto the current phylogeographic pattern of a certain species. The identification of highly differentiated regions in the genome may have false-positive results due to the influence of the unique demographic histories [21]. Additionally, interspecific gene flowoften leads to adaptive introgression and contributes to the development of adaptive floral traits [22, 23]. Intraspecific gene flow might homogenize diverging populations and delay speciation [24]. Therefore, it is important to reconstruct the past demographyof lineages and reliably estimate interspecific and intraspecific gene flow. Moreover, East Asia occupies a diversiform climatic and geographic environment and is considered a natural laboratory for the study of adaptive evolution [25]. Evidence from previous studies in this region has uncovered the demographic histories, genetic diversities, and related influencing factors of many taxa [26–28]. While the heterogeneous environment of this area might have contributed to lineage divergence, it is still uncertain what might be the key driver.

**Figure 1:**
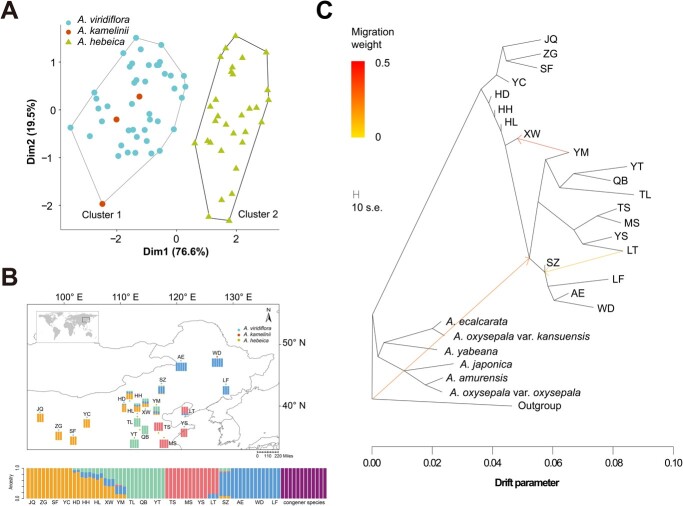
Sampling localities and population genetic structure of the *A. viridiflora* complex. (A) *K*-means cluster analysis of phenotype based on the first two principal components. (B) Geographic distributions of the sampled *A. viridiflora* complex, where colors in circles distinguish groups. The genetic structure showed ADMIXTURE proportions of genetic clusters for each individual at the best *K* value (*K* = 5), and each bar represents an individual. The scale in the figure is 1:20 000 000. (C) Three gene flow events of *Aquilegia*. Each branch represents a population, and arrows indicate migration events that occur between populations.

*A. viridiflora* Pall. (Ranunculaceae) is a dominant, perennial herb that is widely distributed in northern China with obvious variation in phenotypes [29]. In previous studies, Andrey S. Erst *et al*. identified *A. viridiflora* with purple laminae or lilac-blue petals as the new species *A. kamelinii* A. Erst, Schaulo et Schmakov [30] and *A. viridiflora* with dark purple calyx in North China as the new species *A. hebeica* Erst [31]. However, the publication of these new species lacks support from molecular data. According to field surveys, the phenotypic variation of *A. viridiflora* is continuous (Supplementary Data Fig. S1) and presents substantial challenges in species delimitation. Therefore, *A. viridiflora*, *A. kamelinii*, and *A. hebeica* were considered the species complex (*A. viridiflora* complex) in our study. In addition, *Aquilegia* is a well-known example in evolutionary biology [32]. Phylogenetic studies have defined adaptive radiation in *Aquilegia*, involving a wide variety of habitats, and divergent selection has played an important role in the radiation of *Aquilegia* [33–35]. Therefore, the *A. viridiflora* complex provides a remarkable system for assessing how evolutionary forces in various ways could have shaped genomic divergence patterns during speciation.

In the present study, we collected population samples covering the main distribution range of the *A. viridiflora* complex for genome resequencing and morphological characteristic measurements. Then, we assumed that environmental heterogeneity and pollinator insect assemblages may put selective pressures on the *A. viridiflora* complex, driving population divergence and producing genetic variation. Thus, our study focuses on exploring phenotypic and genomic patterns of divergence, and especially on inferring the influences of gene flow, genetic drift, divergence time, divergent selection, and geographic isolation during the speciation process. The investigation reveals the contribution of evolutionary forces to the genomic divergence patterns of the *A. viridiflora* complex and helps us understand allopatric speciation.

**Table 1:** Results of *Q*_ST_–*F*_ST_ analysis.

	***Q***_**ST**_ **(CI)**	***F***_**ST**_ **(CI)**	***P* (ANOVA)**
**Corolla diameter**	0.90634 (-0.8861 to 1.80838)	0.15019 (0.14949 to 0.15074)	<2e−16^***^
**Petal length**	0.42502 (0.02978 to 0.79713)	0.15019 (0.14949 to 0.15074)	<2e−16^***^
**Spur length**	0.52134 (0.01099 to 0.84502)	0.15019 (0.14949 to 0.15074)	<2e−16^***^
**Pistil length**	0.29603 (0.00279 to 0.63464)	0.15019 (0.14949 to 0.15074)	1.84e−12^***^
Stamen length	0.01293 (-0.032 to 0.19406)	0.15019 (0.14949 to 0.15074)	0.00516^**^
Calyx length	0.01272 (-0.0327 to 0.17102)	0.15019 (0.14949 to 0.15074)	0.0176^*^
**Inflorescence number**	0.26357 (0.0309 to 0.64785)	0.15019 (0.14949 to 0.15074)	3.24e−06^***^
**Leaf area**	0.21814 (-0.03151 to 0.68805)	0.15019 (0.14949 to 0.15074)	4.12e−08^***^
Chlorophyll content	0.10519 (-0.2486 to 1.53042)	0.15019 (0.14949 to 0.15074)	0.006^**^
Leaf perimeter	0.14503 (-0.0132 to 0.57413)	0.15019 (0.14949 to 0.15074)	5.15e−05^***^
Height	0.11485 (-0.0264 to 0.38348)	0.15019 (0.14949 to 0.15074)	1.61e−07^***^

^*^Significant difference between different phenotypes: .01 < *p* < .05.

^**^Significant difference between different phenotypes: .001 < *p* < .01.

^***^Significant difference between different phenotypes: *p* < .001.

Phenotypes with adaptation are indicated in bold.

## Results

### Phenotypic variation across the distribution

We sampled 20 populations of the *A. viridiflora* complex with various phenotypes that covered its entire distribution (Fig. 1B, Supplementary Data Fig. S1, Supplementary Data Table S1). The phenotypes of *A. viridiflora* complex populations grown in common gardens were investigated and compared, and each phenotype differed significantly among different populations by using ANOVA (Table 1). *K*-means cluster analysis was used to visualize the phenotypic variation among populations and identified two distinctive groups. Dim1 and Dim2 could explain 76.6 and 19.5% of the observed variation, respectively, and the cumulative contribution to the observed phenotypic variation was 96.1%. All individuals of *A. viridiflora* and *A. kamelinii* were found in cluster 1, while individuals of *A. hebeica* were found in cluster 2 (Fig. 1A). There was a significantly negative correlation coefficient between some floral characteristics, including corolla length, petal length, spur length and pistil length, and the nutritional traits, including leaf area, leaf perimeter and height. In addition, the number of inflorescences was significantly negatively correlated with the above floral characteristics, while it was significantly positively correlated with plant height (Supplementary Data Fig. S2).

### Interspecific and intraspecific genetic structure

**Figure 2:**
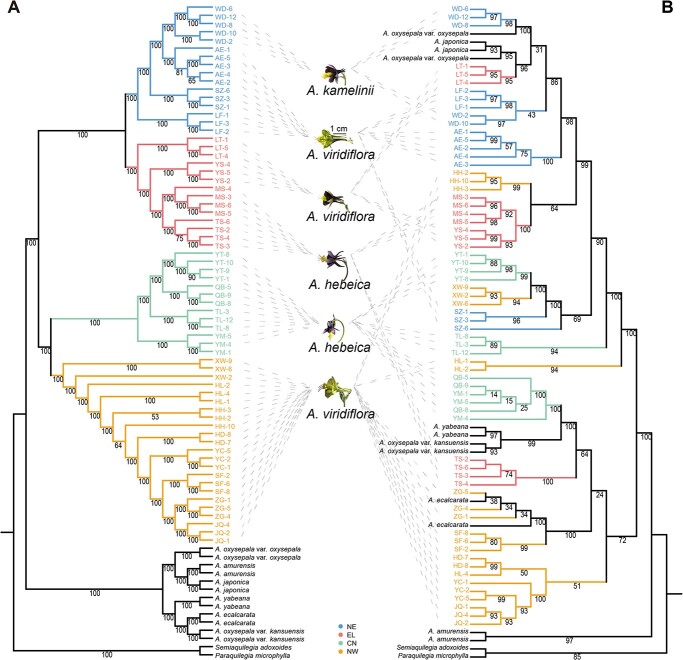
Phylogenetic relationship between *A. viridiflora* complex and other columbine species whose distribution overlapped with *A. viridiflora* complex. (A) Nuclear genome. (B) Chloroplast genome.

To ensure that the species complex with various phenotypes could be regarded as a monophyletic group, we also sampled other sympatric wild columbine species with the *A. viridiflora* complex. Whole-genome resequencing of the 80 individuals generated a total of 392 Gb of data, with an average of 5 Gb and corresponding to ~16× depth for each individual (Supplementary Data Table S2). We constructed the phylogenetic relationships among the *Aquilegia* species through ML methods based on nuclear genome SNPs. The topologies indicated that *A. viridiflora*, *A. kamelinii*, and *A. hebeica* shared an MRCA with strong support and the individuals of *A. viridiflora* complex were divided into two groups (East and West) (Fig. 2A). The population genetic structure of *Aquilegia* species indicated that ancestral clustering at *K* = 5 was optimal according to the cross-validation error rate (Supplementary Data Fig. S3). No admixture between *A. viridiflora* complex and other *Aquilegia* species was apparent from the ADMIXTURE analysis (Fig. 1B). To examine the gene flow between *A. viridiflora* complex and other *Aquilegia* species, we then performed an ABBA-BABA test. We found no significant deviation of the *D*-statistic from zero, indicating that gene flow did not exist between *A. viridiflora* complex and other *Aquilegia* species (Supplementary Data Table S3). Additionally, the optimal number of migration events in Treemix was found to be three. In this model of Treemix analysis it was inferred that gene flow or admixture also did not exist between *A. viridiflora* complex and other *Aquilegia* species (Fig. 1C, Supplementary Data Fig. S4).

**Figure 3:**
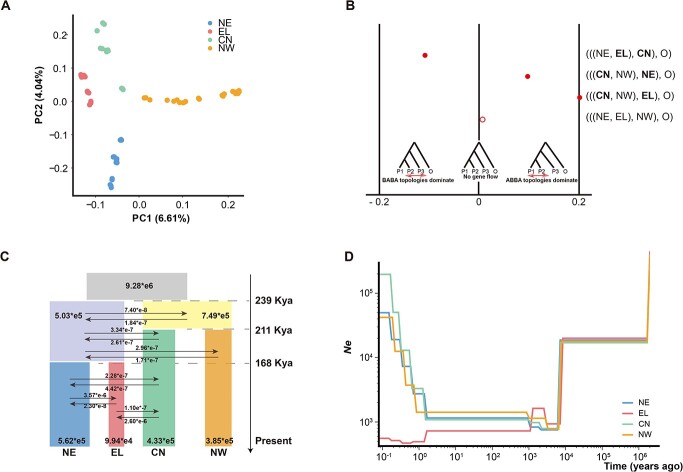
Demographic history of *A. viridiflora* complex and population genetic analysis. (A) PCA plot for the 66 *A. viridiflora* complex individuals based on the first two principal components. (B) Results for the *D*-statistics. The solid red dot indicates that the absolute value of *Z*-scores is > 3, and the open red dot indicates absolute value of *Z*-scores < 3. (C) Optimal demographic scenario using fastsimcoal2, ancestral population sizes, population divergence times, and migration rates were assessed. The 95% CIs of split times, population size, and migration rates obtained from this model are shown in Supplementary Data Table S9. (D) Changes in effective population size inferred by SMC++ through time.

The ML tree inferred from the above SNPs indicated that the individuals of the *A. viridiflora* complex were divided into four lineages (NE, EL, CN, and NW): NE comprised *A. kamelinii* and individuals of *A. viridiflora* distributed in northeastern China; EL comprised individuals of *A. viridiflora* and *A. hebeica* distributed in East Shandong South Liaoning area; individuals of *A. hebeica* distributed in North China belonged to a single lineage (EL); and individuals of *A. viridiflora* distributed in northwestern China belongedto a single lineage (NW). In this case, the *A. viridiflora* complex showed a paraphyletic pattern, i.e. NE and EL formed a sister clade, and the other two lineages, CN and NW, were closely related (Fig. 2A). This revealed a different evolutionary history from the clusters based on phenotypes. The result of ancestral inference was obviously consistent with the geographic distribution of the 20 populations and the phylogenetic relationships detailed above. Individuals of the SZ, LT, YM, XW, HL, HH, and HD populations in the contact regions of lineages showed multiple ancestral compositions (Figs 1B and 2A), which might reflect recent gene flow between these lineages. From the PCA plot, the first principal component (PC1) and second principal component (PC2) explained 6.61 and 4.04% of the observed variation, respectively. It also showed four distinct lineages among the 20 populations, while individuals with multiple ancestral compositions occupied an intermediate space in distinct lineages (Fig. 3A). The NeighborNet phylogenetic network depicted these patterns by grouping the differential of four lineages; it was also proven that the above individuals in contact regions had a mixed genetic background (Supplementary Data Fig. S5). In addition, we also detected a significant signal of hybridization in the above populations in contact regions at the individual level (Supplementary Data Table S4).

### Chloroplast–nuclear discordance

Unsurprisingly, the chloroplast genome phylogenetic tree showed a different topology from the phylogenetic tree of the nuclear genome, with lower bootstrap support, based on 257 polymorphic sites, which could not recognize the relationship and species boundaries in *Aquilegia*. *A. kamelinii* (WD population) and *A. oxysepala* var. *oxysepala* formed a monophyletic group with a bootstrap value of 100%, *A. viridiflora* (LT population), *A. oxysepala* var. *oxysepala*, and *A. japonica* formed a monophyletic group with a bootstrap value of 96%, *A. hebeica* (QB and YM populations), *A. oxysepala* var. *kansuensis*, and *A. yabeana* formed a monophyletic group with a bootstrap value of 100%, and *A. viridiflora* (ZG population) and *A. ecalcarata* formed a monophyletic group with a bootstrap value of 100% (Fig. 2B). Neither the chloroplast nor the nuclear genome tree showed a topology consistent with the cluster based on phenotypes. The NeighborNet phylogenetic network of *A. viridiflora* complex in the chloroplast genome showed a paraphyletic pattern without geographic structure (Supplementary Data Fig. S6A). Based on chloroplast genome polymorphisms, we detected 27 haplotypes among the 66 *A. viridiflora* complex individuals. The haplotype diversity and nucleotide diversity (π) for all individuals were 0.971 and 0.00022, respectively. Among the four lineages based on the nuclear genome, NW had the highest haplotype diversity and nucleotide diversity (Supplementary Data Table S5). Moreover, the haplotypes in NW were in this network elsewhere, while the haplotypes in the other groups were limited in the haplotype network (Supplementary Data Fig. S6B, Supplementary Data Table S6).

### Tests of gene flow and dynamic history inference

To investigate gene flow, effective population size (*N*_e_) and divergence time, 46 individuals were retained after removal of hybrids. We first examined the gene flow between lineages through the ABBA-BABA test. For four lineages, {[(NE, EL), CN], outgroup}, {[(CN, NW), NE], outgroup} and {[(CN, NW), EL], outgroup} showed a significant deviation of *D*-statistics from zero (absolute value of *Z*-score >3), indicating that gene flow exists between lineages EL and CN, CN and NE, and CN and EL. Interestingly, no gene flow was detected between the NW lineage and other lineages (Fig. 3B and Supplementary Data Table S7). Next, we employed Treemix to further investigate the complex patterns of gene flow between populations. The results of Treemix analysis indicated that recent gene flow was only exhibited in the EL and NE lineages, while historical gene flow was exhibited in the NE, EL, and CN lineages (Supplementary Data Fig. S7).

Combined with the results of the ABBA-BABA test and Treemix analysis, the evolutionary history of the four lineages was inferred by fastsimicoal2 using pairwise joint site frequency spectra. Based on the best-supported model (Supplementary Data Fig. S8, Supplementary Data Table S8), the effective population size was 927 589 for the ancestors. The ancestral population was differentiated into eastern and western lineages at ~239 Kya [95% highest posterior density (HPD) = 217 - 267 Kya], and their effective population sizes were 385 321 and 433 093, respectively. Next, CN and NW separated at ~211 Kya (95% HPD = 196 - 227 Kya), and NE and EL separated at 168 Kya (95% HPD = 153 - 184 Kya). All the divergence times were in the Middle Pleistocene. The current effective population sizes of NE, EL, CN, and NW were 561 674, 99 440, 433 094, and 385 321, respectively (Fig. 3C, Supplementary Data Table S9). In addition, the divergence of the four lineages accompanied six bidirectional gene flow events inferred by fastsimcoal2, including three ancient gene flows and three modern gene flows. The modern gene flow was higher from NE to EL than others (CN to EL > CN to NE > EL to NE > NE to CN > EL to CN), which indicated gene flow between contacting lineages larger than the geographically isolated ones. Although no recent gene flow was detected between NW and the other three lineages, hybrids formed by NE, CN, and NW lineage mixing still arose, which resulted from the ancient gene flow not only between the ancestral clade of the NE and EL lineages and the NW lineage, but also between the ancestral clade of the NE and EL lineages and the ancestral clade of the CN and NW lineages. The credible lineage divergence pattern had a better fit based on the comparison of the simulated dataset with the observed site frequency spectra (Supplementary Data Fig. S9). SMC++ analyses revealed that the effective population sizes of four lineages of *A. viridiflora* complex declined at the Last Glacial Maximum (LGM, ~11.7 thousand years) (Fig. 3D). This result suggested that long-term climate changes impacted the fluctuations in effective population sizes.

**Figure 4:**
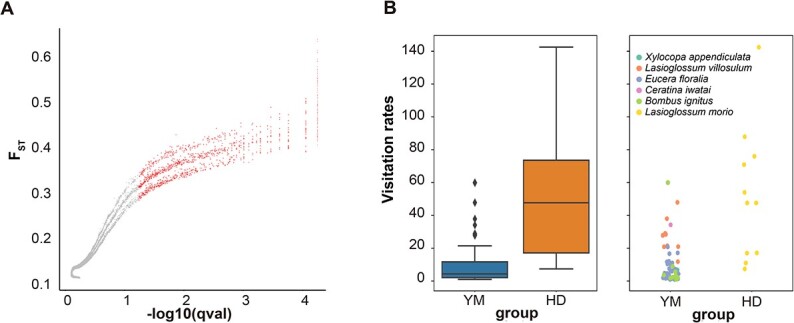
Divergent ecological pressures promoting genomic region differentiation. (A) BayeScan results for genome-wide scans to detect outlier SNPs based on locus-specific *F*_ST_ values. Red dots represent qval < 0.05 and indicate SNPs under positive selection. (B) Visitation rates by pollinators in different study populations.

To understand the diversity patterns, the nucleotide diversity (π) of the NE, EL, CN, and NW lineages was calculated throughout the genome for each 50 kb with a 10-kb step size. Among the four lineages, NW showed the highest nucleotide diversity and EL showed the lowest nucleotide polymorphism (Supplementary Data Fig. S10A). *F*_ST_ was calculated across different lineages (NE, EL, CN, and NW) to infer population genetic differentiation. At the overall level of the genome, the *F*_ST_ values had the highest value between EL and NW, which indicated that the *A. viridiflora* complex had a moderate level of differentiation. Lineage NW was the most differentiated from the other lineages, possibly due to the lack of recent gene flow. This pattern was also obvious from the *F*_ST_ and *D*_XY_ values calculated for the four lineages using 10-kb windows across genomes, which were consistent with *F*_ST_ at the overall level of the genome (Supplementary Data Fig. S10B). We compared LD decay between different chromosomes of four lineages. The analysis showed that CN and EL presented a greater degree of LD, while NE and NW showed less LD (indicated by *r*^2^). When *r*^2^ = .1, the decay distances of NE, EL, CN, and NW were 10, 37, 44 and 6.9 kb, respectively (Supplementary Data Fig. S10C). The rapid decay of NE and NW may have been due to the higher genetic diversity relative to that of EL and CN.

### Local phenotypic adaptation and genes under selection

Common garden experiments were conducted to investigate the phenotypic divergence evidence for genome differentiation and adaptation of the *A. viridiflora* complex. The mean *F*_ST_ for the four lineages was 0.15019 (95% HPD 0.14949 - 0.15074), and the overall *Q*_ST_ of the six traits was higher than the mean *F*_ST_, including corolla diameter, petal length, spur length, pistil length, inflorescence number, and leaf area, indicating that these traits was driven by local adaptation (Table 1). The highest *Q*_ST_ was obtained by measuring corolla diameter, followed by spur length, petal length, pistil length, inflorescence number, and leaf area, which also showed a higher *Q*_ST_ of floral characteristics than nutritional traits. Among the four lineages, CN showed the smallest corolla diameter and the shortest petal length, spur length, and pistil length, followed by EL, while NW and NE showed the largest values of these phenotypes (the difference between NW and NE was not significant) (Supplementary Data Fig. S11A–D). Interestingly, the number of inflorescences showed the reverse order of the above traits (Supplementary Data Fig. S11E). EL showed the largest leaf area, followed by CN, while the difference in leaf area between NW and NE was not significant (Supplementary Data Fig. S11F).

**Figure 5:**
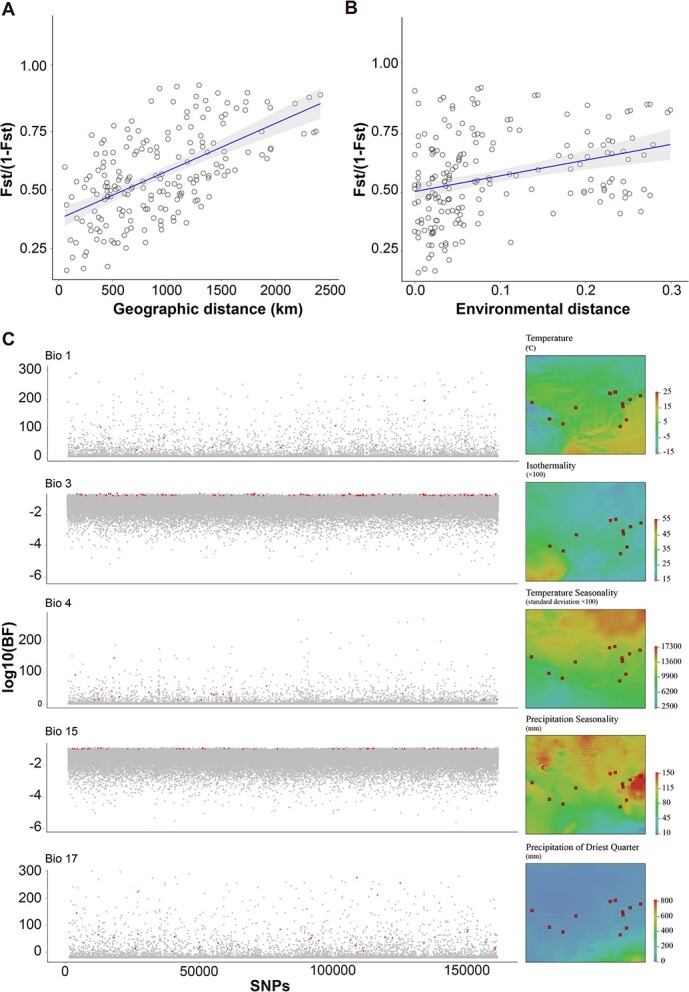
Genomic regions with signals of environmental conditions. (A) Relationship of genetic distance and geographic distance. (B) Relationship of genetic distance and environmental distance. (C) Outlier SNPs associated with bioclimate factors. The red dots indicate that the SNP in the sliding window was selected by the environment; gray dots represent other regions in the genome. The map shows the spatial variation in different bioclimate factors.

Despite being closely related, CN and NW differed in morphology and ecology (Supplementary Data Fig. S10). To explore the genetic mechanism of the differentiation, we identified 1168 outlier SNPs from the 143 318 filtered SNPs according to a 0.05 threshold for the *q*-value between the CN and NW lineages based on the Bayesian method applied in BayeScan (Fig. 4A). The outlier SNPs were in 487 genes that were identified as under positive selection (Supplementary Data Table S10). The *F*_ST_ values of outlier SNPs were significantly higher than those of others, which suggests that the divergence of the lineages from their ancestral population was possibly caused by strong divergent selection. These candidate genes were significantly enriched in ABC-2 type transporter family protein (GO:0016887, *P* = .0151), and we then integrated the candidate genes with organ development, reproductive isolation, and biotic and abiotic stress responses. Among these genes, several (e.g. *PAD2* [73], *DRB3* [74], and *EDM2* [75]) are involved in plant immunity, whereas some are associated with the stresses of drought (e.g. *MAPKKK21* [76] and *SCL1* [77]) and cold (e.g. *CER3* [78] and *LOS1* [79]). Moreover, two genes (*GRF2* [80] and *PHT4;2* [81]) participate in the regulation of leaf size, and several candidate genes are associated with flowering time (e.g. *FT* [82], *FLK* [83], *MYB30* [84], and *FY* [85]), flower organ size (*KNU* [86] and *CKX5* [87]), flower color (*MYB113* [88]), and pollen development (*WLIM1* [89] and *EXO70A2* [90]). The rapid divergence of candidate genes with reproductive function might drive prezygotic isolation across lineages.

### Variation in pollinator assemblage and visitation rate

During the field survey in our study, we recorded 65 and 11 insect pollinators in the YM (representing CN lineage) and HD (representing NW lineage) populations, respectively. The YM population received 15.63% of pollinators from Halicidae (*Lasioglossum villosulum*) and 84.37% of pollinators from Apidae (*Xylocopa appendiculata*, *Eucera floralia*, *Ceratina iwatai*, and *Bombus ignitus*). The HD population received 100% of pollinators from Halicidae (*Lasioglossum morio*) (Fig. 4B, Supplementary Data Table S11). These insect pollinators were identified by DNA barcoding. Although the pollinator species richness of the YM population was higher than that of the HD population, the visitation rate of the HD population was significantly higher than that of the YM population according to Tukey’s pairwise comparisons (*p* = 0.000148), which might have resulted from an environmental difference through their influence on the pollinator assemblages and limitation of the activities of pollinators.

### Candidate genes associated with local climate adaptation

We used a partial Mantel test and MLPE to assess patterns of IBE and IBD. The results of the partial Mantel test revealed that the genetic structure of the *A. viridiflora* complex could be explained by IBD (*r* = 0.392, *p* = 1.20E−03) and IBE (*r* = 0.315, *p* = 0.0041) (Fig. 5A and B), but geographic distance has a greater impact on the genetic environmental difference. MLPE also revealed that, according to the ranking of the AIC and BIC (IBD, AIC = 289.013, BIC = 302.000; IBE, AIC = 344.377, BIC = 357.365), IBD was the optimal model to explain the genetic structure, which is consistent with the inference of the partial Mantel test. Because genetic divergence can result from selection driven by heterogeneous environments via geographic distance, redundancy analysis (RDA) was then implemented to examine the bioclimatic factors related to the 143 318 SNPs. The contribution of seven environmental variables in RDA space is shown in Supplementary Data Fig. S12. The strongest predictor was the mean diurnal range (Bio2), followed by isothermality (Bio3), mean temperature of the wettest quarter (Bio8), temperature seasonality (Bio4), precipitation seasonality (Bio15), annual mean temperatures (Bio1), and precipitation of the driest quarter (Bio17). Bayenv2 identified 179 SNPs significantly associated with the seven examined environmental variables (Fig. 5C). The outlier SNPs were in 83 genes and significantly enriched in PEBP (phosphatidylethanolamine-binding protein) family protein (GO:0003712, two genes, *p* = 0.034), which was previously known to be involved in various physiological processes, such as seasonal growth [91], seed germination [92], and floral transition [93]. In addition, seven genes were adaptively differentiated under temperature and water stress, indicating that the crosstalk between the two modules observed might be important in the process of local adaptation (Supplementary Data Table S12).

## Discussion

Our population genetic analyses of the *A. viridiflora* complex within its main range across northern China suggest a paraphyletic divergence scenario of the species complex with significant phenotypic divergence. These lineages show moderate levels of genetic differentiation and diverged from each other 168 - 239 Kya, a time in the Middle Pleistocene when the most important climate transition occurred, namely, the ‘mid-Brunhes transition’ (MBT, ~400 Kya) [94]. The results further indicate that both geographic isolation and selective pressures via environmental and pollinator assemblage have contributed to the observed lineage differentiation. In the following, these findings will be discussed in more detail.

### Inconsistency between chloroplast and nuclear genomes

Cytonuclear discordance is observed commonly in plant phylogeographic studies, including topological discordance, branch length discordance, and geographic discordance [95]. In our study there were two major clusters in the nuclear genome-wide phylogenetic tree: cluster I corresponds to the *A. viridiflora* complex and cluster II corresponds to other *Aquilegia* species. Among the *A. viridiflora* complex, four lineages (NE, EL, CN, and NW lineages) were identified across the geographic distribution (Fig. 2A). However, the phylogenetic tree based on the chloroplast genome of *Aquilegia* did not show the species boundary and geographic structure in the *A. viridiflora* complex (Fig. 2B). Therefore, two forms of cytonuclear discordance, topological and geographic, existed in *Aquilegia* and the *A. viridiflora* complex, respectively. Both chloroplast capture [96] and incomplete lineage sorting (ILS) [97] are applicable to cytonuclear discordance. Generally, the chloroplast and nuclear genomes of angiosperms are inherited maternally and parentally, respectively. As a classic example of adaptive radiation, interspecific hybridization could be predicted to exist in the genus *Aquilegia* [98]. Chloroplast capture occurs after hybridization and subsequent backcrossing, and the genomes of chloroplasts can be laterally transmitted between sister species [96]. The absence of detectable interspecific gene flow in the nuclear genome revealed in this study also confirmed that chloroplast capture occurred in the *Aquilegia*. On this account, the chloroplast genome is not suitable for inferring the phylogeny of a taxon with radiation speciation, and the difference in sampling location may lead to biased inference. Additionally, there are large effective population sizes (ancestral polymorphism) in the *A. viridiflora* complex (Fig. 3C), and the chloroplast phylogeny was not geographically biased and random, which also supports the idea that ILS may play a role in cytonuclear discordance in the *A. viridiflora* complex, and the random process often occurs in the formation of taxons by rapid adaptive radiation.

### Lineage divergence across geographic distribution

Although our study did not include samples from the Far East of Russia, it covered the primary distribution area of the *A. viridiflora* complex. Erst *et al*. [30] reported that there are no obvious morphological differences between the *A. viridiflora* complex (*A. kamelinii*) in the Far East of Russia and those from the WD population (Supplementary Data Fig. S1). Additionally, no other genetic components were detected in the WD population (Fig. 1B). Similar sampling strategies were employed for other taxa that are widely distributed in the north temperate zone to infer population genetic structure, such as *Populus euphratica* [99], *P. davidiana* [27, 100], and *Juglans mandshurica* [101]. Therefore, while the inference of phylogenetic relationships may have been impacted by incomplete sampling due to a lack of *A. viridiflora* complex samples from the Far East of Russia, our samples are still suitable for exploring the population genome genetic variation and dynamic history of the *A. viridiflora* complex.

Using nuclear SNPs from available samples, four lineages were identified with phylogenetic, Bayesian clustering approaches, and PCA based on nuclear SNPs: the NE lineage included *A. viridiflora* and *A. kamelinii*, the EL lineage included *A. viridiflora* and *A. hebeica*, and the CN and NW lineages only included *A. hebeica* and *A. viridiflora*, respectively, which differs from taxonomically recognized species (Fig. 1A and B). Both the partial Mantel test and the MLEP model showed that geographic distance could explain the variance in genetic differentiation (Fig. 4B), but considering the continuous distribution of the *A. viridiflora* complex, a strong pattern of IBD was observed across all of the study populations as expected, so the genetic differentiation of the *A. viridiflora* complex cannot be fully explained by geographic isolation. The moderate genetic divergence (both *F*_ST_ and *D*_XY_) among different lineages may be explained by three non-mutually exclusive factors, IBD, IBE, and gene flow. We took advantage of the whole-genome dataset of the *A. viridiflora* complex to infer the amount of gene flow of the recovered lineages, revealing that a higher level of recent gene flow than in ancient times was shown to have occurred. Gene flow between pairs of lineages with close geographic distribution ranges was relatively large, indicating that geographic isolation might shape the present range and phylogeny of the recovered lineages. Hybrids with two or three lineages mixed in the contact zone existed because of the absence of strong biogeographic barriers; at the same time, gene flow among populations and after lineage differentiation may decrease the level of genetic differentiation, resulting in the discordance between genetics and morphology in some populations (Figs 1A and 2A). The MS and TS populations in the Liaodong Peninsula were morphologically diagnosed as *A. hebeica* but are genetically closer to *A. viridiflora* than *A. hebeica*.

That the *A. viridiflora* complex was already present in Asia by 1.19 Mya is evident from the age model of Fior *et al*. in accordance with molecular clock estimates of the flora of Altai (*A. glandulosa*, *A. sibirica*, and *A. viridiflora*) [33]. It is clear that the present lineages must have diverged much more recently through our demographic history simulations. The model indicates a very young divergence time in the *A. viridiflora* complex: ~239, ~211, and ~168 Kya (Fig. 3C). Although these ages must be explained with caution, the most important climate transition occurred in the Middle Pleistocene, which might have resulted in lineage divergence by changing the suitable habitats of species. The role of Middle Pleistocene climate transitions in speciation processes has long been considered, such as the *Ranunculus auricomus* complex [102], *Populus rotundifolia* [103], and *Cerapanorpa brevicornis* [104]. Here, we add to these ‘species on the speciation way’ examples in recent Middle Pleistocene speciation and indicate that the very high rates of speciation associated with *Aquilegia* adaptive radiation might be driven by the Middle Pleistocene climate transition. Additionally, a bottleneck event was inferred in the four lineages of *A. viridiflora* complex at the LGM and enhanced genetic drift, which indicated the role of climate changes in the demographic history and allele frequency [105]. The partial Mantel test and the MLEP model also revealed that environmental differences may be an explanation in the *A. viridiflora* complex. Heterogeneous environments and different environmental variables exert differential selection pressure on the different lineages. We found 83 genes related to environmental factors that may play a key role in the continuously adaptive process (Fig. 4C). Some genes associated with the abscisic acid (ABA) signaling pathway (*AIN1* [106] and *AAO4* [107]) can regulate numerous ABA responses and may induce abiotic stress responses for defense in different environments. Therefore, the incipient lineage divergence of the *A. viridiflora* complex could be largely explained by geographic isolation, environmental difference, and the decrease in gene flow. These results are in line with recent studies suggesting that gene flow may have occurred between currently geographically and environmentally isolated East Asia and played a major role in other plant radiations [108–110].

### Local selection and adaptation

Apart from the genetic divergence revealed by phylogenetic and population structure analyses, clear differentiation in phenotypic traits was also exhibited based on common garden data (Fig. 1A). Divergences in corolla diameter, petal length, spur length, pistil length, inflorescence number, and leaf area were probably driven by selection. Among these traits, floral characteristic differentiation might act as a prezygotic isolation mechanism between divergent lineages. We recovered four lineages based on the nuclear genome, two of which are closely related (CN lineage diagnosed as *A. hebeica* and NW lineage diagnosed as *A. viridiflora*) and correspond to distinct floral characteristics (Fig. 1A, Supplementary Data Fig. S4). Changes in the pollinator assemblage impact floral evolution and reproductive success. Pollinator assemblages and visitation rate were significantly different in two lineages (Fig. 4B), and therefore strong natural selection produced obvious phenotype differences between two close lineages, i.e. the NW lineage has larger flowers to attract pollinators, whereas the CN lineage has a greater number of flowers to gain longer visitation rates of pollinators. However, the distantly related NE and NW lineages shared similar phenotypes, as did the MS and TS populations in the EL and CN lineages, which suggests that allopatric lineages adapt to similar habitats without phenotype divergence. In summary, both divergent ecological pressure and similar ecological pressures promoted the divergence of allopatric lineages in the *A. viridiflora* complex during the early stage of speciation.

Additionally, we found 487 genes under positive selection and exhibiting high divergence between lineages (Fig. 4A). These genes were significantly enriched in the ABC-2 type transport family protein, a gene family involved in a wide range of metabolism in plants and playing important roles in seed germination, stomatal movement, lateral root development, and responses to various environmental stresses [111–113]. Among these selected genes, *KNU* [86] and *CKX5* [87] are involved in the regulation of flower morphology and development, for which a major difference exists between the CN and NW lineages. Similarly, *GRF2* [80] and *PHT4;2* [81] in these lineages has been initiated due to their relation to leaf development and morphology. *NRPD1B* is related to panicle branches [114], and high allelic divergence and fixation of this gene in CN lineages may contribute to producing more inflorescence relative to NW lineages. Moreover, several genes (e.g. *PAD2* [73], *DRB3* [74], and *EDM2* [75]) are involved in disease resistance in response to biotic stresses. Plants can adjust growth and defense based on different environments to survive and reproduce in the natural world, which might contribute to lineage divergence [115]. Therefore, our study showed that geographic isolation, environmental isolation, and natural selection drove the lineage divergence of the *A. viridiflora* complex and created geographic distributions of phenotypic variations. Further work is needed to acquire more accurate knowledge of the functions of genes in the whole genome under selective pressure and clarify potential adaptation patterns.

### Conclusions

In summary, we explored the phenotypic and genetic diversification of 20 populations of the *A. viridiflora* complex across its range. Our results revealed inconsistencies of lineage and phenotypic divergence in the *A. viridiflora* complex, which may be caused by continuous gene flow between lineages during lineage divergence. Furthermore, geographic isolation and divergent selection due to heterogeneous environments and pollinator assemblages led to the lineage divergence of the *A. viridiflora* complex. However, allopatric lineages with similar habitats did not show phenotypic differentiation involving similar pressures, indicating that allopatric speciation could occur with or without divergent adaptation. More importantly, our findings provide genomic insights into the integration of information on gene flow, divergence time of lineages, population effect size, and divergent selection to identify genes potentially involved in speciation and the basis of geographic distributions of phenotypic variations. This study may also accelerate further taxonomic and evolutionary studies on the *A. viridiflora* complex and other species in East Asia.

## Materials and methods

### Study system

The *A. viridiflora* complex (Ranunculaceae), including *A. viridiflora*, *A. hebeica*, and *A. kamelinii*, consists of perennial herbaceous plant throughout the mid-latitudes of the Northern Hemisphere. Seeds of the *A. viridiflora* complex were collected from 20 locations covering its current distribution range; each of the maternal plants was separated from the others by > 50 m. Moreover, to explore whether the *A. viridiflora* complex collected shared the most recent common ancestor (MRCA) and the existence of interspecies gene flow, we also collected other congener species whose distribution overlapped with that of the *A. viridiflora* complex, including *A. amurensis* Kom., *A. ecalcarata* Maxim., *A. japonica* Nakai et Hara, *A. oxysepala* Trautv. et Mey. var. *kansuensis* Bruhl, *A. oxysepala* Trautv. et Mey., and *A. yabeana* Kitag. seeds, as reported in our previous study [35]. Additionally, *Paraquilegia microphylla* (Royle) Drumm. et Hutch. was collected as an outgroup. All voucher specimens were identified by Dr Hongxing Xiao and deposited in the Northeast Normal University Herbaria (Fig. 1A; Supplementary Data Table S1).

### Common garden phenotypic measurements and statistics

Five to ten seeds of each individual were sterilized and planted in pots under 12:12 hours light:dark conditions 25°C/20°C for 3 months. Then, we transplanted two plants of each female parent to outdoor agricultural fields at Northeast Normal University. For phenotypic measurement, seven regenerative traits and four vegetative traits of different populations in the experimental field during the full flowering stage were measured. To ensure the accuracy of the measurements, we randomly selected three to five plants from each population to measure the number of inflorescences and plant height; among these plants, six flowers and six leaves were randomly selected from each plant, and the corolla diameter, pistil length, stamen length, leaf area, leaf perimeter, and chlorophyll content were measured and recorded. Among these six flowers, we randomly selected three petals and calyxes to measure petal length (not including spurs), calyx length, spur length, and calyx length.

To reduce the error caused by different measurement batches, we used a mixed linear model to evaluate traits in the lme4 [36] package in R according to the following regression model equation:

$$ {Y}_i=\mathrm{\mu} +{\mathrm{\beta}}_1A+{\mathrm{\beta}}_2P+{\mathrm{\varepsilon}}_i $$

where ${Y}_i$ represents the traits of different populations, $\mathrm{\mu}$ is the actual measurement, ${\mathrm{\beta}}_1$ and ${\beta}_2$ are regression coefficients, *A* represents the measurement batches, *P* is the person conducting the measurement, and ${\mathrm{\varepsilon}}_i$ is the residual variance. The evaluation results were used for ANOVA and *K*-means cluster analysis in R.

### DNA sequencing and single-nucleotide polymorphism calling

Fresh leaves were collected from 1 population of *A. kamelinii*, 6 populations of *A. hebeica*, and 13 populations of *A. viridiflora* to extract genomic DNA using a modified cetyltrimethylammonium bromide (CTAB) method [37], bringing the total number of sequence samples to 66 (Supplementary Data Table S1). In addition, 12 individuals from other *Aquilegia* species overlapped the distribution of the *A. viridiflora* complex, and 1 individual of *P. microphylla* was also used to extract genomic DNA. For each individual, 10 μg genomic DNA was randomly fragmented and the fragments of size 500 bp were recovered following the manufacturer’s protocol to construct the sequencing library. Sequencing was performed on the Illumina X Ten platform from Biomarker Technologies, Inc. (Beijing, China) with PE150 according to the manufacturer’s protocol. Furthermore, the sequence reads of *Semiaquilegia adoxoides* (SRR437677) were downloaded from the NCBI SRA database (http://www.ncbi.nlm.nih.gov/sra) to be used as an outgroup. To obtain high-quality single-nucleotide polymorphisms (SNPs), all the reads were subjected to quality control by FastQC [38] and filtered as follows: reads with adapters and reads with > 10% N content or > 50% low-quality bases (quality value < 10) were removed. Low-quality reads were removed using NGStoolkit [39].

High-quality clean reads of 80 individuals were mapped to the reference genome of *A. coerulea* from the previous study of Filiault *et al*. [40] using BWA v.0.7.12 with default parameters [41]. As the *Aquilegia* species are closely related [32], the same reference genome could be used to call SNPs in all the *Aquilegia* species with high accuracy in our study [40, 42]. SAMtools v.0.1.18 was used to convert SAM files to BAM files and sort reads [43]. The HaplotypeCaller, GenotypeGVCFs, and CombineGVCFs modules in GATK v.4.1.8.0 were used to produce accurate SNP calls [44]. To improve the quality of SNPs, the VariantFiltration module in GATK v.4.1.8.0 was used for filtration with the following parameters: --filter-name FilterQual --filter-expression QUAL < 30.0 --filter-name FilterQD --filter-expression QD < 2.0 --filter-name FilterMQ --filter-expression MQ < 40.0 --filter-name FilterFS --filter-expression FS > 60.0 -window 5 -cluster 2. Next, VCFtools v.0.1.13 [45] was used to remove variants that (i) showed a minor allele frequency (MAF) of ≤ 0.05, (ii) were not biallelic variants, (iii) showed a sequencing depth of < 5, and (iv) showed a missing rate > 0.5. After aligning the reads to the reference genome and filtering, we obtained 1,064,089 high-quality biallelic SNPs for downstream analysis (Dataset 1). Moreover, to identify the loci with the ancestral state of the *A. viridiflora* complex, SNPs in Dataset 1 were detected according to the method described by Ma *et al*. [9]. After the removal of other *Aquilegia* species, we identified 496 715 SNPs with the ancestral state.

### Population genetic structure and gene flow analysis of *Aquilegia* species

We estimated the phylogenetic relationships of other *Aquilegia* species with the *A. viridiflora* complex using IQ-TREE multicore version 1.6.12 [46] with 1000 bootstrap replicates to determine whether the *A. viridiflora* complex in our study shared an MRCA. The maximum likelihood (ML) tree indicated that 20 populations of the *A. viridiflora* complex shared an MRCA. All trees were illustrated in iTOL (http://itol.embl.de). Then we used PLINK to remove linkage sites from Dataset 1, with the parameter indep-pairwise 50 10 0.2 and obtained a total of 243 657 independent SNPs (Dataset 2). The independent SNPs were filtered by excluding sites located within 1 kb of gene regions and obtained 77 520 SNPs. ADMIXTURE v.1.3.0 [47] was applied to investigate the ML of the ancestry of all *Aquilegia* individuals based on independent and neutral loci with *K* values ranging from 2 to 10 with 10 replicates for each *K* value, and examined the optimum *K* value according to the lowest value of the error rate.

To test for gene flow between the *A. viridiflora* complex and other *Aquilegia* species across contact zones, we applied the ABBA-BABA or *D* statistic using the qpDstat module in AdmixTools 7.0 [48] based on the allele frequency of SNP Dataset 1. Following the topology {[(P1, P2), P3], O}, where P1 and P2 are two groups of the *A. viridiflora* complex and P3 is other *Aquilegia* species according to the phylogenetic relationships recovered above, with *P. microphylla* and *S. adoxoides* as outgroups, significant deviation of the *D* statistics from zero suggests that P3 exchanged genes with P1 or P2 [49]. An absolute *Z*-score with a value >3 was considered statistically significant. In addition to the *D* statistic, we performed gene flow analysis between different populations using allele frequency data with 1 - 10 migration events by TreeMix v.1.1 [50] based on SNP Dataset 2 with five iterations of each, using the -se and -global options to calculate the standard errors of the migration weights. To determine the best number of migration edges, OPTM v.0.1.5 [51] packages in R were used through the output files of TreeMix.

### Phylogeny of *Aquilegia* species chloroplast genomes

To avoid the effect of sequence redundancy when inferring the phylogeny of *Aquilegia* species, we selected the LSC (large single-copy) regions, IRB (inverted repeat B) regions, and SSC (short single-copy) regions of the *A. viridiflora* chloroplast genome (MN809220) as a reference, clean reads were aligned to the reference to obtain the variation of *Aquilegia* species in the chloroplast genome and SNPs were called according to the above pipeline. Therefore, 257 high-quality SNPs in the chloroplast genome were used for constructing the ML tree with IQ-TREE multicore version 1.6.12 [46] and illustrated in iTOL (http://itol.embl.de). The chloroplast genomes of *P. microphylla* and *S. adoxoides* were also regarded as outgroups [52].

### Population genetic structure of *A. viridiflora* complex

To infer the lineage divergence and explore the role of gene flow in the speciation of the *A. viridiflora* complex,  672 439 high­quality SNPs for downstream analysis were obtained from the remaining populations of the *A. viridiflora* complex after removing other *Aquilegia *species. We used a phylogenetic network by the NeighborNet algorithm in the software SplitsTree [52] with 1000 bootstrap replicates to infer and visualize patterns of potential reticulate evolution. Moreover, principal component analysis (PCA) was performed using EIGENSOFT v.6.1.4 [53] to infer population genetic structure. By combining the results of the phylogenetic relationship and genetic structure analyses to establish lineages for downstream analysis, we divided 20 populations into four lineages. Since the results of the population genetic structure showed a mixed genetic background in some individuals, the Python package HyDe was used to identify hybridization events at the individual level [54]. Among these individuals, P2 did not show a mixed genetic background, and P1 and P3 were individuals of other populations.after removing other *Aquilegia* species, 672 439 high­quality SNPs for downstream analysis were obtained from the remaining populations of the *A. viridiflora* complex.

In addition, SNPs in the chloroplast genome were also used to infer the phylogeny of the *A. viridiflora* complex using SplitsTree. The haplotype network of chloroplast genome analysis was performed using the median-joining network in PopART v.1.7 with default parameters [55].

### Demographic history inference of *A. viridiflora* complex

Hybrid individuals were removed due to the levels of heterozygosity, and the remaining individuals were used for subsequent analysis. The gene flow between each lineage in the *A. viridiflora* complex was inferred by applying the ABBA-BABA test and Treemix v.1.1 based on the same method as above. The Python script easySFS was used to calculate the joint site frequency spectrum (SFS) for demographic analysis (https://github.com/isaacovercast/easySFS). We also calculated the likelihood of different demographic scenarios in fastsimcoal2 software [56] using the joint SFS to infer demographic parameters. Twenty scenarios were set up involving genetic structure and gene flow, including 3 monophyletic models and 16 paraphyletic models (Supplementary Data Fig. S8). For each scenario, fastsimcoal2 performed 10 000 coalescent simulations to approximate the expected SFS in each cycle and 40 optimization cycles were run to estimate the parameters. To ensure the accuracy of evaluating the best scenario, each scenario was run 100 times, and the run with the highest likelihood was compared by calculating the Akaike information criterion (AIC) to determine the best scenario. The parameter estimation was run under the best scenario 100 times with each of the bootstrapped SFSs. SMC++ v.1.15.3 [57] was used to infer the demographic trajectories of divergent lineages of the *A. viridiflora* complex. A neutral mutation rate of 10^−8^ per site per generation and a generation time of 1 year were used to estimate the effective population size, divergence times, and migration rates [34].

### Genetic diversity and linkage disequilibrium decay

Nucleotide diversity (π) was calculated for the four groups using a 100-kb non-overlapping sliding window by using VCFtools [45]. Additionally, the fixation index (*F*_ST_) and nucleotide divergence (*D*_XY_) between each of the four groups were calculated by PIXY [45] in 100-kb sliding windows. PopLDdecay [58] software was applied to compute linkage disequilibrium (LD) decay among different groups and chromosomes separately.

### Adaptation analysis

The divergence in quantitative traits (Q_ST_) and F_ST_ were be compared to explore the effect of adaptation on 11 quantitative traits, so we used the single-phenotype *Q*_ST_–*F*_ST_ test with the R package QstFstComp [59]. If *Q*_ST_ > *F*_ST_, the differentiation of traits is the major effect of divergent selection and shows local adaptation. We used the half-sib dam model and 10 000 resampling steps for each *Q*_ST_–*F*_ST_ analysis. Taking into account the phenotypic and genetic variation of the *A. viridiflora* complex, we compared the levels of *F*_ST_ between the CN and NW lineages to identify candidate loci under natural selection from 143 318 SNPs by BayeScan v.2.1 [60] software with default parameters, and PGDSpider [61] was used to produce an input file for BayeScan. SNPs with a *q* value <.05 were considered potentially selected loci. Candidate SNPs were mapped to the corresponding genes, and Gene Ontology (GO) functional enrichment analysis was performed by the clusterProfiler package [62] in R to calculate the *P* values of Fisher’s exact test and correction for multiple testing was performed using the Benjamini–Hochberg (BH) method based on the *A. coerulea* gene annotation information. GO terms with a BH correction false discovery rate (FDR) value < 0.05 were considered to be significantly enriched.

### Testing isolation by distance and isolation by environment

Temperature and precipitation are considered the driving factors that affect the population growth rate and limit the distribution of species [63–65]. Therefore, 19 current bioclimatic variables with a spatial resolution of 30 arcseconds were collected from WorldClim (http://www.worldclim.org/). Simultaneously, we recorded the GPS information of the sampling locations and downloaded the GPS information for *A. viridiflora* from the Chinese Virtual Herbarium (CVH; http://www.cvh.ac.cn) and the Global Biodiversity Information Facility (GBIF; http://www.gbif.org). ArcMap v.10.4 was used to limit the spatial extent according to the buffer radius (5 km) around each occurrence record. We used |*r*| < .8 (Pearson correlation coefficient) as a cutoff to remove highly correlated variables. The seven retained current bioclimatic factors (Bio1, annual mean temperature; Bio2, mean diurnal range; Bio3, isothermality; Bio4, temperature seasonality; Bio8, mean temperature of the wettest quarter; Bio15, precipitation seasonality; Bio17, precipitation of the driest quarter) were used for subsequent analysis.

Isolation by environment (IBE) and isolation by distance (IBD) should take the spatial dependence into account in the data. We therefore ran a partial Mantel test using 999 permutations with significance determined to explore the effects of environmental differences and geographic distance on genetic distance in the R package vegan [66]. Environmental distance and geographic distance were represented by Euclidean distances. Genetic distances among populations were represented by *F*_ST_/(1 − *F*_ST_) [67]. The genetic distance was used as the response, the environment distance as the predictor, and the geographic distance as the condition factor to test IBE. On the contrary, the roles of environmental distance and geographic distance were exchanged to test IBD. Moreover, we analyzed the impact of fixed factors (bioclimatic and geographic) on the random effect of the population using a maximum likelihood population effect model (MLPE) in R package corMLPE [68] to avoid the error caused by the partial Mantel test [69, 70]. As the objective criteria, the AIC and Bayesian information criterion (BIC) were used to evaluate the model fitting of different models.

### Environmental associations of candidate loci

Redundancy analysis (RDA) was used to assess the impact of current bioclimatic factors on the genomic composition of the *A. viridiflora* complex in R. For the same reason as selection analysis, we also used the CN and NW lineages to identify the loci related to the seven retained current bioclimatic factors. Bayenv2 [71] was used with 1 000 000 iterations and run three times separately. For each bioclimatic factor, the SNPs among the top 1% according to Bayes factor (BF) and among the top 5% according to the absolute Spearman’s ρ were considered candidates. For genes with candidate SNPs we performed GO functional enrichment analysis according to the same methods as above.

### Pollinator insect assemblages in natural populations

To compare the divergence of pollinator insects of the CN and NW lineages, we selected the YM and HD populations for field observations of insects. During April and May of 2021, which is the peak flowering time for *A. viridiflora* complex in these regions, we sampled and recorded pollinator insects with haphazard selection of seven patches (ranging from ~20 to 30 flowers in each patch) at different days and times of day. On the first day of field observation, pollinator insects of the *A. viridiflora* complex visited flowers very rarely after 5 p.m. Therefore, we decided to restrict field observation to 8 a.m. to 5 p.m. for each study day and the field observations lasted for at least 3 full days with good climate conditions. We recorded the pollinator insect species, the duration of visitation, and the number of flowers visited by the pollinator. Then, the pollinator visitation rates were calculated as the duration of visitation dividing the number of flowers visited by the pollinator. We used clean entomological net in different sampling sites to prevent cross-pollution between samples from each site. After collection, each insect was stored separately in a tidy tube that was filled with 75% ethanol.

We used DNA barcoding basing on mitochondrial cytochrome oxidase subunit I (COI) to identify tentatively the pollinator insects. The thorax or leg of insects was used for DNA extraction. The primer pair LCO1490 (5′-TTGATTTTTTGGTCA TCCAGAAGT-3′) – HCO2198 (5′-TCCАATGСAСТAATСTGCСAТATTA-3′) was used to amplify DNA fragments. The protocols of laboratory and data processes were adopted as described in Galimberti *et al*. [72]. Nucleotide sequences newly obtained in this study have been deposited in NCBI under accession numbers QQ168183–QQ168189.

## Acknowledgements

We are grateful to Jianquan Liu for suggestions and comments to improve the manuscript; we also thank reviewers for their comments on the manuscript; Mingzhou Sun and Shu Wang for collecting the plant materials; and Peng Peng for providing photographs. The research was supported by the National Natural Science Foundation of China (32070244) and Fundamental Research Funds for the Central Universities.

## Author contributions

X.H.X. and W.H.Y. designed the study and evaluated the results; W.H.Y. and Z.W. collected the materials; Z.W., Z.T.J., F.X.X., and L.M.Y participated in data analysis; Z.W. and W.H.Y. prepared the manuscript; all authors read and approved the final manuscript.

## Data availability

Raw sequence data are available from the National Center for Biotechnology Information’s (NCBI) Sequence Read Archive (SRA) under the submission PRJNA666554. Data are available from the Dryad Digital Repository (https://doi.org/10.5061/dryad.cnp5hqc8g).

## Supplementary data

Supplementary data is available at *Horticulture Research* online.

## Supplementary Material

Web_Material_uhad041
