# Supplementary material for: Geographic–genomic and geographic–phenotypic differentiation of the Aquilegia viridiflora complex
Source: Hortic Res. 2023 Mar 13;10(5):uhad041. doi: 10.1093/hr/uhad041 (PMC10163360; doi:10.1093/hr/uhad041)
Supplement: Web_Material_uhad041 [file web_material_uhad041.zip › Supplemental information_clean.docx]

**Supplemental information**

**Figure S1** Phenotypes of the different populations of *Aquilegia viridiflora* complex in the common garden.

**Figure S2** Correlation analysis between phenotypes. * represents a significant relationship between different phenotypes, 0.01 < p <0.05. ** represents a significant relationship between different phenotypes, 0.001 < p <0.01. *** represents a significant relationship between different phenotypes, p < 0.001.

**Figure S3** Cross-validation results corresponding to different K values in the ADMIXTURE analysis of *Aquilegia viridiflora* complex.

**Figure S4** Optimal migration edges of TreeMix analysis.

**Figure S5** NeighborNet diagram of *Aquilegia viridiflora* complex based on genome.

**Figure S6** A. NeighborNet diagram based on chloroplast genome, B. Median-joining haplotype network of *Aquilegia viridiflora* complex based on chloroplast genome.

Figure S7 Five gene flow events of *Aquilegia viridiflora* complex. Each branch represents a population, and arrows indicate migration events that occur between populations.

**Figure S8** Four groups of possible models for inferring demographic parameters. The gray arrows represent the current gene flow, the red arrows represent the ancient gene flow, and the model in the dotted line is the best model selected according to the values of the likelihoods and AIC.

**Figure S9** Comparison of observed data and expected data based on the best model for A. NE and EL, B. NE and CN, C. EL and CN, D. NE and NW, E. EL and NW, and F. CN and NW.

Figure S10 A. Nucleotide diversity (θ_π_) of *Aquilegia viridiflora* complex, B. pairwise F_ST_ and D_XY_ between different groups, C. LD decay of the four lineages of *A. viridiflora* complex. Except that the difference between the two groups marked in the figure is not significant, there is a significant difference between any other two groups.

Figure S11 Phenotype of *Aquilegia viridiflora* complex. A. Corolla diameter, B petal length (not including spur), C. spur length, D. pistil length, E Number of inflorescences, F Leaf area. Except that the difference between the two groups marked in the figure is not significant, there is a significant difference between any other two groups.

**Figure S12** RDA plot of *Aquilegia viridiflora* complex showing SNPs with are potentially associated with seven environmental factors.

**Table S1** Sampling information and the value of seven environmental factors.

**Table S2** Summary and mapping statistics of the re-sequencing.

**Table S3.** Results of the D-statistics in *Aquilegia.*

**Table S4.** Results of the HyDe.

**Table S5.** Polymorphisms in the chloroplast genome of *A. viridiflora* complex.

**Table S6.** Haplotype in the chloroplast genome of *A. viridiflora* complex.

**Table S7.** Results of the D-statistics.

**Table S8.** The AIC value of different models shown in Figure S7.

**Table S9.** Inferred parameters estimates with 95% confidence intervals for the best-fitting demographic scenario modelled in fastasimcoal2.

**Table S10.** The gene list under the selection.

**Table S11.** Pollinator insects assemblages and visitation frequency at different locations.

**Table S12.** The gene list correlation with the environment.

**Table S1** Sampling information and the value of seven environmental factors.

| **Population** | **Latitude (N)** | **Longitude (E)** | **Bio1** | **Bio2** | **Bio3** | **Bio4** | **Bio8** | **Bio15** | **Bio17** | **Number** |
| --- | --- | --- | --- | --- | --- | --- | --- | --- | --- | --- |
| WD | 48.046 | 126.076 | 15 | 120 | 21 | 15295 | 199 | 111 | 10 | 3, 5 |
| AE | 47.410 | 120.562 | -32 | 132 | 25 | 14276 | 141 | 103 | 17 | 5, 5 |
| SZ | 43.932 | 117.523 | -12 | 129 | 26 | 12931 | 147 | 106 | 11 | 5, 3 |
| LF | 43.818 | 127.411 | 28 | 130 | 25 | 13770 | 193 | 98 | 21 | 5, 3 |
| LT | 38.741 | 121.162 | 101 | 79 | 22 | 9815 | 218 | 101 | 24 | 4, 3 |
| YS | 37.219 | 121.066 | 94 | 75 | 22 | 9426 | 207 | 96 | 35 | 4, 3 |
| MS | 35.557 | 117.969 | 121 | 104 | 28 | 9771 | 242 | 101 | 36 | 4, 4 |
| TS | 36.257 | 117.084 | 83 | 83 | 24 | 9498 | 200 | 104 | 38 | 4, 4 |
| YT | 35.467 | 113.339 | 127 | 120 | 30 | 9721 | 231 | 89 | 23 | 5, 4 |
| QB | 35.558 | 117.963 | 117 | 102 | 27 | 9718 | 237 | 101 | 36 | 5, 3 |
| TL | 38.74 | 113.812 | 28 | 119 | 29 | 10286 | 154 | 101 | 19 | 4, 3 |
| YM | 40.556 | 116.701 | 61 | 124 | 28 | 11334 | 199 | 117 | 11 | 4, 3 |
| HL | 39.27 | 113.731 | 61 | 130 | 29 | 10909 | 194 | 101 | 15 | 5, 3 |
| XW | 39.944 | 114.966 | 27 | 120 | 28 | 11317 | 164 | 95 | 17 | 5, 3 |
| HH | 41.144 | 112.543 | 6 | 124 | 27 | 11599 | 148 | 103 | 11 | 5, 3 |
| HD | 40.954 | 111.672 | 31 | 125 | 27 | 11995 | 177 | 110 | 9 | 5, 2 |
| YC | 38.609 | 106.032 | 89 | 133 | 29 | 11055 | 207 | 96 | 4 | 5, 3 |
| SF | 35.927 | 103.884 | 51 | 119 | 31 | 8622 | 146 | 87 | 9 | 5, 3 |
| ZG | 36.715 | 101.655 | 55 | 138 | 35 | 8471 | 148 | 92 | 5 | 4, 3 |
| JQ | 39.458 | 98.803 | 69 | 139 | 31 | 10573 | 196 | 84 | 5 | 4, 3 |

The front number is the number of phenotypic measurements, the behind number is the number of sequencing.

**Table S2 Summary and mapping statistics of the re-sequencing.**

| **Individual** | **Clean Reads** | **Clean Base (bp)** | **Q20(%)** | **GC Content (%)** | **Depth** | **Mapping Ratio (%)** |
| --- | --- | --- | --- | --- | --- | --- |
| LF-1 | 27,884,459 | 4,123,104,300 | 97.04 | 39.16 | 13.50 | 95.70% |
| LF-2 | 28,125,306 | 4,153,267,200 | 97.08 | 38.81 | 13.60 | 97.18% |
| LF-3 | 32,337,592 | 4,777,469,400 | 97.31 | 38.53 | 15.64 | 97.76% |
| WD-2 | 17,590,508 | 5,268,389,426 | 97.84 | 37.72 | 17.25 | 97.08% |
| WD-6 | 18,703,277 | 5,602,043,394 | 97.64 | 38.07 | 18.34 | 98.21% |
| WD-8 | 16,072,157 | 4,813,815,612 | 97.99 | 37.81 | 15.76 | 97.04% |
| WD-10 | 18,106,366 | 5,423,466,426 | 98.10 | 37.62 | 17.76 | 96.99% |
| WD-12 | 18,061,632 | 5,409,826,144 | 98.01 | 37.76 | 17.71 | 96.33% |
| AE-1 | 18,667,027 | 5,588,837,584 | 97.48 | 38.00 | 18.30 | 98.38% |
| AE-2 | 19,394,009 | 5,804,912,734 | 97.38 | 38.17 | 19.00 | 98.95% |
| AE-3 | 19,589,175 | 5,866,346,372 | 97.11 | 38.10 | 19.21 | 98.96% |
| AE-4 | 15,042,922 | 4,505,328,542 | 97.62 | 38.02 | 14.75 | 94.93% |
| AE-5 | 19,846,935 | 5,943,406,800 | 97.53 | 38.39 | 19.46 | 98.55% |
| SZ-1 | 13,792,879 | 4,131,350,386 | 97.97 | 37.75 | 13.53 | 98.55% |
| SZ-3 | 14,320,832 | 4,286,872,442 | 97.95 | 37.49 | 14.03 | 98.52% |
| SZ-6 | 14,251,963 | 4,267,341,108 | 96.85 | 38.15 | 13.97 | 98.42% |
| LT-1 | 19,336,568 | 5,789,550,852 | 97.42 | 38.07 | 18.95 | 98.72% |
| LT-4 | 19,063,380 | 5,706,506,434 | 97.37 | 38.22 | 18.68 | 98.70% |
| LT-5 | 16,591,276 | 4,969,541,970 | 97.49 | 38.29 | 16.27 | 96.07% |
| YS-2 | 18,678,535 | 5,594,268,942 | 96.87 | 38.09 | 18.31 | 97.46% |
| YS-4 | 18,971,254 | 5,679,482,792 | 97.35 | 38.02 | 18.59 | 97.03% |
| YS-5 | 16,650,110 | 4,986,987,594 | 97.87 | 37.97 | 16.33 | 96.55% |
| MS-3 | 20,926,444 | 6,267,764,968 | 98.07 | 37.90 | 20.52 | 98.42% |
| MS-4 | 16,158,193 | 4,838,069,618 | 98.16 | 38.44 | 15.84 | 98.65% |
| MS-5 | 17,394,498 | 5,209,792,442 | 97.67 | 38.39 | 17.06 | 95.76% |
| MS-6 | 19,565,964 | 5,860,103,978 | 97.80 | 38.20 | 19.19 | 96.03% |
| TS-2 | 18,926,237 | 5,668,119,350 | 96.79 | 37.77 | 18.56 | 98.32% |
| TS-3 | 18,794,846 | 5,628,589,684 | 97.86 | 38.40 | 18.43 | 96.16% |
| TS-4 | 15,025,407 | 4,500,616,314 | 97.89 | 38.23 | 14.73 | 98.30% |
| TS-6 | 15,041,214 | 4,504,857,326 | 97.65 | 38.24 | 14.75 | 97.81% |
| YT-1 | 19,412,446 | 5,812,360,226 | 97.46 | 38.02 | 19.03 | 98.63% |
| YT-9 | 17,255,020 | 5,168,771,130 | 97.71 | 38.07 | 16.92 | 96.67% |
| YT-8 | 17,267,428 | 5,172,014,476 | 96.63 | 38.39 | 16.93 | 95.40% |
| YT-10 | 18,931,211 | 5,669,429,628 | 97.09 | 37.62 | 18.56 | 96.35% |
| QB-5 | 19,361,397 | 5,798,803,118 | 96.88 | 37.68 | 18.98 | 98.48% |
| QB-8 | 17,880,192 | 5,355,126,922 | 97.59 | 38.32 | 17.53 | 98.19% |
| QB-9 | 16,946,408 | 5,075,156,830 | 97.81 | 38.62 | 16.62 | 96.97% |
| TL-3 | 18,776,362 | 5,623,780,642 | 97.72 | 38.17 | 18.41 | 97.52% |
| TL-8 | 19,583,855 | 5,865,769,408 | 97.25 | 38.19 | 19.20 | 98.63% |
| TL-12 | 17,553,686 | 5,257,410,500 | 97.49 | 38.36 | 17.21 | 97.38% |
| YM-1 | 19,432,934 | 5,820,256,126 | 97.00 | 37.99 | 19.05 | 98.56% |
| YM-4 | 18,160,633 | 5,438,827,874 | 97.66 | 38.33 | 17.81 | 96.11% |
| YM-5 | 17,356,613 | 5,198,268,554 | 97.68 | 38.14 | 17.02 | 98.25% |
| XW-2 | 15,824,498 | 4,739,149,170 | 97.93 | 37.66 | 15.52 | 98.02% |
| XW-6 | 19,363,830 | 5,799,030,798 | 97.08 | 38.08 | 18.99 | 98.56% |
| XW-9 | 16,755,089 | 5,017,982,156 | 96.81 | 37.66 | 16.43 | 98.54% |
| HL-1 | 17,901,841 | 5,361,330,444 | 97.62 | 38.04 | 17.55 | 88.32% |
| HL-2 | 15,587,109 | 4,668,092,968 | 97.84 | 37.90 | 15.28 | 94.38% |
| HL-4 | 17,819,470 | 5,336,604,942 | 95.97 | 37.90 | 17.47 | 98.52% |
| HH-2 | 16,428,691 | 4,920,258,792 | 98.14 | 37.72 | 16.11 | 98.49% |
| HH-3 | 16,710,703 | 5,004,237,184 | 96.87 | 37.76 | 16.38 | 97.89% |
| HH-10 | 16,364,526 | 4,901,376,256 | 98.19 | 37.99 | 16.05 | 98.37% |
| HD-7 | 18,729,599 | 5,609,276,984 | 96.87 | 38.49 | 18.36 | 98.58% |
| HD-8 | 17,487,797 | 5,237,635,670 | 97.87 | 38.42 | 17.15 | 97.65% |
| YC-1 | 19,365,491 | 5,799,605,346 | 97.54 | 38.00 | 18.99 | 98.68% |
| YC-2 | 15,091,886 | 4,519,638,594 | 98.11 | 37.41 | 14.80 | 97.62% |
| YC-5 | 16,039,050 | 4,800,975,550 | 98.21 | 37.65 | 15.72 | 98.44% |
| SF-2 | 15,032,070 | 4,500,157,300 | 98.01 | 38.00 | 14.73 | 98.37% |
| SF-6 | 16,556,257 | 4,956,459,356 | 98.13 | 37.87 | 16.23 | 98.41% |
| SF-8 | 16,239,898 | 4,861,530,500 | 98.01 | 37.64 | 15.92 | 98.16% |
| ZG-1 | 17,291,995 | 5,177,529,202 | 97.96 | 38.37 | 16.95 | 97.75% |
| ZG-4 | 15,159,492 | 4,537,589,450 | 97.86 | 37.43 | 14.86 | 97.13% |
| ZG-5 | 16,225,349 | 4,857,872,108 | 97.86 | 38.07 | 15.90 | 97.68% |
| JQ-1 | 17,707,032 | 5,303,149,128 | 96.91 | 38.02 | 17.36 | 98.07% |
| JQ-2 | 14,964,832 | 4,481,453,266 | 97.19 | 37.97 | 14.67 | 98.40% |
| JQ-4 | 15,036,088 | 4,503,159,660 | 97.07 | 38.07 | 14.74 | 98.30% |
| *A. amurensis* | 14,538,668 | 4,354,053,544 | 97.76 | 38.11 | 14.25 | 98.72% |
| *A. amurensis* | 14,758,620 | 4,419,626,304 | 97.17 | 38.10 | 14.47 | 98.71% |
| *A. japonica* | 27,165,900 | 2,444,931,000 | 96.65 | 37.68 | 8.00 | 97.96% |
| *A. japonica* | 28,247,416 | 2,542,267,440 | 96.72 | 38.00 | 8.32 | 97.60% |
| *A. oxysepala* var. *oxysepala* | 25,895,204 | 2,330,568,360 | 96.81 | 38.92 | 7.63 | 95.33% |
| *A. oxysepala* var. *oxysepala* | 30,172,356 | 2,715,512,040 | 97.49 | 38.47 | 8.89 | 97.82% |
| *A. oxysepala* var. *kansuensis* | 17,062,862 | 5,109,109,266 | 98.12 | 38.15 | 16.73 | 98.49% |
| *A. oxysepala* var. *kansuensis* | 16,161,175 | 4,837,931,934 | 98.08 | 38.20 | 15.84 | 96.22% |
| *A. yabeana* | 18,296,460 | 5,477,331,684 | 97.24 | 38.56 | 17.93 | 96.52% |
| *A. yabeana* | 21,746,987 | 6,512,016,988 | 97.66 | 38.52 | 21.32 | 96.94% |
| *A. ecalcarata* | 16,159,854 | 4,838,404,938 | 96.72 | 38.56 | 15.84 | 95.64% |
| *A. ecalcarata* | 16,076,498 | 4,813,394,578 | 96.85 | 38.41 | 15.76 | 98.35% |
| *Paraquilegia microphylla* | 16,239,629 | 4,864,514,694 | 97.38 | 38.44 | 15.93 | 56.23% |

**Table S3.** Results of the D-statistics in *Aquilegia*

| **P1** | **P2** | **P3** | **P4** | **D-stat** | **Z** | **BABA** | **ABBA** |
| --- | --- | --- | --- | --- | --- | --- | --- |
| East | West | *A. yabeana* | outgroup | -0.0073 | -1.453 | 10041 | 10190 |
| East | West | *A. oxysepala* var. *kansuensis* | outgroup | -0.0026 | -0.622 | 9757 | 9808 |
| East | West | *A. ecalcarata* | outgroup | -0.0064 | -1.462 | 9711 | 9836 |
| East | West | *A. japonica* | outgroup | 0.0100 | 1.923 | 9032 | 8854 |
| East | West | *A. oxysepala* var. *oxysepala* | outgroup | 0.0104 | 2.030 | 8887 | 8705 |
| East | West | *A. amurensis* | outgroup | 0.0092 | 1.942 | 9043 | 8878 |

**Table S4.** Results of the HyDe

| **P1** | **Hybrid** | **P2** | **Z-score** | **P value** | **Gamma** |
| --- | --- | --- | --- | --- | --- |
| NE | SZ-1 | EL | 7.18±1.76 | 2.9e-08±7.38e-08 | 0.84±0.04 |
| NE | SZ-1 | CN | 8.92±2.88 | 1.6e-09±8.8e-09 | 0.85±0.06 |
| NE | SZ-1 | NW | 11.47±3.91 | 5.9e-09±3.25e-08 | 0.86±0.06 |
| NE | SZ-3 | EL | 7.97±1.92 | 8.4e-09±3.23e-08 | 0.83±0.04 |
| NE | SZ-3 | CN | 10.11±3.02 | 3.1e-09±1.76e-08 | 0.84±0.05 |
| NE | SZ-3 | NW | 11.12±3.58 | 2.4e-09±1.34e-08 | 0.86±0.06 |
| NE | SZ-6 | EL | 9.44±2.74 | 1.3e-08±5.37e-08 | 0.82±0.06 |
| NE | SZ-6 | CN | 12.21±4.13 | 6.1e-09±3.33e-08 | 0.82±0.07 |
| NE | SZ-6 | NW | 13.73±4.9 | 5.2e-09±3.36e-08 | 0.84±0.07 |
| NE | LT-4 | EL | 14.25±5.29 | 5.5e-09±3.79e-08 | 0.16±0.06 |
| NE | LT-5 | EL | 13.49±4.84 | 9e-10±6e-09 | 0.16±0.06 |
| NE | LT-1 | EL | 11.37±4.27 | 2.6e-09±1.5e-08 | 0.14±0.05 |
| NE | LT-1 | CN | 12.76±3.47 | 2.5e-09±1.99e-08 | 0.56±0.11 |
| NE | LT-1 | NW | 7.26±1.46 | 1.81e-08±4.58e-08 | 0.63±0.06 |
| NE | LT-4 | CN | 15.14±3.95 | 0.0±4e-10 | 0.57±0.09 |
| NE | LT-4 | NW | 7.95±2.07 | 1.19e-08±4.17e-08 | 0.72±0.08 |
| NE | LT-5 | CN | 14.08±3.94 | 8e-10±6.8e-09 | 0.59±0.08 |
| NE | LT-5 | NW | 7.83±1.68 | 3.3e-09±9.4e-09 | 0.7±0.05 |
| NE | YM-1 | EL | 6.53±1.17 | 2.65e-08±5.71e-08 | 0.44±0.13 |
| NE | YM-4 | EL | 7.42±1.61 | 1.18e-08±4e-08 | 0.43±0.14 |
| NE | YM-5 | EL | 9.66±2.94 | 4.7e-09±2.45e-08 | 0.37±0.12 |
| NE | XW-6 | EL | 6.05±0.83 | 3.46e-08±8.96e-08 | 0.55±0.08 |
| NE | YM-1 | CN | 11.44±3.86 | 7.1e-09±3.61e-08 | 0.22±0.06 |
| NE | YM-4 | CN | 14.26±4.29 | 3e-09±2.76e-08 | 0.26±0.08 |
| NE | YM-5 | CN | 16.88±5.34 | 1.7e-09±1.09e-08 | 0.25±0.09 |
| NE | HL-2 | CN | 6.2±0.91 | 3.13e-08±5.68e-08 | 0.52±0.09 |
| NE | XW-6 | CN | 12.13±3.27 | 2.1e-09±1.57e-08 | 0.4±0.12 |
| NE | XW-9 | CN | 9.6±2.57 | 6.2e-09±4.02e-08 | 0.41±0.13 |
| NE | XW-2 | CN | 9.95±2.75 | 2.5e-09±1.19e-08 | 0.47±0.13 |
| NE | YM-1 | NW | 8.94±2.73 | 7.7e-09±2.97e-08 | 0.68±0.2 |
| NE | YM-4 | NW | 11.18±3.28 | 2.7e-09±1.63e-08 | 0.65±0.21 |
| NE | YM-5 | NW | 13.33±4.31 | 2.7e-09±1.72e-08 | 0.62±0.23 |
| NE | HL-2 | NW | 30.22±12.08 | 1.1e-09±7.9e-09 | 0.34±0.13 |
| NE | HL-4 | NW | 29.46±9.9 | 4.1e-09±2.9e-08 | 0.32±0.1 |
| NE | XW-6 | NW | 24.12±8.44 | 2e-09±2.26e-08 | 0.46±0.18 |
| NE | XW-9 | NW | 21.38±7.19 | 8e-10±5.8e-09 | 0.5±0.18 |
| NE | XW-2 | NW | 25.26±9.54 | 3.2e-09±2.85e-08 | 0.44±0.17 |
| NE | HH-3 | NW | 31.65±12.0 | 1.6e-09±2.19e-08 | 0.29±0.1 |
| NE | HH-10 | NW | 30.47±10.75 | 1.8e-09±1.41e-08 | 0.28±0.09 |
| NE | HL-1 | NW | 28.18±7.72 | 1e-10±1.5e-09 | 0.3±0.08 |
| NE | HH-2 | NW | 28.56±7.9 | 8e-10±6.7e-09 | 0.26±0.07 |
| NE | HD-8 | NW | 25.14±6.71 | 2e-09±2.24e-08 | 0.19±0.05 |
| NE | HD-7 | NW | 20.29±4.25 | 0.0±0.0 | 0.18±0.04 |
| NE | XW-2 | EL | 5.0±0.0 | 2.823e-07±0.0 | 0.56±0.0 |
| NE | XW-9 | EL | 5.43±0.0 | 2.82e-08±0.0 | 0.59±0.0 |
| EL | SZ-1 | NW | 14.41±3.6 | 9e-10±9.4e-09 | 0.7±0.14 |
| CN | SZ-1 | NW | 11.76±3.85 | 4.4e-09±3.01e-08 | 0.67±0.16 |
| EL | SZ-3 | NW | 13.44±3.34 | 1e-10±6e-10 | 0.73±0.13 |
| CN | SZ-3 | NW | 11.64±3.47 | 8e-10±6.9e-09 | 0.7±0.14 |
| EL | SZ-6 | NW | 16.9±3.82 | 2e-10±4.1e-09 | 0.68±0.14 |
| EL | SZ-6 | CN | 5.99±0.6 | 1.7e-08±1.7e-08 | 0.65±0.04 |
| CN | SZ-6 | NW | 13.72±4.22 | 2.4e-09±1.47e-08 | 0.68±0.16 |
| EL | LT-1 | CN | 10.23±2.06 | 0.0±0.0 | 0.86±0.04 |
| EL | LT-1 | NW | 7.46±1.6 | 7.7e-09±2.55e-08 | 0.93±0.02 |
| EL | YM-1 | CN | 7.26±2.01 | 2.85e-08±6.03e-08 | 0.24±0.05 |
| EL | YM-4 | CN | 7.9±2.59 | 1.84e-08±4.67e-08 | 0.27±0.06 |
| EL | YM-5 | CN | 9.92±3.07 | 1.4e-09±1.15e-08 | 0.23±0.1 |
| EL | YM-1 | NW | 13.28±3.98 | 1.2e-09±1.68e-08 | 0.69±0.21 |
| EL | YM-4 | NW | 16.65±3.83 | 1e-09±1.7e-08 | 0.66±0.19 |
| EL | YM-5 | NW | 21.26±3.69 | 0.0±0.0 | 0.65±0.17 |
| EL | HL-2 | NW | 30.82±13.31 | 4e-10±3.1e-09 | 0.31±0.13 |
| EL | XW-6 | NW | 29.85±8.41 | 8e-10±9e-09 | 0.46±0.15 |
| EL | XW-9 | NW | 26.7±7.49 | 1e-10±1.6e-09 | 0.48±0.16 |
| EL | XW-2 | NW | 30.14±9.63 | 0.0±0.0 | 0.43±0.15 |
| EL | HL-1 | NW | 29.54±8.9 | 1.1e-09±1.37e-08 | 0.29±0.08 |
| EL | HL-4 | NW | 29.91±9.22 | 3e-10±3.8e-09 | 0.3±0.09 |
| EL | HH-3 | NW | 30.88±11.32 | 4.1e-09±2.99e-08 | 0.26±0.09 |
| EL | HH-10 | NW | 29.46±8.75 | 2.2e-09±2.16e-08 | 0.25±0.07 |
| EL | HH-2 | NW | 26.93±7.87 | 1.8e-09±1.8e-08 | 0.23±0.06 |
| EL | HD-7 | NW | 19.93±3.91 | 0.0±0.0 | 0.16±0.03 |
| EL | HD-8 | NW | 24.16±5.49 | 3.7e-09±3.03e-08 | 0.18±0.04 |
| EL | LT-4 | CN | 8.91±2.69 | 1.07e-08±2.1e-08 | 0.89±0.06 |
| EL | LT-4 | NW | 7.06±2.17 | 4.23e-08±8.14e-08 | 0.94±0.03 |
| CN | LT-4 | NW | 5.79±0.56 | 5.86e-08±9.84e-08 | 0.86±0.07 |
| EL | LT-5 | CN | 9.53±0.84 | 0.0±0.0 | 0.85±0.0 |
| EL | LT-5 | NW | 7.0±1.7 | 1.45e-08±5.22e-08 | 0.93±0.03 |
| CN | LT-5 | NW | 5.68±0.0 | 6.8e-09±0.0 | 0.78±0.0 |
| CN | YM-1 | NW | 14.12±4.73 | 2.5e-09±1.54e-08 | 0.77±0.14 |
| CN | YM-4 | NW | 15.99±5.48 | 5e-09±2.71e-08 | 0.76±0.15 |
| CN | YM-5 | NW | 19.33±6.78 | 2.2e-09±2.04e-08 | 0.76±0.14 |
| CN | HL-2 | NW | 31.57±12.84 | 0.0±1e-10 | 0.35±0.14 |
| CN | XW-6 | NW | 29.0±7.55 | 8e-10±8.7e-09 | 0.54±0.17 |
| CN | XW-9 | NW | 25.5±6.64 | 1e-10±1.3e-09 | 0.57±0.17 |
| CN | XW-2 | NW | 29.35±8.21 | 0.0±0.0 | 0.49±0.16 |
| CN | HL-1 | NW | 29.77±9.43 | 2.1e-09±1.92e-08 | 0.31±0.09 |
| CN | HL-4 | NW | 30.39±10.52 | 1.9e-09±1.41e-08 | 0.32±0.11 |
| CN | HH-3 | NW | 32.03±11.36 | 2.8e-09±2.1e-08 | 0.29±0.1 |
| CN | HH-2 | NW | 28.05±8.12 | 4e-09±3.04e-08 | 0.26±0.07 |
| CN | HH-10 | NW | 30.53±9.27 | 1.9e-09±2.08e-08 | 0.28±0.08 |
| CN | HD-7 | NW | 21.71±3.81 | 0.0±0.0 | 0.18±0.03 |
| CN | HD-8 | NW | 26.27±5.25 | 0.0±1e-10 | 0.2±0.04 |

**Table S5.** Polymorphisms in the chloroplast genome of *A. viridiflora* complex

| Population | Hd | Pi |
| --- | --- | --- |
| NE | 0.85 | 0.00008 |
| EL | 0.857 | 0.00014 |
| CN | 0.833 | 0.00019 |
| NW | 0.929 | 0.00024 |
| Total | 0.971 | 0.00022 |

**Table S6.** Haplotype in the chloroplast genome of *A. viridiflora* complex

| Hd | Individuals |
| --- | --- |
| H1 | YC-2, YC-5 |
| H2 | JQ-1, JQ-2, JQ-4 |
| H3 | YC-1 |
| H4 | HD-7, HD-8 |
| H5 | HL-4 |
| H6 | TS-2 |
| H7 | TS-4 |
| H8 | TS-3, TS-6 |
| H9 | YM-4 |
| H10 | YM-1, YM-5 |
| H11 | QB-5, QB-8, QB-9 |
| H12 | HL-1, HL-2 |
| H13 | ZG-1, ZG-4, ZG-5 |
| H14 | SF-2, SF-6, SF-8 |
| H15 | SZ-6 |
| H16 | SZ-1, SZ-3 |
| H17 | XW-2, XW-6, XW-9 |
| H18 | YT-1, YT-8, YT-9, YT-10 |
| H19 | TL-3, TL-8, TL-12 |
| H20 | HH-2, HH-3, HH-10 |
| H21 | YS-2, YS-4, YS-5 |
| H22 | MS-3, MS-4, MS-5, MS-6 |
| H23 | LT-1, LT-4, LT-5 |
| H24 | WD-2, WD-10 |
| H25 | WD-6, WD-8, WD-12 |
| H26 | LF-1, LF-2, LF-3 |
| H27 | AE-1, AE-2, AE-3, AE-4, AE-5 |

**Table S7.** Results of the D-statistics

| **P1** | **P2** | **P3** | **P4** | **D-stat** | **Z** | **BABA** | **ABBA** |
| --- | --- | --- | --- | --- | --- | --- | --- |
| NE | **EL** | **CN** | outgroup | -0.0938 | -10.418 | 6743 | 8139 |
| **CN** | NW | **NE** | outgroup | 0.1105 | 9.799 | 8898 | 7128 |
| **CN** | NW | **EL** | outgroup | 0.2002 | 15.687 | 9904 | 6601 |
| NE | EL | NW | outgroup | 0.0100 | 1.865 | 6916 | 6778 |

Lineages with gene flow were indicated in bold.

**Table S8.** The AIC value of different models shown in Figure S7.

| **Model** | **DeltaL** | **AIC** |
| --- | --- | --- |
| M1 | 82303.149 | 5288012 |
| M2 | 85237.286 | 5301524.53 |
| M3 | 87646.562 | 5312619.66 |
| M4 | 91714.619 | 5331353.75 |
| M5 | 87623.527 | 5312513.58 |
| M6 | 80901.944 | 5281559.54 |
| M7 | 78504.729 | 5270519.96 |
| M8 | 74454.259 | 5251866.86 |
| M9 | 73068.1 | 5245483.36 |
| M10 | 77095.392 | 5264029.72 |
| M11 | 74985.84 | 5254314.88 |
| M12 | 78423.026 | 5270143.7 |
| **M13** | **69874.738** | **5230777.38** |
| M14 | 90308.177 | 5324876.85 |
| M15 | 84423.849 | 5297778.52 |
| M16 | 74247.592 | 5250915.12 |
| M17 | 71016.664 | 5236036.15 |
| M18 | 77305.513 | 5264997.37 |
| M19 | 71689.569 | 5239134.99 |
| M20 | 76180.614 | 5259817.02 |

The smallest AIC value were indicated in bold.

**Table S9.** Inferred parameters estimates with 95% confidence intervals for the best-fitting demographic scenario modelled in fastasimcoal2. The AIC value of different models shown in Figure S8.

|  | **Mean** | **-95% HPD** | **+95% HPD** |
| --- | --- | --- | --- |
| NNE | 561674.18 | 511713.691 | 609321.25 |
| NEL | 99439.96 | 93667.786 | 105134.864 |
| NCN | 433093.9 | 401713.524 | 463188.545 |
| NNW | 385320.58 | 356698.796 | 412838.138 |
| NNL | 503383.48 | 449068.43 | 558100.953 |
| NCW | 749485.58 | 705358.753 | 791950.404 |
| NANC | 927588.76 | 912244.88 | 941034.141 |
| TNL | 168324.12 | 152733.372 | 183702.821 |
| TCW | 211364.9 | 195529.078 | 226826 |
| TANC | 239100.26 | 217406.837 | 267235.163 |
| YMIG | 190632.28 | 175718.396 | 205216.441 |
| OMIG | 229058.32 | 209949.485 | 250886.477 |
| MIG10 | 2.30E-08 | 1.00E-08 | 4.00E-08 |
| MIG20 | 4.42E-07 | 1.91E-07 | 8.88E-07 |
| MIG01 | 3.57E-06 | 3.23E-06 | 3.96E-06 |
| MIG02 | 2.28E-07 | 1.65E-07 | 2.94E-07 |
| MIG21 | 2.60E-06 | 2.33E-06 | 2.90E-06 |
| MIG12 | 1.10E-07 | 3.80E-08 | 2.32E-07 |
| Y_MIG03 | 2.96E-07 | 1.33E-07 | 4.95E-07 |
| Y_MIG30 | 1.71E-07 | 9.40E-08 | 2.62E-07 |
| Y_MIG02 | 3.34E-07 | 1.59E-07 | 5.46E-07 |
| Y_MIG20 | 2.61E-07 | 1.02E-07 | 4.87E-07 |
| O_MIG02 | 7.40E-08 | 4.20E-08 | 1.12E-07 |
| O_MIG20 | 1.84E-07 | 7.80E-08 | 3.13E-07 |

**Table S10. The gene list under the selection.**

| **Gene ID** | **SNP** | | **F_ST_** | **qval** | **Best Arabidopsis hit** | **Gene name** |
| --- | --- | --- | --- | --- | --- | --- |
|  | **Chromosome** | **Location** |  |  |  |  |
| *Aqcoe1G004500* | 1 | 247494 | 0.37865 | 0.0090731 |  |  |
| *Aqcoe1G005900* | 1 | 317134 | 0.40247 | 0.0012688 | AT1G65440.2 | GTB1 |
| *Aqcoe1G010900* | 1 | 598515 | 0.32321 | 0.030832 | AT3G56930.1 |  |
| *Aqcoe1G017700* | 1 | 907819 | 0.38458 | 0.001375 | AT5G06800.1 |  |
| *Aqcoe1G018600* | 1 | 960210 | 0.38554 | 0.0030653 |  |  |
| *Aqcoe1G032200* | 1 | 1700358 | 0.38177 | 0.0074315 | AT3G08010.1 | AB2 |
| *Aqcoe1G042200* | 1 | 2161679 | 0.35986 | 0.017771 |  |  |
| *Aqcoe1G047100* | 1 | 2354585 | 0.31387 | 0.033242 |  |  |
| *Aqcoe1G063700* | 1 | 3235661 | 0.37754 | 0.0091113 | AT3G03760.1 | LBD20 |
| *Aqcoe1G066000* | 1 | 3363634 | 0.36674 | 0.031001 | AT3G03773.1 |  |
| *Aqcoe1G071400* | 1 | 3726610 | 0.32774 | 0.022384 | AT5G06560.1 |  |
| *Aqcoe1G075600* | 1 | 3966823 | 0.34241 | 0.012776 | AT5G22050.2 |  |
| *Aqcoe1G081400* | 1 | 4327887 | 0.34896 | 0.026453 | AT5G52340.1 | EXO70A2 |
| *Aqcoe1G084100* | 1 | 4479845 | 0.34795 | 0.04579 | AT4G11400.1 |  |
| *Aqcoe1G085100* | 1 | 4540929 | 0.30536 | 0.039679 | AT2G38060.1 | PHT4;2 |
| *Aqcoe1G096000* | 1 | 5093355 | 0.38582 | 0.00095284 | AT2G39840.1 | TOPP4 |
| *Aqcoe1G102200* | 1 | 5511235 | 0.38481 | 0.0034123 | AT3G47570.1 |  |
| *Aqcoe1G105000* | 1 | 5607822 | 0.40302 | 0.0011448 | AT5G26710.1 |  |
| *Aqcoe1G112700* | 1 | 6118344 | 0.34837 | 0.030329 | AT1G08230.2 | GAT1 |
| *Aqcoe1G121900* | 1 | 6670309 | 0.32926 | 0.020608 | AT2G36690.1 |  |
| *Aqcoe1G123800* | 1 | 6815452 | 0.3951 | 0.0014318 | AT2G36810.1 |  |
| *Aqcoe1G126400* | 1 | 6949488 | 0.35113 | 0.023647 |  |  |
| *Aqcoe1G147100* | 1 | 8279146 | 0.32103 | 0.029328 | AT3G52850.1 | ELP |
| *Aqcoe1G170200* | 1 | 9813579 | 0.41353 | 0 | AT1G31260.1 | ZIP10 |
| *Aqcoe1G172500* | 1 | 9987552 | 0.52361 | 0 | AT1G31330.1 | PSAF |
| *Aqcoe1G199400* | 1 | 11826869 | 0.35861 | 0.0050606 | AT3G10110.1 | MEE67 |
| *Aqcoe1G212700* | 1 | 12714591 | 0.4006 | 0.0011448 | AT1G06620.1 |  |
| *Aqcoe1G218900* | 1 | 13208916 | 0.3972 | 0.010388 | AT1G06720.1 |  |
| *Aqcoe1G223700* | 1 | 13612473 | 0.34171 | 0.028997 | AT1G64160.1 |  |
| *Aqcoe1G231800* | 1 | 14386265 | 0.37813 | 0.029494 | AT1G11740.1 |  |
| *Aqcoe1G232600* | 1 | 14492366 | 0.46634 | 0 | AT3G14550.1 | GGPS3 |
| *Aqcoe1G243700* | 1 | 15468184 | 0.37298 | 0.0031071 | AT1G78760.1 |  |
| *Aqcoe1G248700* | 1 | 15987051 | 0.47435 | 0.00017911 | AT3G62150.1 | PGP21 |
| *Aqcoe1G252200* | 1 | 16434289 | 0.29967 | 0.045455 | AT5G46110.4 | APE2 |
| *Aqcoe1G253600* | 1 | 16610292 | 0.3943 | 0.01639 | AT5G44200.2 | CBP20 |
| *Aqcoe1G255600* | 1 | 16835011 | 0.39856 | 0.00017911 | AT4G27540.1 | PRA1.H |
| *Aqcoe1G256700* | 1 | 16986922 | 0.3498 | 0.028335 | AT5G53900.2 |  |
| *Aqcoe1G257800* | 1 | 17124126 | 0.42389 | 0 | AT5G44280.1 | RING1A |
| *Aqcoe1G259500* | 1 | 17387550 | 0.31937 | 0.031428 | AT2G20650.1 |  |
| *Aqcoe1G260600* | 1 | 17469645 | 0.37343 | 0.0093809 | AT1G28280.1 |  |
| *Aqcoe1G261800* | 1 | 17633511 | 0.34635 | 0.033685 | AT4G27500.1 | PPI1 |
| *Aqcoe1G264100* | 1 | 17865629 | 0.34914 | 0.0081114 | AT2G20680.1 |  |
| *Aqcoe1G265600* | 1 | 18031131 | 0.3257 | 0.01968 | AT1G53025.1 |  |
| *Aqcoe1G275800* | 1 | 19438461 | 0.40261 | 0.0012688 |  |  |
| *Aqcoe1G278700* | 1 | 19801400 | 0.36144 | 0.041221 | AT1G50740.1 |  |
| *Aqcoe1G279800* | 1 | 19879331 | 0.35413 | 0.0064452 | AT4G21390.1 | B120 |
| *Aqcoe1G285200* | 1 | 20604419 | 0.37681 | 0.001054 | AT3G12060.1 | TBL1 |
| *Aqcoe1G297900* | 1 | 22876381 | 0.37079 | 0.0080394 | AT1G11930.2 |  |
| *Aqcoe1G298600* | 1 | 22972597 | 0.33535 | 0.022018 | AT3G20800.1 |  |
| *Aqcoe1G298700* | 1 | 22988386 | 0.37361 | 0.010552 |  |  |
| *Aqcoe1G301100* | 1 | 23591613 | 0.34804 | 0.005319 | AT1G02080.1 |  |
| *Aqcoe1G302300* | 1 | 23774427 | 0.42699 | 0.00027922 | AT5G54310.1 | AGD5 |
| *Aqcoe1G305900* | 1 | 24968600 | 0.42901 | 0.0019821 | AT1G16280.1 | RH36 |
| *Aqcoe1G306300* | 1 | 25247268 | 0.43363 | 0 |  |  |
| *Aqcoe1G308600* | 1 | 25738915 | 0.35876 | 2.94E-03 | AT2G20810.1 | GAUT10 |
| *Aqcoe1G310200* | 1 | 26070543 | 0.44685 | 0 | AT1G07350.1 |  |
| *Aqcoe1G313200* | 1 | 26806073 | 0.32668 | 0.018526 | AT5G47690.3 |  |
| *Aqcoe1G314700* | 1 | 27402656 | 0.4968 | 0 | AT2G20760.1 |  |
| *Aqcoe1G318100* | 1 | 27889473 | 0.30842 | 0.041535 | AT2G14830.1 |  |
| *Aqcoe1G318600* | 1 | 27996107 | 0.40145 | 0.00095284 |  |  |
| *Aqcoe1G319300* | 1 | 28074379 | 0.34824 | 0.027594 | AT4G27340.1 |  |
| *Aqcoe1G330100* | 1 | 29720978 | 0.3972 | 0.015287 | AT4G38180.1 | FRS5 |
| *Aqcoe1G332600* | 1 | 30151399 | 0.38686 | 0.00077267 | AT2G28470.1 | BGAL8 |
| *Aqcoe1G333900* | 1 | 30387644 | 0.45127 | 8.49E-05 | AT2G04740.1 |  |
| *Aqcoe1G336000* | 1 | 30826520 | 0.38422 | 0.0037621 | AT5G05390.1 | LAC12 |
| *Aqcoe1G339200* | 1 | 31560940 | 0.3638 | 0.014058 |  |  |
| *Aqcoe1G342000* | 1 | 32158771 | 0.35425 | 0.0062286 | AT5G55130.1 | CNX5 |
| *Aqcoe1G353500* | 1 | 33710916 | 0.35359 | 0.0051734 | AT4G27100.2 |  |
| *Aqcoe1G360000* | 1 | 34380809 | 0.41641 | 0.00046073 | AT5G54800.1 | GPT1 |
| *Aqcoe1G362400* | 1 | 34716770 | 0.30239 | 0.040599 | AT3G21810.1 |  |
| *Aqcoe1G364100* | 1 | 34967477 | 0.34539 | 0.025101 | AT4G05320.1 | UBQ10 |
| *Aqcoe1G370700* | 1 | 35959840 | 0.32871 | 0.016096 | AT5G54580.1 |  |
| *Aqcoe1G377700* | 1 | 36527452 | 0.35652 | 0.0042488 | AT4G26550.1 |  |
| *Aqcoe1G384200* | 1 | 37197341 | 0.39638 | 0.0015927 | AT1G55900.1 | emb1860 |
| *Aqcoe1G390200* | 1 | 37643980 | 0.36004 | 0.018398 |  |  |
| *Aqcoe1G390900* | 1 | 37717491 | 0.37597 | 0.026534 | AT5G61140.2 |  |
| *Aqcoe1G395800* | 1 | 38103024 | 0.36649 | 0.0017348 | AT4G16710.2 |  |
| *Aqcoe1G405400* | 1 | 38728784 | 0.38646 | 4.68E-03 | AT1G32750.1 | GTD1 |
| *Aqcoe1G417400* | 1 | 39577729 | 0.35632 | 0.0041187 | AT3G51840.1 | ACX4 |
| *Aqcoe1G418300* | 1 | 39631769 | 0.41829 | 0.00038965 | AT5G35370.1 |  |
| *Aqcoe1G422800* | 1 | 39924939 | 0.32895 | 0.046465 | AT3G51870.1 |  |
| *Aqcoe1G423800* | 1 | 40012484 | 0.33039 | 0.015518 | AT2G29940.1 | PDR3 |
| *Aqcoe1G427900* | 1 | 40242151 | 0.34826 | 0.0062902 | AT5G05760.1 | SED5 |
| *Aqcoe1G431700* | 1 | 40541919 | 0.34743 | 0.025651 | AT2G39940.1 | COI1 |
| *Aqcoe1G432400* | 1 | 40587954 | 0.41625 | 0.00088163 | AT1G05380.1 |  |
| *Aqcoe1G436400* | 1 | 40833228 | 0.34902 | 0.022825 | AT5G13480.1 | FY |
| *Aqcoe1G444600* | 1 | 41275726 | 0.36937 | 0.0030653 | AT3G52720.1 | ACA1 |
| *Aqcoe1G445900* | 1 | 41334669 | 0.3813 | 0.0050887 | AT5G20090.2 |  |
| *Aqcoe1G448500* | 1 | 41494299 | 0.44073 | 8.49E-05 | AT5G04710.1 |  |
| *Aqcoe1G453800* | 1 | 41836753 | 0.40918 | 0.0041187 | AT5G03650.1 | SBE2.2 |
| *Aqcoe1G475700* | 1 | 43110423 | 0.33541 | 0.016213 | AT5G13550.1 | SULTR4;1 |
| *Aqcoe1G480600* | 1 | 43384787 | 0.32058 | 0.030246 | AT3G55070.1 |  |
| *Aqcoe1G486700* | 1 | 43729157 | 0.4115 | 0.00083869 |  |  |
| *Aqcoe1G487800* | 1 | 43827373 | 0.62085 | 0 | AT5G60170.2 |  |
| *Aqcoe1G488100* | 1 | 43841163 | 0.35604 | 0.039477 | AT1G07890.8 | APX1 |
| *Aqcoe1G488500* | 1 | 43863153 | 0.33953 | 0.010808 | AT2G37050.3 |  |
| *Aqcoe1G488600* | 1 | 43867201 | 0.36771 | 0.01218 | AT3G45660.1 |  |
| *Aqcoe1G493000* | 1 | 44096165 | 0.33601 | 0.018081 | AT5G60410.5 | SIZ1 |
| *Aqcoe2G001000* | 2 | 155474 | 0.34053 | 0.010594 | AT3G28910.1 | MYB30 |
| *Aqcoe2G002900* | 2 | 288776 | 0.33903 | 0.032372 |  |  |
| *Aqcoe2G012900* | 2 | 914342 | 0.34949 | 0.04501 | AT5G64670.1 |  |
| *Aqcoe2G023600* | 2 | 1622592 | 0.3268 | 0.025101 | AT3G63470.1 | scpl40 |
| *Aqcoe2G025000* | 2 | 1732118 | 0.37415 | 0.026373 | AT5G12430.1 |  |
| *Aqcoe2G028300* | 2 | 1897286 | 0.33895 | 0.031599 |  |  |
| *Aqcoe2G035600* | 2 | 2371128 | 0.41926 | 1.79E-04 | AT1G15520.1 | ABCG40 |
| *Aqcoe2G043200* | 2 | 2907477 | 0.38992 | 3.55E-03 | AT1G43190.2 | PTB3 |
| *Aqcoe2G043600* | 2 | 2930283 | 0.32416 | 0.049436 | AT3G12250.2 | BZIP45 |
| *Aqcoe2G045600* | 2 | 3072092 | 0.44147 | 0 |  |  |
| *Aqcoe2G048200* | 2 | 3273723 | 0.39059 | 0.0034123 |  |  |
| *Aqcoe2G052800* | 2 | 3545128 | 0.38516 | 0.0033013 | AT3G11980.1 | FAR2 |
| *Aqcoe2G053600* | 2 | 3622823 | 0.32685 | 0.044789 | AT3G11980.1 | FAR2 |
| *Aqcoe2G085700* | 2 | 6329979 | 0.34868 | 0.028171 |  |  |
| *Aqcoe2G091600* | 2 | 6772953 | 0.3103 | 0.035327 | AT5G05580.1 | FAD8 |
| *Aqcoe2G098900* | 2 | 7355132 | 0.36917 | 0.012776 | AT2G01050.1 |  |
| *Aqcoe2G099800* | 2 | 7419591 | 0.35142 | 2.77E-02 | AT5G12370.2 | SEC10 |
| *Aqcoe2G103700* | 2 | 7749272 | 0.34725 | 0.024484 | AT2G38080.1 | LMCO4 |
| *Aqcoe2G103800* | 2 | 7787285 | 0.37486 | 0.00088163 | AT2G38080.1 | LMCO4 |
| *Aqcoe2G105000* | 2 | 8130080 | 0.33375 | 0.037599 | AT1G48090.1 |  |
| *Aqcoe2G111200* | 2 | 9005039 | 0.38635 | 0.0069531 | AT3G24495.1 | MSH7 |
| *Aqcoe2G114100* | 2 | 9326194 | 0.31961 | 0.030079 | AT2G36130.1 |  |
| *Aqcoe2G121300* | 2 | 9920011 | 0.37242 | 0.0095771 | AT3G07040.1 | RPM1 |
| *Aqcoe2G126100* | 2 | 10519741 | 0.43023 | 4.06E-05 | AT2G36480.3 |  |
| *Aqcoe2G130200* | 2 | 11010846 | 0.43361 | 8.49E-05 | AT1G66950.1 | PDR11 |
| *Aqcoe2G130400* | 2 | 11090185 | 0.40239 | 0.0065702 | AT5G22510.1 | A/N-InvE |
| *Aqcoe2G135000* | 2 | 12142785 | 0.38118 | 0.001054 | AT3G51970.1 | ASAT1 |
| *Aqcoe2G135700* | 2 | 12248423 | 0.38144 | 6.79E-03 | AT3G44540.1 | FAR4 |
| *Aqcoe2G136200* | 2 | 12317650 | 0.37591 | 0.0083661 | AT3G44530.1 | HIRA |
| *Aqcoe2G144000* | 2 | 13595757 | 0.35919 | 0.039076 | AT2G37010.1 | NAP12 |
| *Aqcoe2G165200* | 2 | 18485728 | 0.41053 | 0.01319 | AT5G41020.1 |  |
| *Aqcoe2G167300* | 2 | 19537699 | 0.56094 | 0 | AT1G07890.3 | APX1 |
| *Aqcoe2G170700* | 2 | 20700164 | 0.45318 | 0.00064566 | AT5G20660.1 |  |
| *Aqcoe2G171100* | 2 | 20760057 | 0.45233 | 0.00E+00 |  |  |
| *Aqcoe2G171500* | 2 | 20860115 | 0.39563 | 0.01598 |  |  |
| *Aqcoe2G171700* | 2 | 20905478 | 0.44772 | 4.06E-05 | AT1G07670.1 | ECA4 |
| *Aqcoe2G173200* | 2 | 21156363 | 0.4119 | 0.00027922 | AT3G42050.1 |  |
| *Aqcoe2G176400* | 2 | 21591037 | 0.3327 | 0.037309 |  |  |
| *Aqcoe2G176800* | 2 | 21682179 | 0.35251 | 0.0218 | AT3G12520.1 | SULTR4;2 |
| *Aqcoe2G176900* | 2 | 21711864 | 0.34268 | 0.032372 | AT3G55260.1 | HEX2 |
| *Aqcoe2G180300* | 2 | 22123922 | 0.37209 | 0.001375 | AT5G59880.1 | ADF3 |
| *Aqcoe2G181300* | 2 | 22236508 | 0.37646 | 0.010679 |  |  |
| *Aqcoe2G196600* | 2 | 23519111 | 0.29233 | 0.049551 |  |  |
| *Aqcoe2G202600* | 2 | 24121333 | 0.41206 | 0.0067593 | AT3G48930.1 | EMB1080 |
| *Aqcoe2G203600* | 2 | 24361285 | 0.39094 | 0.00077267 |  |  |
| *Aqcoe2G204600* | 2 | 24534378 | 0.32528 | 0.036924 | AT3G46100.1 | HRS1 |
| *Aqcoe2G219600* | 2 | 27543599 | 0.35031 | 0.0085131 | AT2G36000.1 |  |
| *Aqcoe2G231600* | 2 | 29006249 | 0.44123 | 0 | AT3G55580.1 |  |
| *Aqcoe2G232900* | 2 | 29113808 | 0.30281 | 0.043687 | AT2G37860.3 | LCD1 |
| *Aqcoe2G236900* | 2 | 29568486 | 0.46387 | 0 | AT1G09560.1 | GLP5 |
| *Aqcoe2G241000* | 2 | 29982246 | 0.37865 | 0.024179 | AT1G09530.2 | PAP3 |
| *Aqcoe2G247700* | 2 | 30600099 | 0.34529 | 0.011573 | AT1G48280.1 |  |
| *Aqcoe2G260500* | 2 | 31852931 | 0.46094 | 0.00038965 | AT5G64250.2 |  |
| *Aqcoe2G265800* | 2 | 32380822 | 0.34357 | 0.039277 | AT1G09320.1 |  |
| *Aqcoe2G272000* | 2 | 32917801 | 0.4344 | 0.0015927 | AT1G64520.1 | RPN12a |
| *Aqcoe2G274000* | 2 | 33073077 | 0.35774 | 0.040085 | AT3G62390.1 | TBL6 |
| *Aqcoe2G278000* | 2 | 33488441 | 0.40014 | 4.06E-05 | AT3G62580.1 |  |
| *Aqcoe2G279700* | 2 | 33679468 | 0.35874 | 0.015173 | AT4G02120.1 |  |
| *Aqcoe2G280700* | 2 | 33800278 | 0.35804 | 0.019358 | AT4G25800.1 |  |
| *Aqcoe2G287700* | 2 | 34282431 | 0.40575 | 0.0013106 | AT2G04160.1 | AIR3 |
| *Aqcoe2G289300* | 2 | 34432577 | 0.32297 | 0.047262 |  |  |
| *Aqcoe2G295200* | 2 | 34823733 | 0.29622 | 0.046015 | AT1G02850.2 | BGLU11 |
| *Aqcoe2G305100* | 2 | 35521302 | 0.33244 | 0.038477 | AT2G47810.1 | NF-YB5 |
| *Aqcoe2G309800* | 2 | 35881586 | 0.35446 | 0.022091 | AT3G09030.1 |  |
| *Aqcoe2G310500* | 2 | 35924612 | 0.37862 | 0.009188 | AT3G05040.1 | HST |
| *Aqcoe2G313400* | 2 | 36086379 | 0.42625 | 0.00038965 | AT3G26932.1 | DRB3 |
| *Aqcoe2G315500* | 2 | 36201068 | 0.34577 | 0.029911 | AT1G08830.1 | CSD1 |
| *Aqcoe2G319800* | 2 | 36475248 | 0.37477 | 0.0072576 | AT2G30780.1 |  |
| *Aqcoe2G333800* | 2 | 37250236 | 0.3488 | 0.023271 | AT5G55720.1 |  |
| *Aqcoe2G339800* | 2 | 37574886 | 0.30861 | 0.045344 | AT3G05990.1 |  |
| *Aqcoe2G340700* | 2 | 37611481 | 0.29727 | 0.046807 | AT5G26240.1 | CLC-D |
| *Aqcoe2G345200* | 2 | 37859416 | 0.3942 | 0.0040161 | AT4G14920.1 |  |
| *Aqcoe2G367100* | 2 | 39118732 | 0.35714 | 0.0035494 | AT5G42670.1 |  |
| *Aqcoe2G369800* | 2 | 39270440 | 0.32114 | 0.032114 | AT5G43500.2 | ARP9 |
| *Aqcoe2G377000* | 2 | 39681593 | 0.35146 | 0.019616 | AT2G18760.1 | CHR8 |
| *Aqcoe2G379800* | 2 | 39836249 | 0.40986 | 0.00046073 | AT2G20420.1 |  |
| *Aqcoe2G380100* | 2 | 39877183 | 0.3208 | 2.54E-02 |  |  |
| *Aqcoe2G382100* | 2 | 39965968 | 0.42115 | 2.07E-04 | AT3G04710.3 |  |
| *Aqcoe2G385000* | 2 | 40108220 | 0.37136 | 1.13E-02 | AT2G33320.1 |  |
| *Aqcoe2G408900* | 2 | 41299529 | 0.35609 | 1.58E-02 | AT5G48480.1 |  |
| *Aqcoe2G409800* | 2 | 41357346 | 0.33356 | 0.016926 | AT3G25290.2 |  |
| *Aqcoe2G432300* | 2 | 42739838 | 0.40164 | 0.0018308 | AT1G65480.1 | FT |
| *Aqcoe2G432600* | 2 | 42766110 | 0.33094 | 0.015864 |  |  |
| *Aqcoe2G434700* | 2 | 42872788 | 0.33416 | 0.034316 | AT5G17440.1 |  |
| *Aqcoe2G437900* | 2 | 43082249 | 0.44445 | 0.00017911 | AT5G48380.1 | BIR1 |
| *Aqcoe3G003100* | 3 | 218727 | 0.35791 | 0.017771 |  |  |
| *Aqcoe3G004600* | 3 | 293722 | 0.40065 | 0.00027922 | AT4G11560.1 |  |
| *Aqcoe3G006600* | 3 | 387261 | 0.31427 | 0.027186 | AT3G17850.1 |  |
| *Aqcoe3G015400* | 3 | 979325 | 0.37188 | 0.001375 | AT1G56070.1 | LOS1 |
| *Aqcoe3G016500* | 3 | 1028621 | 0.33216 | 0.038676 | AT1G57790.1 |  |
| *Aqcoe3G040700* | 3 | 2602559 | 0.3969 | 0.0006922 |  |  |
| *Aqcoe3G042200* | 3 | 2684191 | 0.58516 | 0 | AT4G36220.1 | CYP84A1 |
| *Aqcoe3G049200* | 3 | 3108995 | 0.30869 | 0.045232 |  |  |
| *Aqcoe3G049700* | 3 | 3129712 | 0.37669 | 0.009188 | AT3G18520.2 | HDA15 |
| *Aqcoe3G052500* | 3 | 3296413 | 0.32704 | 0.043468 |  |  |
| *Aqcoe3G054300* | 3 | 3443709 | 0.37114 | 0.0034578 | AT1G26120.1 | ICME-LIKE1 |
| *Aqcoe3G063700* | 3 | 3949887 | 0.40053 | 6.92E-04 | AT1G69210.1 |  |
| *Aqcoe3G063900* | 3 | 3967537 | 0.31252 | 3.51E-02 | AT2G03667.1 |  |
| *Aqcoe3G070000* | 3 | 4407669 | 0.29968 | 0.049093 | AT5G15710.1 |  |
| *Aqcoe3G071900* | 3 | 4537800 | 0.39541 | 0.011162 | AT5G49610.1 |  |
| *Aqcoe3G094800* | 3 | 6320795 | 0.38755 | 0.018847 | AT4G28480.1 |  |
| *Aqcoe3G113800* | 3 | 7659559 | 0.31601 | 0.02581 | AT1G60070.1 |  |
| *Aqcoe3G114800* | 3 | 7725199 | 0.37142 | 0.027349 | AT5G14870.1 | CNGC18 |
| *Aqcoe3G121100* | 3 | 8495762 | 0.41693 | 0.0005713 | AT2G26540.1 | DUF3 |
| *Aqcoe3G122000* | 3 | 8584622 | 0.35722 | 0.016628 | AT3G01500.3 | BCA1 |
| *Aqcoe3G129400* | 3 | 9288068 | 0.31347 | 0.033242 | AT5G63270.1 |  |
| *Aqcoe3G132200* | 3 | 9642045 | 0.44839 | 4.06E-05 | AT3G48430.1 | REF6 |
| *Aqcoe3G133800* | 3 | 9811727 | 0.35342 | 0.02395 | AT5G14370.1 |  |
| *Aqcoe3G141100* | 3 | 10586718 | 0.5428 | 0 | AT5G14450.1 |  |
| *Aqcoe3G141300* | 3 | 10598531 | 0.35769 | 0.014113 |  |  |
| *Aqcoe3G149700* | 3 | 12135227 | 0.32346 | 0.020608 | AT1G17720.1 | ATB BETA |
| *Aqcoe3G151300* | 3 | 12338025 | 0.35414 | 0.0093036 | AT4G24680.1 | MOS1 |
| *Aqcoe3G153000* | 3 | 12571972 | 0.40298 | 0.0005713 | AT1G78770.1 | APC6 |
| *Aqcoe3G155900* | 3 | 13196027 | 0.37634 | 0.031343 | AT5G14010.1 | KNU |
| *Aqcoe3G156600* | 3 | 13389106 | 0.40049 | 0.00017911 | AT3G13080.1 | MRP3 |
| *Aqcoe3G157200* | 3 | 13513013 | 0.373 | 0.02743 | AT4G00990.1 |  |
| *Aqcoe3G158900* | 3 | 13709176 | 0.49319 | 0 | AT3G26935.1 |  |
| *Aqcoe3G159400* | 3 | 13812431 | 0.41948 | 0.00020678 | AT5G23660.1 | MTN3 |
| *Aqcoe3G163200* | 3 | 14303291 | 0.35407 | 0.0070192 |  |  |
| *Aqcoe3G164100* | 3 | 14444031 | 0.36804 | 0.010388 | AT5G49510.1 | PFD3 |
| *Aqcoe3G165800* | 3 | 14779288 | 0.38133 | 0.00038965 | AT1G32340.1 | NHL8 |
| *Aqcoe3G169700* | 3 | 15302289 | 0.48373 | 0 | AT1G32240.1 | KAN2 |
| *Aqcoe3G172300* | 3 | 15932040 | 0.32477 | 0.024102 | AT1G32160.1 |  |
| *Aqcoe3G173000* | 3 | 16140270 | 0.36876 | 0.0016815 |  |  |
| *Aqcoe3G173200* | 3 | 16155923 | 0.35834 | 0.041117 | AT3G57180.1 | BPG2 |
| *Aqcoe3G175400* | 3 | 16618222 | 0.57513 | 0 | AT4G08455.1 |  |
| *Aqcoe3G176000* | 3 | 16760076 | 0.31214 | 0.031172 | AT5G51200.1 |  |
| *Aqcoe3G177800* | 3 | 17251943 | 0.43028 | 0.0015927 |  |  |
| *Aqcoe3G178500* | 3 | 17429507 | 0.43745 | 7.73E-04 | AT4G26965.1 |  |
| *Aqcoe3G187100* | 3 | 19548701 | 0.41136 | 0.0040161 | AT1G31360.1 | RECQ2 |
| *Aqcoe3G189000* | 3 | 19772656 | 0.3304 | 0.022238 |  |  |
| *Aqcoe3G199900* | 3 | 21163955 | 0.36053 | 0.0023486 | AT4G30480.2 |  |
| *Aqcoe3G206400* | 3 | 22409436 | 0.43916 | 0.0012688 |  |  |
| *Aqcoe3G209000* | 3 | 23414924 | 0.40124 | 0.0016685 |  |  |
| *Aqcoe3G213800* | 3 | 24432751 | 0.30978 | 0.03702 | AT5G27270.1 | EMB976 |
| *Aqcoe3G215000* | 3 | 24786023 | 0.35114 | 0.022605 | AT1G15520.1 | ABCG40 |
| *Aqcoe3G216300* | 3 | 24974067 | 0.41178 | 0.0041445 | AT1G31650.1 | ROPGEF14 |
| *Aqcoe3G216400* | 3 | 25001828 | 0.37037 | 0.0095771 |  |  |
| *Aqcoe3G217500* | 3 | 25423281 | 0.36284 | 0.034956 | AT3G48820.1 |  |
| *Aqcoe3G217800* | 3 | 25498023 | 0.33071 | 0.019486 | AT1G80170.1 |  |
| *Aqcoe3G242600* | 3 | 28917183 | 0.38952 | 0.005408 | AT5G07440.1 | GDH2 |
| *Aqcoe3G246900* | 3 | 29338015 | 0.34286 | 0.011344 | AT1G12920.1 | ERF1-2 |
| *Aqcoe3G273500* | 3 | 31973463 | 0.39037 | 0.0052599 | AT5G45140.1 | NRPC2 |
| *Aqcoe3G276300* | 3 | 32256224 | 0.42678 | 0.0022428 | AT4G24670.2 | TAR2 |
| *Aqcoe3G284600* | 3 | 32964022 | 0.33451 | 0.013948 |  |  |
| *Aqcoe3G298500* | 3 | 34044426 | 0.35626 | 0.0044062 | AT1G23820.1 | SPDS1 |
| *Aqcoe3G300900* | 3 | 34241829 | 0.3915 | 0.0027823 | AT1G60060.1 |  |
| *Aqcoe3G313100* | 3 | 35030380 | 0.34482 | 0.0080394 |  |  |
| *Aqcoe3G315200* | 3 | 35188705 | 0.33293 | 0.037986 | AT1G10430.1 | PP2A-2 |
| *Aqcoe3G329000* | 3 | 35980505 | 0.36853 | 0.0012688 | AT5G62530.1 | ALDH12A1 |
| *Aqcoe3G340200* | 3 | 36629922 | 0.38038 | 0.012038 | AT1G26810.1 | GALT1 |
| *Aqcoe3G344000* | 3 | 36841231 | 0.35284 | 0.0062286 | AT1G14180.1 |  |
| *Aqcoe3G344700* | 3 | 36883061 | 0.32257 | 0.047719 |  |  |
| *Aqcoe3G347700* | 3 | 37045995 | 0.35515 | 0.01981 | AT3G16660.1 |  |
| *Aqcoe3G347900* | 3 | 37045995 | 0.35515 | 0.01981 | AT3G16660.1 |  |
| *Aqcoe3G370100* | 3 | 38439483 | 0.39579 | 0.0026084 | AT3G02150.2 | PTF1 |
| *Aqcoe3G384400* | 3 | 39334721 | 0.41877 | 0.00088163 | AT1G13580.3 | LAG13 |
| *Aqcoe3G394700* | 3 | 39867171 | 0.41678 | 0.00046073 |  |  |
| *Aqcoe3G404100* | 3 | 40482091 | 0.54834 | 0 | AT5G08680.1 |  |
| *Aqcoe3G407000* | 3 | 40634770 | 0.44516 | 0 | AT2G27030.3 | ACAM-2 |
| *Aqcoe3G412500* | 3 | 40939588 | 0.33728 | 0.039881 | AT2G01940.1 | IDD15 |
| *Aqcoe3G414300* | 3 | 41039611 | 0.31429 | 0.036071 | AT1G24764.1 | MAP70-2 |
| *Aqcoe3G427000* | 3 | 41642474 | 0.29887 | 0.047262 | AT1G14570.2 |  |
| *Aqcoe3G427100* | 3 | 41654074 | 0.38839 | 0.0047573 | AT1G31050.1 |  |
| *Aqcoe3G436800* | 3 | 42256899 | 0.36104 | 0.0023486 | AT3G19820.2 | CBB1 |
| *Aqcoe3G439800* | 3 | 42426691 | 0.46943 | 0.00012408 | AT5G23340.1 |  |
| *Aqcoe3G440500* | 3 | 42478026 | 0.34659 | 0.027186 | AT1G50410.1 |  |
| *Aqcoe3G441400* | 3 | 42523207 | 0.45939 | 0 |  |  |
| *Aqcoe3G444600* | 3 | 42687185 | 0.31012 | 0.033864 | AT5G13240.1 |  |
| *Aqcoe4G010600* | 4 | 946553 | 0.40978 | 0.0005713 | AT3G18080.1 | BGLU44 |
| *Aqcoe4G053100* | 4 | 4668024 | 0.31838 | 0.033418 | AT3G59010.1 | PME61 |
| *Aqcoe4G116100* | 4 | 12650522 | 0.37923 | 0.0015927 | AT4G18550.1 |  |
| *Aqcoe4G160900* | 4 | 21619464 | 0.34217 | 0.02966 |  |  |
| *Aqcoe4G171100* | 4 | 23526004 | 0.38459 | 0.026212 |  |  |
| *Aqcoe4G180200* | 4 | 25209434 | 0.34712 | 0.028748 | AT1G72050.2 | TFIIIA |
| *Aqcoe4G188500* | 4 | 27293314 | 0.39869 | 0.011435 |  |  |
| *Aqcoe4G196300* | 4 | 29086841 | 0.44449 | 0 | AT5G35700.1 | FIM2 |
| *Aqcoe4G217500* | 4 | 34282465 | 0.39053 | 0.0037132 | AT1G15125.1 |  |
| *Aqcoe4G224000* | 4 | 35647807 | 0.35805 | 0.020268 | AT3G20390.1 |  |
| *Aqcoe4G224800* | 4 | 35720849 | 0.32189 | 0.027841 | AT1G62360.1 | BUM |
| *Aqcoe4G242300* | 4 | 39105401 | 0.36686 | 0.029246 | AT2G24050.1 | eIFiso4G2 |
| *Aqcoe4G249500* | 4 | 39986936 | 0.41395 | 0.00095284 | AT5G42830.1 |  |
| *Aqcoe4G259900* | 4 | 41091082 | 0.34508 | 0.016986 | AT5G54250.2 | CNGC4 |
| *Aqcoe4G261300* | 4 | 41204055 | 0.35014 | 0.043687 | AT4G21450.1 |  |
| *Aqcoe4G274500* | 4 | 42606005 | 0.34033 | 0.031685 | AT5G06900.1 | CYP93D1 |
| *Aqcoe4G281400* | 4 | 43067128 | 0.32655 | 0.044458 | AT5G55390.1 | EDM2 |
| *Aqcoe5G006000* | 5 | 369332 | 0.34366 | 0.0099377 | AT4G28760.2 |  |
| *Aqcoe5G007900* | 5 | 469019 | 0.43293 | 0.00017911 | AT2G32700.6 | LUH |
| *Aqcoe5G008000* | 5 | 478313 | 0.37364 | 0.0099784 | AT3G24820.1 |  |
| *Aqcoe5G010600* | 5 | 639412 | 0.3467 | 0.0056776 | AT5G43940.1 | ADH2 |
| *Aqcoe5G012500* | 5 | 752759 | 0.40448 | 1.24E-04 | AT1G04120.1 | ABCC5 |
| *Aqcoe5G025300* | 5 | 1425690 | 0.37565 | 0.00046073 | AT3G54670.3 | SMC1 |
| *Aqcoe5G032500* | 5 | 1739772 | 0.40755 | 0.0012688 | AT3G23150.1 | ETR2 |
| *Aqcoe5G034400* | 5 | 1872697 | 0.35252 | 0.043142 |  |  |
| *Aqcoe5G036500* | 5 | 1945222 | 0.3242 | 0.046352 | AT3G04610.1 | FLK |
| *Aqcoe5G043100* | 5 | 2249598 | 0.33408 | 0.013405 | AT3G54610.1 | BGT |
| *Aqcoe5G051600* | 5 | 2812455 | 0.32792 | 4.14E-02 | AT4G14805.1 |  |
| *Aqcoe5G070900* | 5 | 3806004 | 0.3047 | 4.26E-02 | AT2G47970.1 |  |
| *Aqcoe5G097900* | 5 | 5251774 | 0.38172 | 0.0023486 |  |  |
| *Aqcoe5G117400* | 5 | 6445383 | 0.36868 | 0.030162 |  |  |
| *Aqcoe5G121400* | 5 | 6661275 | 0.39561 | 0.0021416 | AT2G17570.1 |  |
| *Aqcoe5G145600* | 5 | 8134370 | 0.3197 | 2.61E-02 | AT4G36910.1 | CDCP2 |
| *Aqcoe5G152200* | 5 | 8588464 | 0.35224 | 0.0046759 | AT3G19270.1 | CYP707A4 |
| *Aqcoe5G158200* | 5 | 8992704 | 0.34977 | 0.022531 | AT5G53390.1 |  |
| *Aqcoe5G160300* | 5 | 9139612 | 0.34351 | 0.0065702 | AT5G64360.1 |  |
| *Aqcoe5G160900* | 5 | 9166496 | 0.36122 | 0.0044062 | AT2G39340.1 |  |
| *Aqcoe5G161700* | 5 | 9246907 | 0.37218 | 0.0093036 |  |  |
| *Aqcoe5G163200* | 5 | 9357298 | 0.34727 | 0.028748 | AT5G10940.2 |  |
| *Aqcoe5G165600* | 5 | 9567515 | 0.40995 | 0.0006922 | AT4G38180.1 | FRS5 |
| *Aqcoe5G168600* | 5 | 9742544 | 0.38689 | 1.52E-02 | AT5G64210.1 | AOX2 |
| *Aqcoe5G171800* | 5 | 9904524 | 0.32336 | 0.022164 | AT2G23540.1 |  |
| *Aqcoe5G177700* | 5 | 10268020 | 0.34644 | 0.0081835 |  |  |
| *Aqcoe5G188300* | 5 | 11147152 | 0.36433 | 0.034135 | AT1G22930.2 |  |
| *Aqcoe5G193300* | 5 | 11469856 | 0.36995 | 0.0012688 | AT4G34500.1 |  |
| *Aqcoe5G195700* | 5 | 11677501 | 0.38891 | 0.0032793 | AT5G42950.1 |  |
| *Aqcoe5G196900* | 5 | 11750135 | 0.31492 | 0.034772 | AT1G45160.2 |  |
| *Aqcoe5G198800* | 5 | 11883920 | 0.34892 | 0.026942 | AT4G36800.1 | RCE1 |
| *Aqcoe5G210900* | 5 | 12900417 | 0.46683 | 0 | AT1G75780.1 | TUB1 |
| *Aqcoe5G215200* | 5 | 13237537 | 0.33595 | 0.011945 | AT4G10960.1 | UGE5 |
| *Aqcoe5G224200* | 5 | 14445927 | 0.34602 | 0.014223 | AT4G39660.1 | AGT2 |
| *Aqcoe5G234100* | 5 | 15288901 | 0.38681 | 0.0047573 | AT3G21470.1 |  |
| *Aqcoe5G240000* | 5 | 16170786 | 0.37353 | 0.028582 | AT1G77122.1 |  |
| *Aqcoe5G249000* | 5 | 17371942 | 0.41108 | 0.0006922 |  |  |
| *Aqcoe5G252800* | 5 | 17702364 | 0.41868 | 0.00032942 | AT5G64750.1 | ABR1 |
| *Aqcoe5G257400* | 5 | 18157978 | 0.31857 | 0.02589 | AT3G56850.1 | AREB3 |
| *Aqcoe5G268600* | 5 | 20324986 | 0.39703 | 0.014445 | AT5G49580.1 |  |
| *Aqcoe5G269600* | 5 | 20597155 | 0.32925 | 0.020005 | AT1G47570.1 |  |
| *Aqcoe5G278500* | 5 | 22564169 | 0.37016 | 0.012473 | AT4G08500.1 | ARAKIN |
| *Aqcoe5G284100* | 5 | 23462236 | 0.40252 | 0.0055575 | AT1G21690.1 | EMB1968 |
| *Aqcoe5G288300* | 5 | 24000924 | 0.33043 | 0.042172 | AT4G01660.1 | ABC1 |
| *Aqcoe5G295300* | 5 | 25264543 | 0.51739 | 0 | AT5G18525.1 |  |
| *Aqcoe5G295500* | 5 | 25302183 | 0.5791 | 0 |  |  |
| *Aqcoe5G296000* | 5 | 25395536 | 0.5784 | 0 | AT3G17365.1 |  |
| *Aqcoe5G296100* | 5 | 25413672 | 0.37101 | 0.0011448 | AT3G17360.1 | POK1 |
| *Aqcoe5G297100* | 5 | 25741247 | 0.53329 | 0 |  |  |
| *Aqcoe5G297200* | 5 | 25785076 | 0.55943 | 0 | AT5G23630.1 | MIA |
| *Aqcoe5G297300* | 5 | 25818481 | 0.46171 | 0 | AT1G48100.1 |  |
| *Aqcoe5G297900* | 5 | 25927047 | 0.41673 | 1.43E-03 | AT1G09380.1 |  |
| *Aqcoe5G300900* | 5 | 26707248 | 0.39181 | 0.011527 | AT1G71220.2 | EBS1 |
| *Aqcoe5G310300* | 5 | 29449230 | 0.39596 | 0.00020678 | AT5G51070.1 | CLPD |
| *Aqcoe5G319500* | 5 | 30808437 | 0.42221 | 0 | AT3G02070.1 |  |
| *Aqcoe5G320600* | 5 | 31009828 | 0.36295 | 0.0036655 | AT1G44900.1 | MCM2 |
| *Aqcoe5G329400* | 5 | 32267062 | 0.42226 | 0.00032942 |  |  |
| *Aqcoe5G339000* | 5 | 34036493 | 0.32391 | 0.021022 | AT5G18740.1 |  |
| *Aqcoe5G340500* | 5 | 34196018 | 0.44737 | 8.49E-05 | AT5G23680.1 |  |
| *Aqcoe5G342400* | 5 | 34307496 | 0.37953 | 0.0058904 | AT1G54570.1 |  |
| *Aqcoe5G342900* | 5 | 34405295 | 0.34321 | 0.033067 | AT1G33410.2 | NUP160 |
| *Aqcoe5G346900* | 5 | 34858473 | 0.41818 | 0.00027922 | AT1G27430.1 |  |
| *Aqcoe5G351400* | 5 | 35335822 | 0.3333 | 0.014945 | AT1G67320.2 |  |
| *Aqcoe5G353400* | 5 | 35476897 | 0.31816 | 0.0285 | AT1G27320.1 | AHK3 |
| *Aqcoe5G354000* | 5 | 35526464 | 0.4317 | 0.00020678 | AT5G53150.1 |  |
| *Aqcoe5G362700* | 5 | 36356419 | 0.34404 | 0.030748 | AT4G16440.1 |  |
| *Aqcoe5G366300* | 5 | 36725759 | 0.38671 | 0.015691 | AT1G04920.1 | SPS3F |
| *Aqcoe5G381400* | 5 | 37875810 | 0.3609 | 0.015402 | AT2G04410.1 |  |
| *Aqcoe5G395600* | 5 | 38987588 | 0.38445 | 0.0031712 | AT4G24730.4 |  |
| *Aqcoe5G397600* | 5 | 39101283 | 0.47952 | 8.49E-05 | AT1G67850.1 |  |
| *Aqcoe5G398900* | 5 | 39202579 | 0.35117 | 0.023421 | AT2G45350.1 | CRR4 |
| *Aqcoe5G400000* | 5 | 39249256 | 0.40033 | 0.0023486 |  |  |
| *Aqcoe5G400600* | 5 | 39278159 | 0.33581 | 0.014778 |  |  |
| *Aqcoe5G414700* | 5 | 40360677 | 0.41949 | 0.0004029 | AT1G15110.1 |  |
| *Aqcoe5G432800* | 5 | 41692587 | 0.31629 | 0.03333 | AT4G12300.1 | CYP706A4 |
| *Aqcoe5G437600* | 5 | 42017038 | 0.39572 | 0.0017348 | AT4G11280.1 | ACS6 |
| *Aqcoe5G439400* | 5 | 42125217 | 0.39011 | 0.00088163 | AT5G24030.1 | SLAH3 |
| *Aqcoe6G015000* | 6 | 833736 | 0.33644 | 0.035327 | AT1G35510.1 |  |
| *Aqcoe6G022900* | 6 | 1241924 | 0.30307 | 0.047262 | AT1G77580.2 |  |
| *Aqcoe6G025000* | 6 | 1362815 | 0.42351 | 8.49E-05 | AT1G21740.1 |  |
| *Aqcoe6G031100* | 6 | 1650159 | 0.41407 | 0.0027823 | AT4G33280.1 |  |
| *Aqcoe6G038000* | 6 | 2023636 | 0.3635 | 0.013138 | AT1G21450.1 | SCL1 |
| *Aqcoe6G041100* | 6 | 2149797 | 0.34926 | 0.0063521 | AT5G09790.2 | XR5 |
| *Aqcoe6G044600* | 6 | 2330276 | 0.37406 | 0.008698 | AT3G10690.1 | GYRA |
| *Aqcoe6G049700* | 6 | 2611488 | 0.32114 | 0.047947 | AT1G14290.1 | SBH2 |
| *Aqcoe6G050000* | 6 | 2628641 | 0.39547 | 0.0017892 | AT3G19740.1 |  |
| *Aqcoe6G051700* | 6 | 2708214 | 0.37677 | 0.0062286 | AT5G64860.1 | DPE1 |
| *Aqcoe6G056100* | 6 | 2913667 | 0.34265 | 0.007121 | AT2G13810.1 | ALD1 |
| *Aqcoe6G059600* | 6 | 3104738 | 0.32878 | 0.017588 | AT2G17030.1 |  |
| *Aqcoe6G059900* | 6 | 3120679 | 0.31601 | 0.027349 | AT4G13830.2 | J20 |
| *Aqcoe6G065500* | 6 | 3474312 | 0.37927 | 0.007121 |  |  |
| *Aqcoe6G066600* | 6 | 3558039 | 0.38113 | 0.00088163 | AT5G64470.3 | TBL12 |
| *Aqcoe6G071500* | 6 | 3837741 | 0.36124 | 0.012827 | AT4G24450.1 | GWD2 |
| *Aqcoe6G074300* | 6 | 3959877 | 0.3962 | 0.002403 | AT2G36690.1 |  |
| *Aqcoe6G084600* | 6 | 4526081 | 0.3296 | 0.039983 | AT1G78190.1 |  |
| *Aqcoe6G084700* | 6 | 4527538 | 0.40636 | 0.00077267 |  |  |
| *Aqcoe6G085400* | 6 | 4574250 | 0.333 | 0.037406 | AT1G22275.1 | ZYP1 |
| *Aqcoe6G095800* | 6 | 5184916 | 0.34883 | 0.0065702 |  |  |
| *Aqcoe6G104600* | 6 | 5608895 | 0.35296 | 0.006696 | AT1G49410.1 | TOM6 |
| *Aqcoe6G118900* | 6 | 6486263 | 0.38722 | 4.49E-03 | AT1G19400.2 |  |
| *Aqcoe6G119700* | 6 | 6538972 | 0.41363 | 0.00046073 | AT4G36630.1 | EMB2754 |
| *Aqcoe6G130600* | 6 | 7235671 | 0.37688 | 0.0085131 | AT1G75450.1 | CKX5 |
| *Aqcoe6G130700* | 6 | 7252719 | 0.45145 | 0 | AT1G66980.1 | SNC4 |
| *Aqcoe6G135700* | 6 | 7606742 | 0.32399 | 0.044127 | AT2G23380.1 | CLF |
| *Aqcoe6G135900* | 6 | 7618547 | 0.38091 | 0.00083869 | AT5G53390.1 |  |
| *Aqcoe6G139100* | 6 | 7860344 | 0.37491 | 0.0013972 | AT4G36950.1 | MAPKKK21 |
| *Aqcoe6G139200* | 6 | 7860344 | 0.37491 | 0.0013972 | AT1G76280.3 |  |
| *Aqcoe6G143400* | 6 | 8132775 | 0.36099 | 0.016272 | AT1G66370.1 | MYB113 |
| *Aqcoe6G146700* | 6 | 8343070 | 0.30585 | 0.040703 | AT2G26560.1 | PLA IIA |
| *Aqcoe6G150100* | 6 | 8588728 | 0.56967 | 0 | AT3G63470.1 | scpl40 |
| *Aqcoe6G153300* | 6 | 8786886 | 0.40754 | 8.49E-05 | AT5G10470.1 | KAC1 |
| *Aqcoe6G153400* | 6 | 8807315 | 0.34187 | 0.017346 | AT5G27650.1 |  |
| *Aqcoe6G154100* | 6 | 8833515 | 0.38339 | 0.0010256 | AT4G24550.2 |  |
| *Aqcoe6G154400* | 6 | 8869061 | 0.39746 | 0.0015927 | AT4G38090.1 |  |
| *Aqcoe6G161300* | 6 | 9530360 | 0.42142 | 0.002477 | AT1G50030.1 | TOR |
| *Aqcoe6G167600* | 6 | 10114660 | 0.41835 | 0.00032942 | AT2G17480.1 | MLO8 |
| *Aqcoe6G170100* | 6 | 10364595 | 0.36024 | 0.0044062 | AT3G52320.1 |  |
| *Aqcoe6G178200* | 6 | 11144166 | 0.31856 | 0.029744 | AT5G42500.1 |  |
| *Aqcoe6G181300* | 6 | 11541286 | 0.307 | 0.04228 | AT1G57790.1 |  |
| *Aqcoe6G186200* | 6 | 11923861 | 0.32223 | 0.025256 | AT1G19250.1 | FMO1 |
| *Aqcoe6G197400* | 6 | 13215858 | 0.37537 | 0.0010935 | AT1G47670.1 |  |
| *Aqcoe6G200500* | 6 | 13747765 | 0.3908 | 0.011712 |  |  |
| *Aqcoe6G203900* | 6 | 14255225 | 0.43642 | 0 | AT2G17760.1 |  |
| *Aqcoe6G207900* | 6 | 15050504 | 0.37919 | 0.0072576 | AT1G19920.1 | APS2 |
| *Aqcoe6G213400* | 6 | 16183544 | 0.35027 | 0.0076424 | AT1G11000.1 | MLO4 |
| *Aqcoe6G214500* | 6 | 16451262 | 0.4331 | 0.0005713 | AT1G20160.1 | SBT5.2 |
| *Aqcoe6G221400* | 6 | 17944883 | 0.35083 | 0.044789 | AT5G35980.1 | YAK1 |
| *Aqcoe6G221900* | 6 | 17980282 | 0.39 | 0.016509 | AT2G16730.1 | BGAL13 |
| *Aqcoe6G222500* | 6 | 18107155 | 0.36135 | 1.47E-02 | AT5G66150.1 |  |
| *Aqcoe6G223100* | 6 | 18272898 | 0.30485 | 4.19E-02 | AT5G66140.1 | PAD2 |
| *Aqcoe6G244500* | 6 | 21572014 | 0.4004 | 0.01598 | AT2G18060.1 | ANAC037 |
| *Aqcoe6G267500* | 6 | 24421852 | 0.32152 | 0.049779 | AT1G59520.1 | CW7 |
| *Aqcoe6G267900* | 6 | 24477829 | 0.3539 | 0.0078591 | AT5G48930.1 | HCT |
| *Aqcoe6G277200* | 6 | 25753485 | 0.34424 | 0.023196 | AT1G10200.1 | WLIM1 |
| *Aqcoe6G288900* | 6 | 27016960 | 0.36793 | 0.0017348 |  |  |
| *Aqcoe6G324800* | 6 | 29989132 | 0.33635 | 0.035606 | AT1G76520.1 |  |
| *Aqcoe6G328500* | 6 | 30305830 | 0.36581 | 0.010764 | AT5G65930.2 | KCBP |
| *Aqcoe7G000700* | 7 | 78317 | 0.40386 | 0.00077267 | AT1G32810.2 |  |
| *Aqcoe7G001700* | 7 | 139061 | 0.40609 | 0.0013106 | AT2G46710.1 |  |
| *Aqcoe7G001800* | 7 | 164718 | 0.32166 | 0.049894 | AT2G46700.1 | CRK3 |
| *Aqcoe7G011200* | 7 | 721150 | 0.35114 | 0.0056174 |  |  |
| *Aqcoe7G013400* | 7 | 878478 | 0.36319 | 0.031942 | AT5G53160.2 | PYL8 |
| *Aqcoe7G014400* | 7 | 917572 | 0.35516 | 0.0041187 | AT4G01037.1 | WTF1 |
| *Aqcoe7G016400* | 7 | 1009163 | 0.32056 | 0.048635 |  |  |
| *Aqcoe7G016900* | 7 | 1062640 | 0.33477 | 0.033774 |  |  |
| *Aqcoe7G017100* | 7 | 1073202 | 0.36545 | 0.0048117 | AT3G61640.1 | AGP20 |
| *Aqcoe7G031100* | 7 | 1852105 | 0.39797 | 0.007326 | AT2G45910.1 |  |
| *Aqcoe7G031800* | 7 | 1903780 | 0.30639 | 0.04091 | AT4G00440.1 |  |
| *Aqcoe7G043100* | 7 | 2527708 | 0.45394 | 0.00032942 | AT5G63890.2 | HDH |
| *Aqcoe7G049900* | 7 | 2939455 | 0.31277 | 0.037599 | AT1G12600.1 |  |
| *Aqcoe7G053600* | 7 | 3237236 | 0.46365 | 0 | AT5G51830.1 |  |
| *Aqcoe7G058900* | 7 | 3581112 | 0.32273 | 0.023048 | AT2G45510.1 | CYP704A2 |
| *Aqcoe7G059500* | 7 | 3602320 | 0.36726 | 0.026778 |  |  |
| *Aqcoe7G060800* | 7 | 3692563 | 0.30734 | 0.041745 | AT4G37740.1 | GRF2 |
| *Aqcoe7G061700* | 7 | 3741209 | 0.40672 | 0.00095284 | AT2G45510.1 | CYP704A2 |
| *Aqcoe7G061800* | 7 | 3747723 | 0.33755 | 0.012674 | AT2G45510.1 | CYP704A2 |
| *Aqcoe7G072700* | 7 | 4357961 | 0.38612 | 0.0005713 | AT3G60750.1 |  |
| *Aqcoe7G080100* | 7 | 4776720 | 0.42646 | 0.0005713 | AT4G22540.1 | ORP2A |
| *Aqcoe7G084600* | 7 | 5094106 | 0.33355 | 0.017226 | AT1G02080.2 |  |
| *Aqcoe7G088300* | 7 | 5341595 | 0.39248 | 0.0020133 | AT5G04620.2 | BIOF |
| *Aqcoe7G125200* | 7 | 7774805 | 0.30879 | 0.037213 | AT2G26180.1 | IQD6 |
| *Aqcoe7G127200* | 7 | 7883854 | 0.36095 | 0.013297 | AT4G10620.1 |  |
| *Aqcoe7G131400* | 7 | 8171950 | 0.31964 | 0.035141 |  |  |
| *Aqcoe7G153700* | 7 | 10259284 | 0.41021 | 0.0019363 | AT5G13980.2 |  |
| *Aqcoe7G154800* | 7 | 10487996 | 0.40148 | 0.001375 | AT5G44400.1 |  |
| *Aqcoe7G168600* | 7 | 11728781 | 0.42871 | 4.06E-05 | AT5G11530.1 | EMF1 |
| *Aqcoe7G179500* | 7 | 12710197 | 0.36656 | 0.011805 | AT4G32700.2 | TEB |
| *Aqcoe7G185200* | 7 | 13419019 | 0.36004 | 0.016213 | AT2G29760.1 | OTP81 |
| *Aqcoe7G185300* | 7 | 13424986 | 0.32789 | 0.044789 |  |  |
| *Aqcoe7G199100* | 7 | 15257522 | 0.38288 | 0.011028 | AT1G15550.1 | GA3OX1 |
| *Aqcoe7G200500* | 7 | 15361957 | 0.34632 | 0.0085498 |  |  |
| *Aqcoe7G202600* | 7 | 15641928 | 0.37439 | 0.0076073 | AT1G15520.1 | ABCG40 |
| *Aqcoe7G203800* | 7 | 15726395 | 0.37509 | 0.0059516 | AT2G03500.1 |  |
| *Aqcoe7G209400* | 7 | 16412866 | 0.34044 | 0.010852 | AT3G15610.1 |  |
| *Aqcoe7G210600* | 7 | 16507205 | 0.3853 | 0.018018 | AT5G18390.1 |  |
| *Aqcoe7G211300* | 7 | 16602686 | 0.3421 | 0.031771 |  |  |
| *Aqcoe7G211700* | 7 | 16669731 | 0.38479 | 0.0023665 | AT5G25450.1 |  |
| *Aqcoe7G221400* | 7 | 19096076 | 0.41808 | 0.00064566 | AT4G28080.1 |  |
| *Aqcoe7G237900* | 7 | 21797699 | 0.38335 | 0.0005713 | AT5G35360.1 | CAC2 |
| *Aqcoe7G242200* | 7 | 22317371 | 0.30037 | 0.046693 | AT4G26000.1 | PEP |
| *Aqcoe7G247600* | 7 | 23063873 | 0.32058 | 0.028171 |  |  |
| *Aqcoe7G252800* | 7 | 23930745 | 0.32161 | 0.020953 | AT4G16630.1 |  |
| *Aqcoe7G254700* | 7 | 24433807 | 0.37119 | 0.032458 | AT5G57800.1 | CER3 |
| *Aqcoe7G266400* | 7 | 27069779 | 0.37353 | 0.0022428 |  |  |
| *Aqcoe7G281900* | 7 | 29859109 | 0.55488 | 0 | AT1G15740.1 |  |
| *Aqcoe7G282800* | 7 | 29932566 | 0.3363 | 0.012374 | AT2G41040.1 |  |
| *Aqcoe7G307500* | 7 | 33433972 | 0.31765 | 0.031257 | AT3G47570.1 |  |
| *Aqcoe7G322300* | 7 | 35111560 | 0.35699 | 0.04164 |  |  |
| *Aqcoe7G331700* | 7 | 35991701 | 0.3334 | 0.015287 | AT1G70000.2 |  |
| *Aqcoe7G333900* | 7 | 36187396 | 0.35088 | 0.020404 | AT2G24640.2 | UBP19 |
| *Aqcoe7G335000* | 7 | 36300190 | 0.2986 | 0.047604 | AT2G24610.1 | CNGC14 |
| *Aqcoe7G354200* | 7 | 37735528 | 0.30647 | 0.049093 | AT5G48930.1 | HCT |
| *Aqcoe7G355000* | 7 | 37793729 | 0.39786 | 0.0099377 | AT3G07870.1 |  |
| *Aqcoe7G370800* | 7 | 38916063 | 0.40472 | 0.0014557 |  |  |
| *Aqcoe7G394800* | 7 | 40678576 | 0.33155 | 0.040393 | AT5G19610.1 | GNL2 |
| *Aqcoe7G414400* | 7 | 41735168 | 0.50038 | 0 | AT2G17030.1 |  |
| *Aqcoe7G419300* | 7 | 41968394 | 0.42615 | 0.0012688 | AT4G29330.1 | DER1 |
| *Aqcoe7G423100* | 7 | 42222997 | 0.37679 | 0.0089214 | AT5G57040.1 |  |
| *Aqcoe7G425500* | 7 | 42374060 | 0.37195 | 0.0088839 | AT2G26170.1 | CYP711A1 |
| *Aqcoe7G426500* | 7 | 42429276 | 0.30003 | 0.049779 | AT1G54270.1 | EIF4A-2 |
| *Aqcoe7G436500* | 7 | 43069849 | 0.35143 | 0.006633 | AT1G19250.1 | FMO1 |
| *Aqcoe7G441900* | 7 | 43380776 | 0.40542 | 8.49E-05 | AT3G10710.1 | RHS12 |
| *Aqcoe7G443200* | 7 | 43484047 | 0.44087 | 0.0000406 | AT2G40030.1 | NRPD1B |

**Table S11. Pollinator insects assemblages and visitation frequency at different location.**

| **Order** | **Family** | **Genus** | **Species** | **Visitation**  **frequency (%)** | **Average**  **visitation rates (s)** | **Lineage** |
| --- | --- | --- | --- | --- | --- | --- |
| Hymenoptera | Apidae | *Xylocopa* | *X. appendiculata* | 3.13% | 4.125 | CN |
| Hymenoptera | Halictidae | *Lasioglossum* | *L. villosulum* | 15.63% | 24.857 | CN |
| Hymenoptera | Apidae | *Eucera* | *E. floralia* | 65.63% | 6.955 | CN |
| Hymenoptera | Apidae | [*Ceratina*](https://en.wikipedia.org/wiki/Ceratina) | *C. iwatai* | 1.56% | 34.250 | CN |
| Hymenoptera | Apidae | [*Bombus*](https://en.wikipedia.org/wiki/Bombus_ignitus) | *B. ignitus* | 14.06% | 9.885 | CN |
| Hymenoptera | Halictidae | *Lasioglossum* | *L. morio* | 100% | 52.665 | NW |

**Table S12. The gene list correlation the environment.**

| **Gene name** | **SNP** | | **bf** | **ρ** | **Environmental factors** | **Best Arabidopsis hit** | **Gene name** |
| --- | --- | --- | --- | --- | --- | --- | --- |
|  | **Chromosome** | **Location** |  |  |  |  |  |
| *Aqcoe1G062100* | 1 | 3125627 | 5.90E+153 | 0.099006 | Bio_17 | AT5G17860.1 | CAX7 |
| *Aqcoe1G068500* | 1 | 3526624 | 2.93E+29 | 0.16596 | Bio_1 | AT1G04580.1 | AAO4 |
| *Aqcoe1G079800* | 1 | 4262636 | 1.35E-01 | 186.98 | Bio_3 | AT3G10640.1 | VPS60.1 |
| *Aqcoe1G103500* | 1 | 5552781 | 0.11854 | 1.216 | Bio_3 | AT2G38900.2 | PR-6 |
| *Aqcoe1G116400 ** | 1 | 6354128 | 1.07E+50 | 0.11259 | Bio_17 | AT5G06160.1 | ATO |
| *Aqcoe1G116400 ** | 1 | 6354128 | 0.11939 | 4.18E+73 | Bio_3 | AT5G06160.1 | ATO |
| *Aqcoe1G145800* | 1 | 8182541 | 0.10657 | 3.68E-01 | Bio_3 | AT2G39190.2 | ATH8 |
| *Aqcoe1G186100* | 1 | 10919181 | 1.75E+27 | 0.11973 | Bio_17 | AT1G17220.1 | FUG1 |
| *Aqcoe1G204300* | 1 | 12113497 | 5.17E+11 | 0.068053 | Bio_4 | AT4G21150.3 | HAP6 |
| *Aqcoe1G242300* | 1 | 15314497 | 1.48E+11 | 0.06728 | Bio_4 | AT2G29120.1 | GLR2.7 |
| *Aqcoe1G254100* | 1 | 16690488 | 1.35E-01 | 6089100 | Bio_3 | AT3G15190.1 |  |
| *Aqcoe1G298500* | 1 | 22970890 | 0.10569 | 3.29E+00 | Bio_3 | AT3G20440.2 | BE1 |
| *Aqcoe1G302700* | 1 | 23894535 | 0.11061 | 1293400 | Bio_3 | AT5G41950.1 |  |
| *Aqcoe1G315300* | 1 | 27495378 | 1.41E-01 | 1986400000 | Bio_3 | AT5G26960.1 |  |
| *Aqcoe1G341500* | 1 | 32135896 | 3.04E+67 | 9.23E-02 | Bio_17 | AT1G62360.1 | BUM |
| *Aqcoe1G351400* | 1 | 33506634 | 2.21E+137 | 0.064115 | Bio_4 |  |  |
| *Aqcoe1G444400* | 1 | 41268720 | 0.10675 | 2.75E+04 | Bio_3 | AT2G21440.1 |  |
| *Aqcoe1G500600* | 1 | 44606206 | 6.71E+30 | 0.10449 | Bio_17 | AT5G20920.1 | EIF2 BETA |
| *Aqcoe2G011000* | 2 | 803723 | 1.53E+24 | 0.16169 | Bio_1 | AT1G50480.1 | THFS |
| *Aqcoe2G017500* | 2 | 1233429 | 0.10416 | 0.30144 | Bio_3 | AT3G48880.2 |  |
| *Aqcoe2G053400* | 2 | 3596623 | 0.12316 | 1.35E+11 | Bio_3 |  |  |
| *Aqcoe2G066200* | 2 | 4655905 | 0.11804 | 0.67084 | Bio_3 | AT5G17980.1 |  |
| *Aqcoe2G111200* | 2 | 9003498 | 4.91E+26 | 0.15738 | Bio_1 | AT3G24495.1 | MSH7 |
| *Aqcoe2G113900* | 2 | 9310698 | 7.27E+29 | 0.16397 | Bio_1 | AT5G04760.1 |  |
| *Aqcoe2G156300* | 2 | 16393987 | 0.12495 | 1.1385 | Bio_3 | AT4G00380.1 |  |
| *Aqcoe2G186300* | 2 | 23100245 | 0.11824 | 3958000 | Bio_3 | AT3G52970.1 | CYP76G1 |
| *Aqcoe2G194000* | 2 | 23403889 | 1.19E+68 | 0.1777 | Bio_1 |  |  |
| *Aqcoe2G196600* | 2 | 23519111 | 1.20E-01 | 1.6352 | Bio_3 |  |  |
| *Aqcoe2G213700* | 2 | 26539224 | 1.15E-01 | 2.453 | Bio_3 | AT2G13680.1 | GSL02 |
| *Aqcoe2G256900* | 2 | 31530061 | 1.99E+21 | 0.098307 | Bio_17 | AT5G30510.1 | ARRPS1 |
| *Aqcoe2G258100* | 2 | 31595216 | 0.10571 | 1.69E+17 | Bio_3 |  |  |
| *Aqcoe2G290800* | 2 | 34518099 | 0.11071 | 4.75E+00 | Bio_3 | AT4G15920.1 |  |
| *Aqcoe2G344400* | 2 | 37814362 | 4.19E+12 | 6.21E-02 | Bio_4 | AT4G14930.1 |  |
| *Aqcoe2G422300* | 2 | 42209252 | 5.70E+30 | 0.062385 | Bio_4 |  |  |
| *Aqcoe3G006000* | 3 | 370029 | 5.24E+28 | 0.095142 | Bio_17 | AT1G70670.1 |  |
| *Aqcoe3G016500* | 3 | 1028621 | 7.49E+35 | 9.27E-02 | Bio_17 | AT1G57790.1 | ATFDR1 |
| *Aqcoe3G025400* | 3 | 1594439 | 1.48E-01 | 98295 | Bio_3 | AT1G54150.1 |  |
| *Aqcoe3G103800* | 3 | 6993511 | 3.07E+29 | 6.06E-02 | Bio_4 | AT3G48160.1 | DEL1 |
| *Aqcoe3G119200* | 3 | 8279408 | 1.01E+26 | 0.059109 | Bio_4 | AT5G13700.1 | APAO |
| *Aqcoe3G119800* | 3 | 8377290 | 1.10E-01 | 4.1278 | Bio_3 | AT3G47990.1 | SIS3 |
| *Aqcoe3G187100* | 3 | 19557660 | 0.10729 | 7.65E+04 | Bio_3 | AT1G31360.1 | RECQ2 |
| *Aqcoe3G212100* | 3 | 24079514 | 4.26E+48 | 1.46E-01 | Bio_1 | AT1G32090.1 |  |
| *Aqcoe3G232300* | 3 | 27739554 | 1.09E+49 | 7.62E-02 | Bio_4 | AT5G46800.1 | BOU |
| *Aqcoe3G374000* | 3 | 38672902 | 0.1236 | 8.53E-01 | Bio_3 | AT1G69120.1 | AGL7 |
| *Aqcoe3G387000* | 3 | 39486916 | 1.18E-01 | 0.28212 | Bio_3 | AT1G61600.1 |  |
| *Aqcoe4G012500* | 4 | 1129444 | 0.10335 | 5.32E+00 | Bio_3 | AT5G16360.1 |  |
| *Aqcoe4G026600* | 4 | 2313515 | 4.31E+52 | 0.079647 | Bio_4 | AT1G53440.1 |  |
| *Aqcoe4G098100* | 4 | 9798677 | 8.17E+30 | 0.14251 | Bio_1 |  |  |
| *Aqcoe4G213500* | 4 | 33297671 | 0.12038 | 3.89E+57 | Bio_3 | AT2G01050.1 |  |
| *Aqcoe4G228700* | 4 | 36491848 | 1.78E+30 | 0.14667 | Bio_1 | AT4G08850.1 |  |
| *Aqcoe4G245800* | 4 | 39581031 | 0.104 | 4.57E-01 | Bio_3 | AT4G05200.1 | CRK25 |
| *Aqcoe4G293200* | 4 | 44155649 | 5.29E+57 | 0.11421 | Bio_17 | AT2G42990.1 |  |
| *Aqcoe5G011700* | 5 | 710164 | 0.11043 | 1.8058 | Bio_3 | AT2G20330.1 |  |
| *Aqcoe5G097900* | 5 | 5251774 | 1.64E+111 | 0.15635 | Bio_1 |  |  |
| *Aqcoe5G166800* | 5 | 9658181 | 1.11E-01 | 7.86E+36 | Bio_3 | AT4G21220.1 |  |
| *Aqcoe5G207000* | 5 | 12599017 | 1.43E-01 | 2.36E+11 | Bio_3 | AT1G54490.1 | AIN1 |
| *Aqcoe5G215300* * | 5 | 13243073 | 1.29E+60 | 0.12223 | Bio_17 | AT4G25790.1 |  |
| *Aqcoe5G215300* * | 5 | 13243073 | 0.1228 | 7.78E+131 | Bio_3 | AT4G25790.1 |  |
| *Aqcoe5G261100* | 5 | 19133367 | 0.10443 | 0.38545 | Bio_3 | AT5G37590.1 |  |
| *Aqcoe5G299000* | 5 | 26137606 | 1.20E-01 | 1.13E+16 | Bio_3 | AT1G60940.1 | SNRK2-10 |
| *Aqcoe5G383000* | 5 | 37984262 | 3.40E+40 | 0.14902 | Bio_1 | AT5G26850.3 |  |
| *Aqcoe5G447600* | 5 | 42705640 | 1.32E-01 | 7.60E+21 | Bio_3 | AT1G32900.1 | GBSS1 |
| *Aqcoe6G083300* | 6 | 4438431 | 4.61E+24 | 0.1459 | Bio_1 | AT4G39952.1 |  |
| *Aqcoe6G086600* | 6 | 4628846 | 0.12164 | 8.23E+13 | Bio_3 | AT1G08070.1 | OTP82 |
| *Aqcoe6G087400* | 6 | 4667933 | 1.42E+258 | 1.20E-01 | Bio_17 | AT5G64170.1 |  |
| *Aqcoe6G230900* | 6 | 19555275 | 1.20E-01 | 6973.3 | Bio_3 | AT1G56450.1 | PBG1 |
| *Aqcoe6G261100* | 6 | 23861548 | 0.12156 | 1.52E+16 | Bio_3 | AT1G71770.1 | PAB5 |
| *Aqcoe6G261800* | 6 | 23907811 | 9.88E+50 | 0.098712 | Bio_17 | ATMG00090.1 |  |
| *Aqcoe6G275100* | 6 | 25502070 | 1.09E-01 | 3.19E+50 | Bio_3 | AT5G39030.1 |  |
| *Aqcoe7G000300* | 7 | 44779 | 1.06E-01 | 0.76956 | Bio_3 | AT5G53730.1 |  |
| *Aqcoe7G006800* | 7 | 426219 | 4.32E+69 | 0.10232 | Bio_17 | AT5G53060.1 |  |
| *Aqcoe7G046600* | 7 | 2739554 | 0.10397 | 6.60E+253 | Bio_3 | AT5G62090.1 | SLK2 |
| *Aqcoe7G060400* | 7 | 3668204 | 1.03E-01 | 9.0511 | Bio_3 | AT5G62220.1 | GT18 |
| *Aqcoe7G078500* | 7 | 4684508 | 1.17E-01 | 7200.7 | Bio_3 | AT4G12420.2 | SKU5 |
| *Aqcoe7G229300* * | 7 | 20347699 | 1.43E+56 | 0.15598 | Bio_1 | ATCG00150.1 |  |
| *Aqcoe7G229300* * | 7 | 20347699 | 0.18268 | 1.88E+72 | Bio_15 | ATCG00150.1 |  |
| *Aqcoe7G248400* * | 7 | 23114247 | 2.34E+88 | 1.06E-01 | Bio_17 | AT1G16710.1 | HAC12 |
| *Aqcoe7G248400 ** | 7 | 23114247 | 1.04E-01 | 9.42E+167 | Bio_3 | AT1G16710.1 | HAC12 |
| *Aqcoe7G266900* | 7 | 27177911 | 1.07E-01 | 4.70E+17 | Bio_3 | AT5G20150.1 | SPX1 |
| *Aqcoe7G274000* | 7 | 28397009 | 2.08E+29 | 0.14825 | Bio_1 | AT4G21470.1 | FMN/FHY |
| *Aqcoe7G281200* * | 7 | 29516773 | 2.04E+37 | 0.12308 | Bio_17 | AT1G80230.1 |  |
| *Aqcoe7G281200* * | 7 | 29516773 | 1.13E-01 | 1.75E+97 | Bio_3 | AT1G80230.1 |  |
| *Aqcoe7G362000* | 7 | 38250146 | 1.15E-01 | 0.38743 | Bio_3 | AT4G30993.2 |  |
| *Aqcoe7G369800* | 7 | 38826932 | 1.66E+53 | 9.90E-02 | Bio_17 | AT5G25220.1 | KNAT3 |
| *Aqcoe7G389000* | 7 | 40343972 | 1.10E-01 | 1.7743 | Bio_3 | AT5G11200.1 |  |
| *Aqcoe7G425300* * | 7 | 42365619 | 3.20E+33 | 0.11866 | Bio_17 | AT1G64280.1 | NPR1 |
| *Aqcoe7G425300* * | 7 | 42365619 | 0.12037 | 2.10E+51 | Bio_3 | AT1G64280.1 | NPR1 |
| *Aqcoe7G426800 ** | 7 | 42451779 | 4.90E+26 | 0.12915 | Bio_17 | AT5G11840.1 |  |
| *Aqcoe7G426800* * | 7 | 42451779 | 1.38E-01 | 1.40E+64 | Bio_3 | AT5G11840.1 |  |
| *Aqcoe7G442400* | 7 | 43424908 | 1.75E+11 | 0.077733 | Bio_4 | AT4G33150.2 | LKR |
