# Supplementary figures and images for: Geographic–genomic and geographic–phenotypic differentiation of the Aquilegia viridiflora complex
Source: Hortic Res. 2023 Mar 13;10(5):uhad041. doi: 10.1093/hr/uhad041 (PMC10163360; doi:10.1093/hr/uhad041)

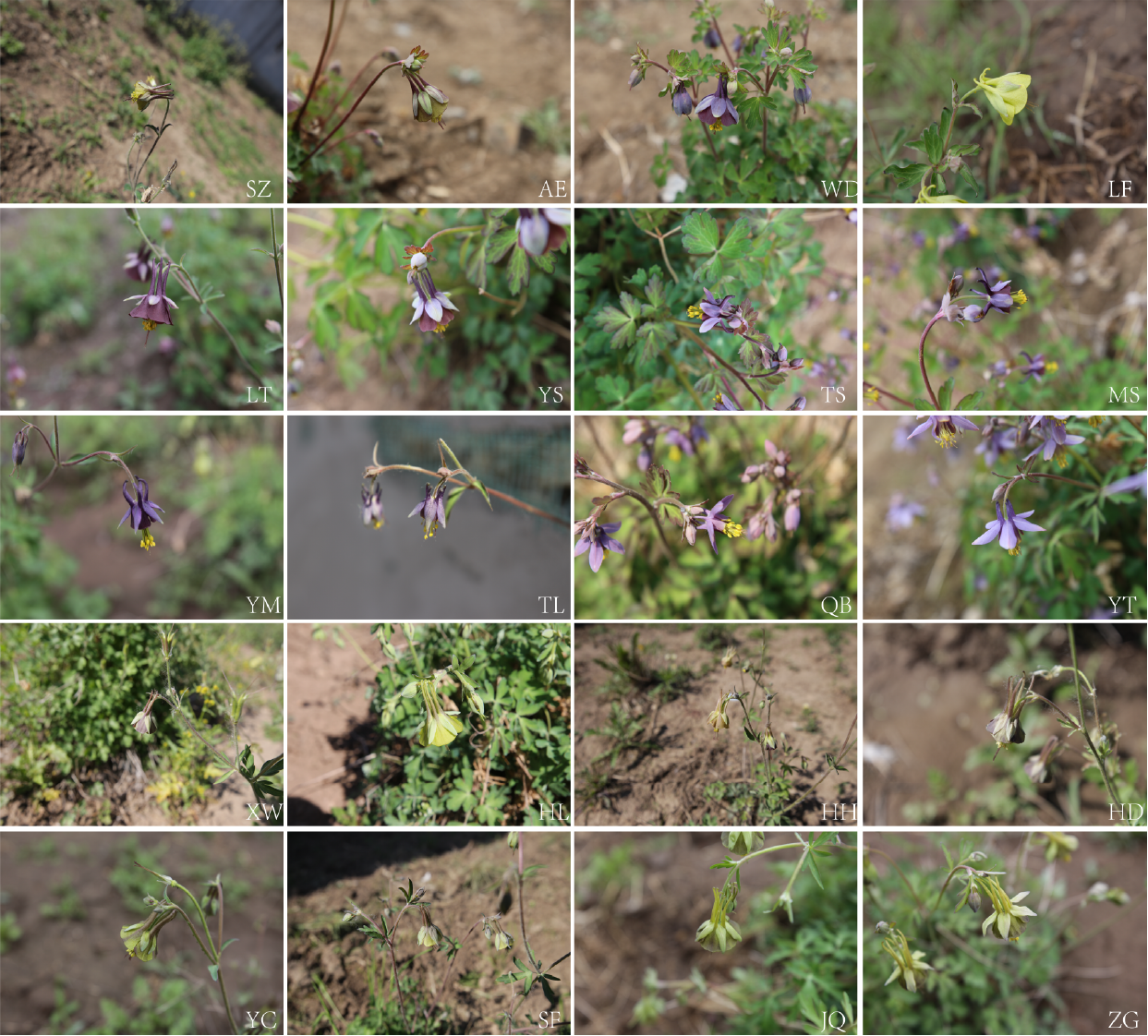

Supplement: Web_Material_uhad041 [file web_material_uhad041.zip › Figure_S1.tif]

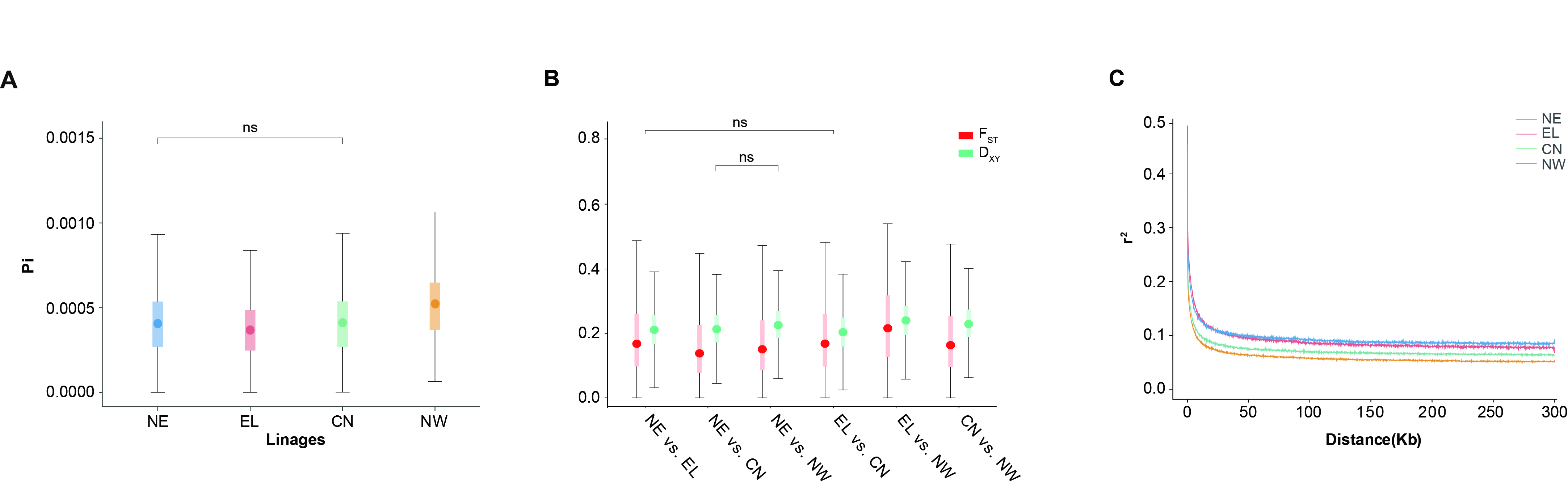

Supplement: Web_Material_uhad041 [file web_material_uhad041.zip › Figure_S10.tif]

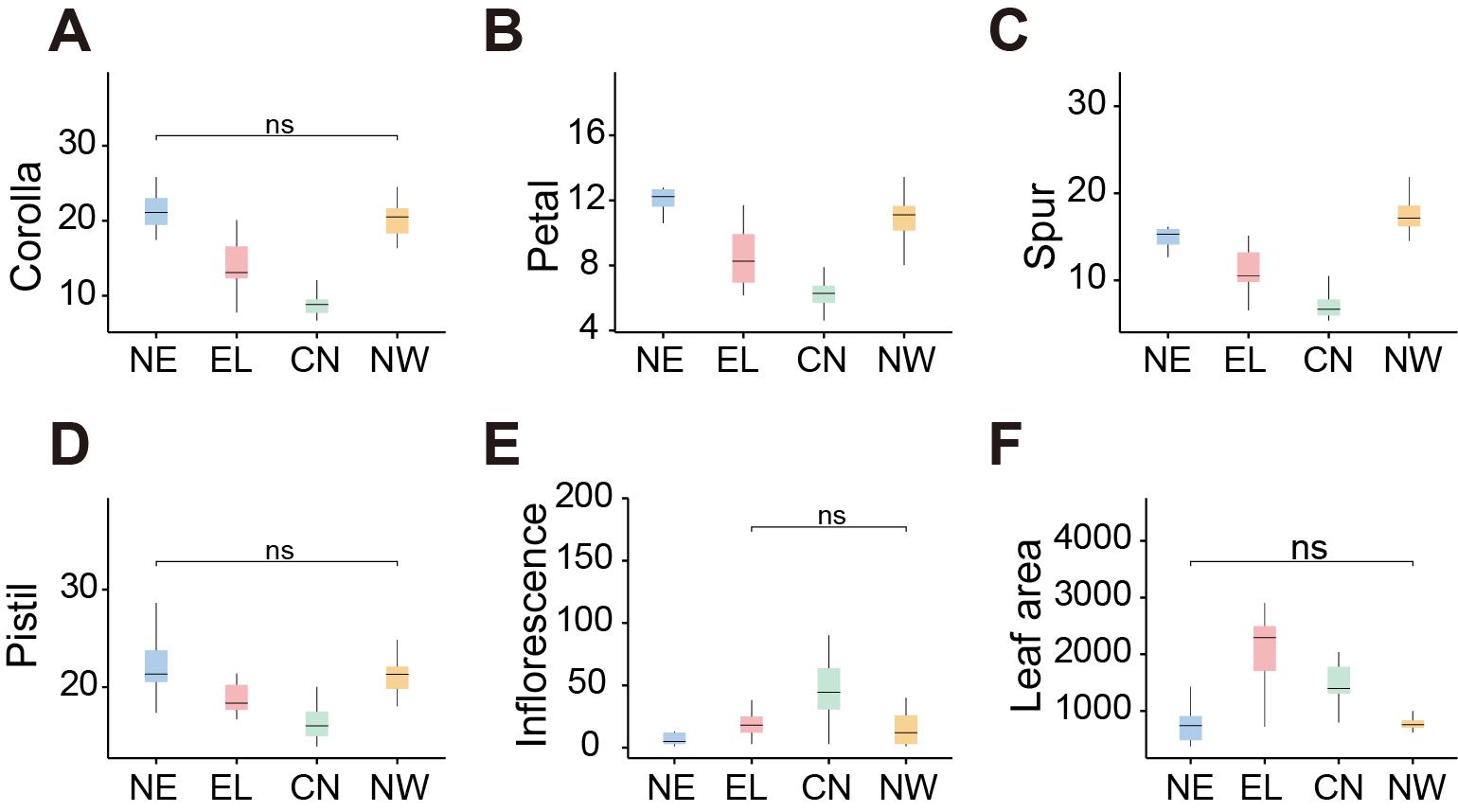

Supplement: Web_Material_uhad041 [file web_material_uhad041.zip › Figure_S11.tif]

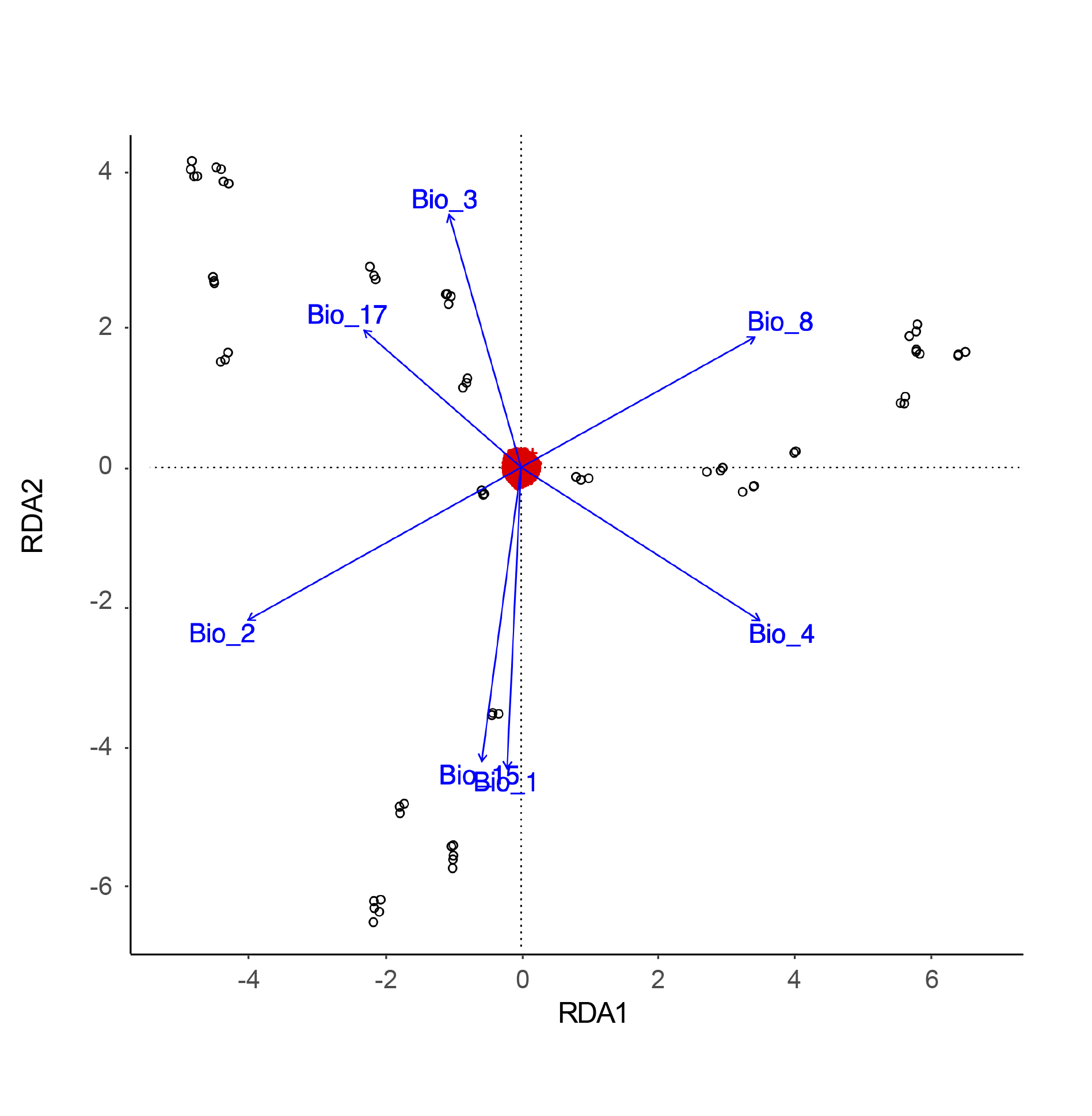

Supplement: Web_Material_uhad041 [file web_material_uhad041.zip › Figure_S12.tif]

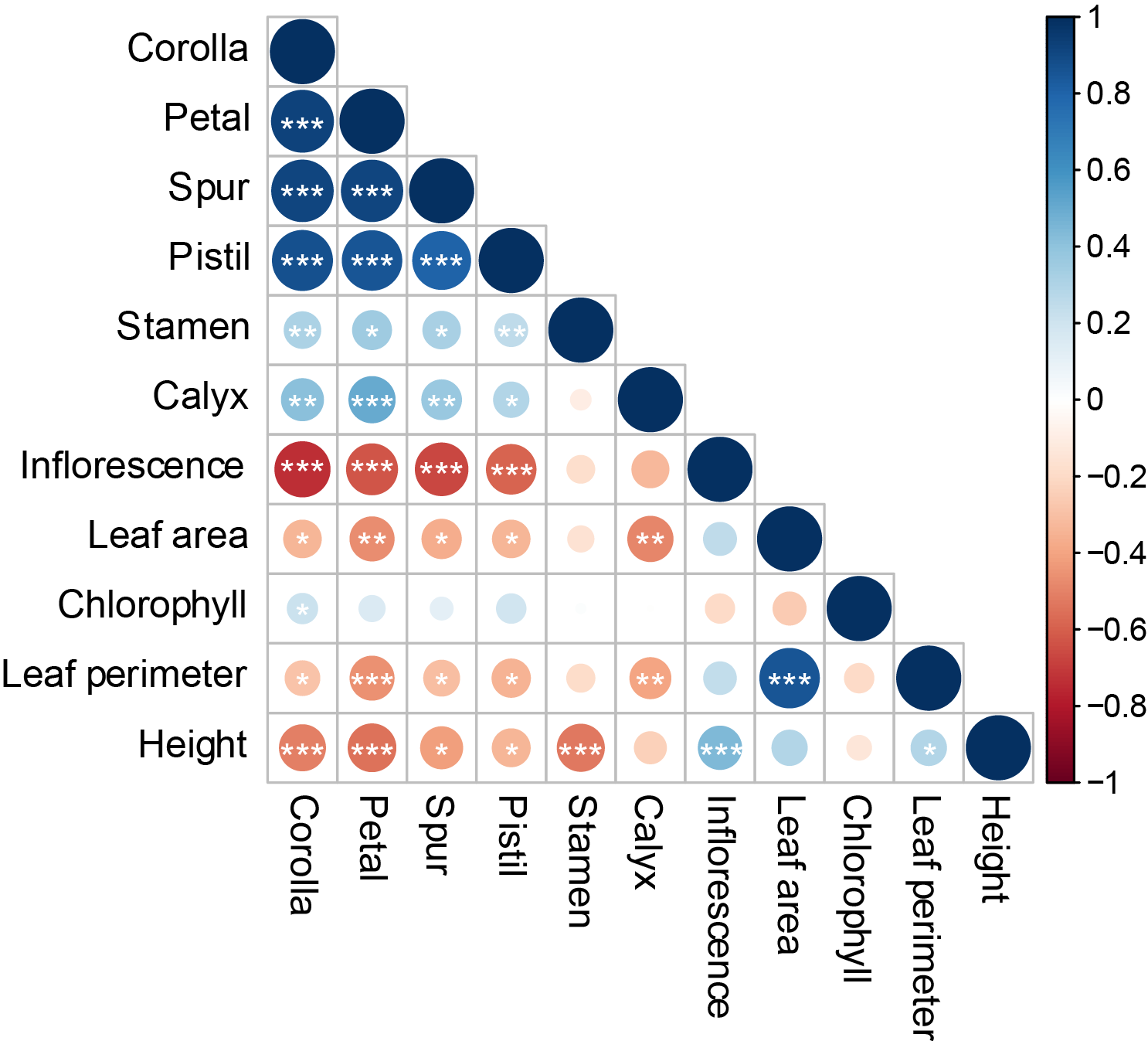

Supplement: Web_Material_uhad041 [file web_material_uhad041.zip › Figure_S2.tif]

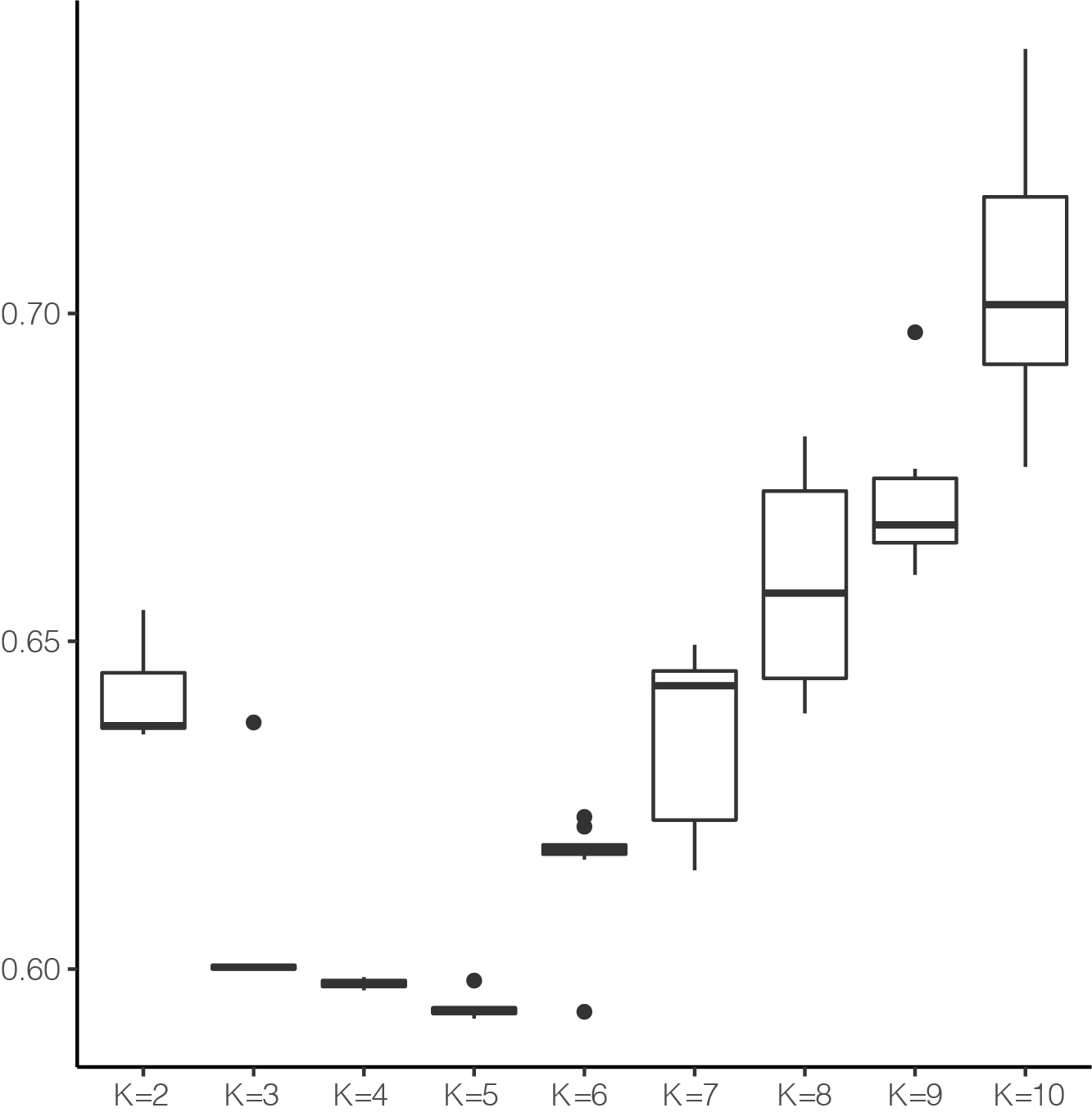

Supplement: Web_Material_uhad041 [file web_material_uhad041.zip › Figure_S3.tif]

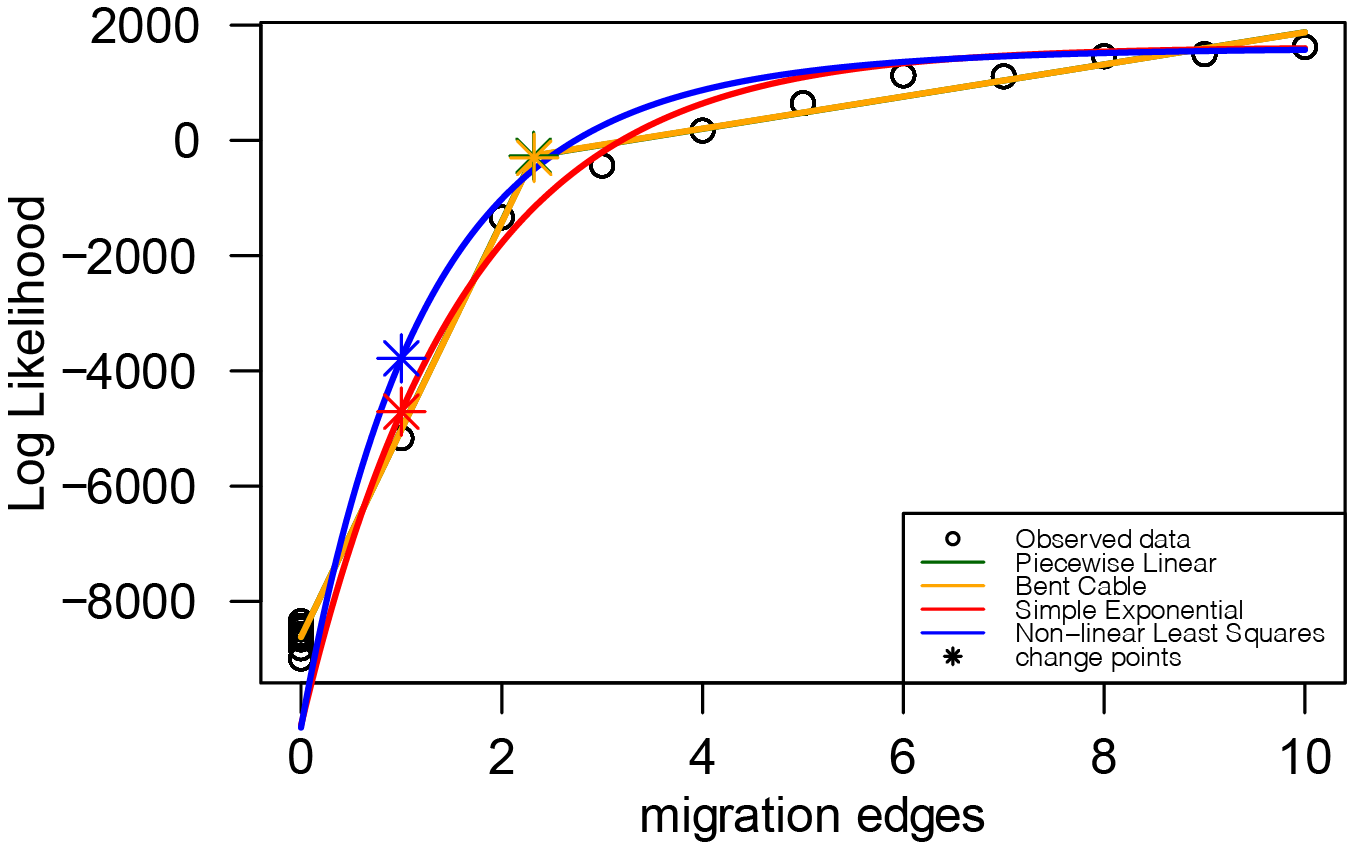

Supplement: Web_Material_uhad041 [file web_material_uhad041.zip › Figure_S4.tif]

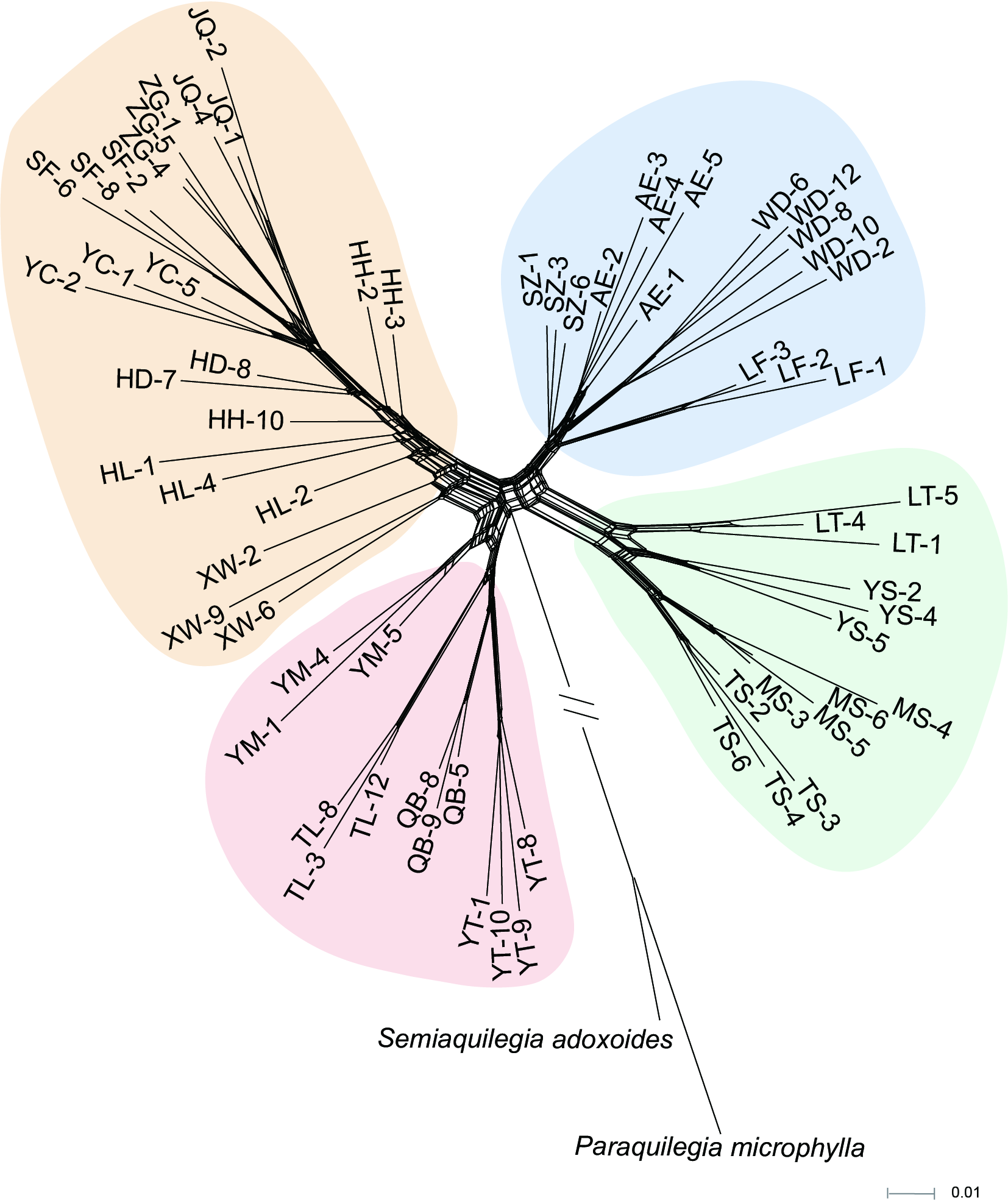

Supplement: Web_Material_uhad041 [file web_material_uhad041.zip › Figure_S5.tif]

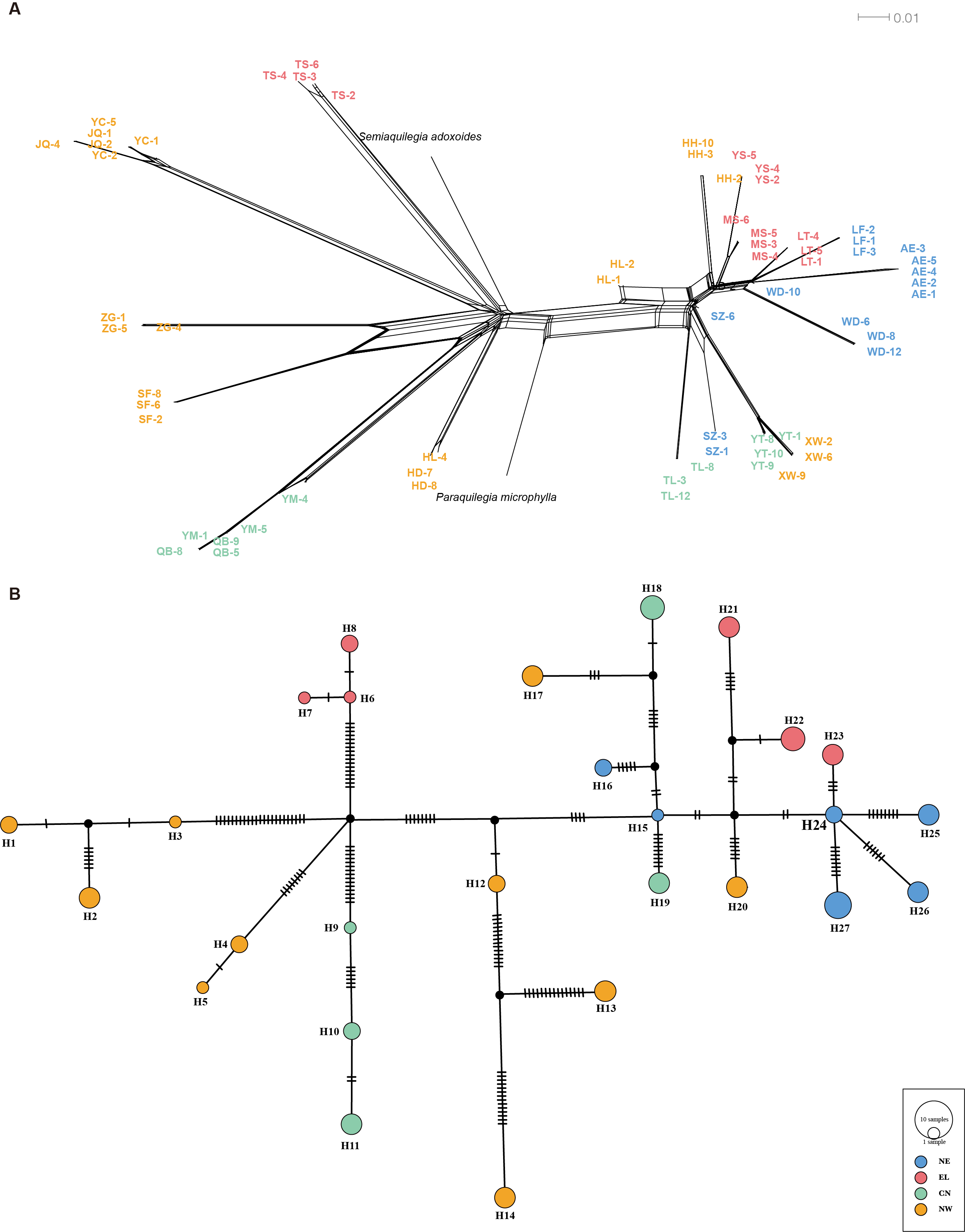

Supplement: Web_Material_uhad041 [file web_material_uhad041.zip › Figure_S6.tif]

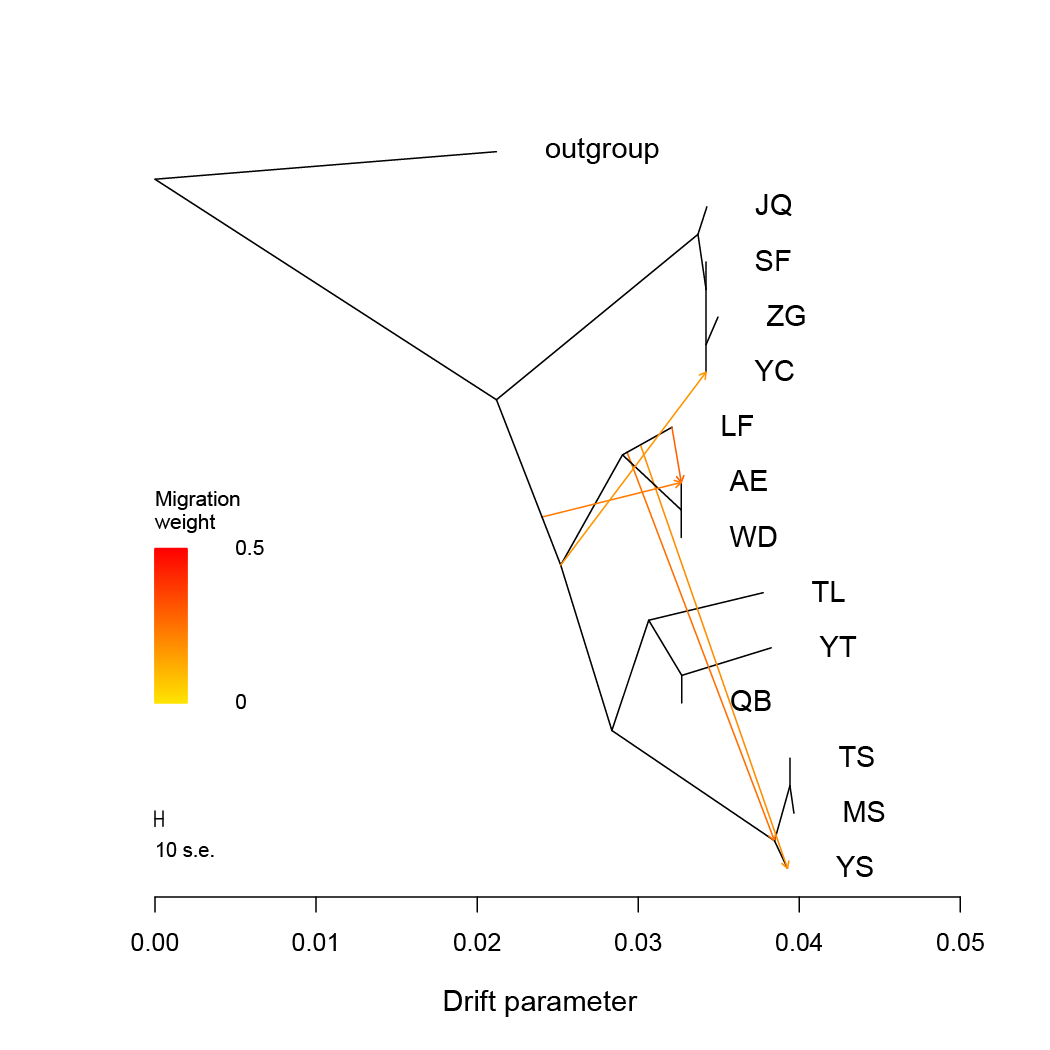

Supplement: Web_Material_uhad041 [file web_material_uhad041.zip › Figure_S7.tif]

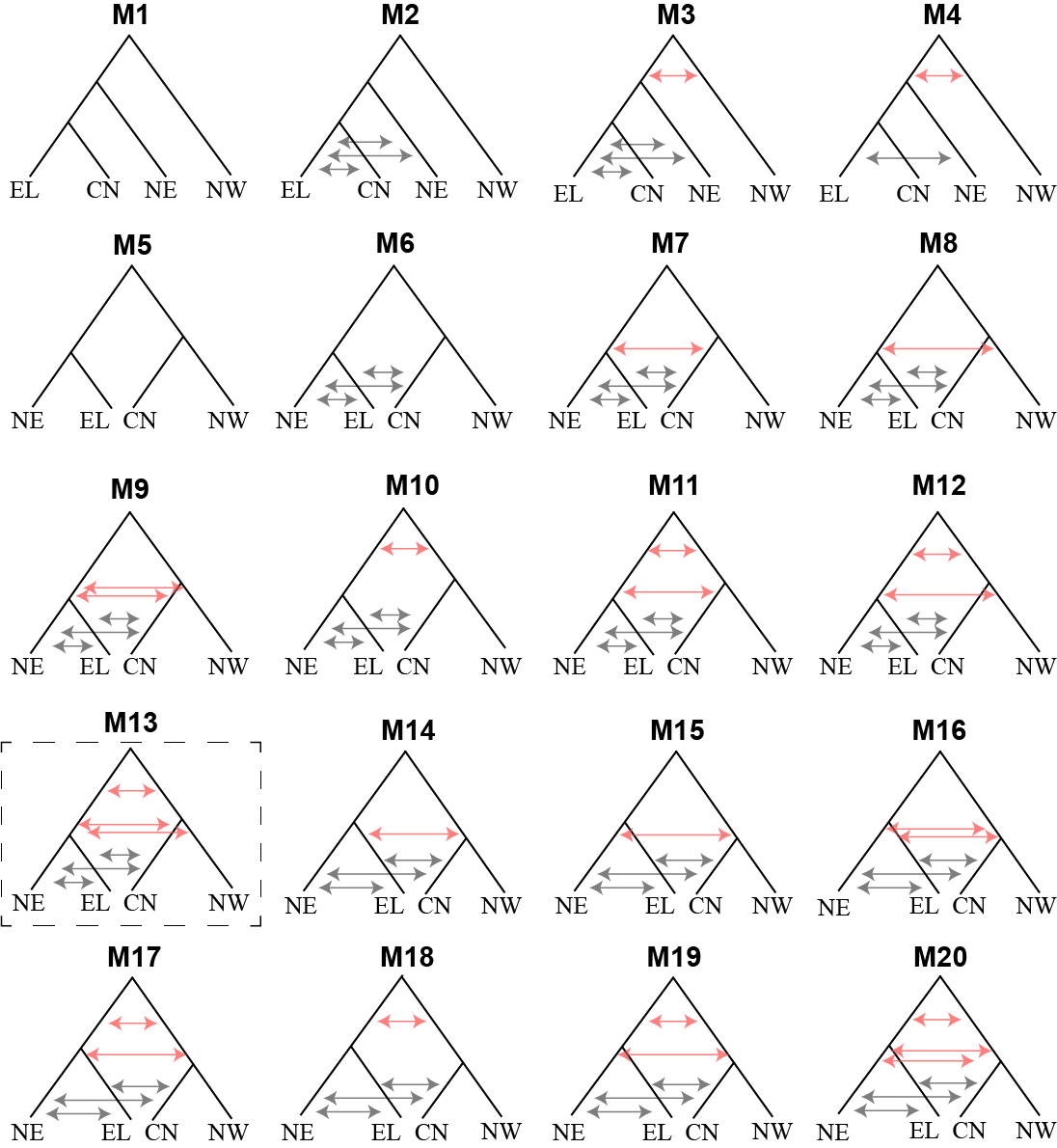

Supplement: Web_Material_uhad041 [file web_material_uhad041.zip › Figure_S8.tif]

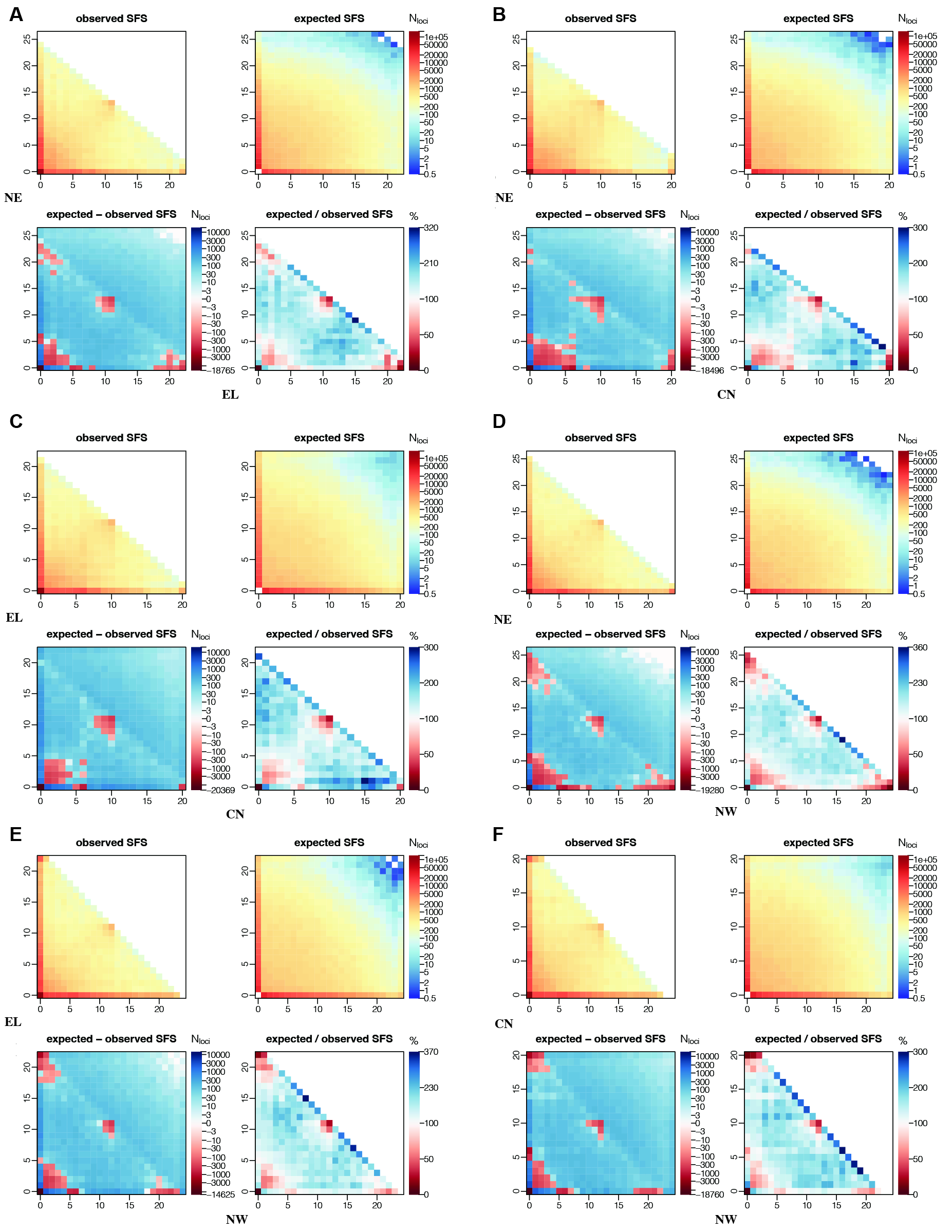

Supplement: Web_Material_uhad041 [file web_material_uhad041.zip › Figure_S9.tif]
